# Studying Noncovalent Interactions in Molecular Systems with Machine Learning

**DOI:** 10.1021/acs.chemrev.4c00893

**Published:** 2025-06-09

**Authors:** Serhii Tretiakov, AkshatKumar Nigam, Robert Pollice

**Affiliations:** † Stratingh Institute for Chemistry, 3647 University of Groningen, 9747 AG Groningen, The Netherlands; ‡ KlyneAI, San Francisco, California 94305, United States

## Abstract

Noncovalent interactions (NCIs) is an umbrella term for a multitude of typically weak interactions within and between molecules. Despite the low individual energy contributions, their collective effect significantly influences molecular behavior. Accordingly, understanding these interactions is crucial across fields like catalysis, drug design, materials science, and environmental chemistry. However, predicting NCIs is challenging, requiring at least molecular mechanics-level pairwise energy contributions or efficient quantum mechanical electron correlation treatment. In this review, we investigate the application of machine learning (ML) to study NCIs in molecular systems, an emerging research field. ML excels at modeling complex nonlinear relationships, and is capable of integrating vast data sets from experimental and theoretical sources. It offers a powerful approach for analyzing interactions across scales, from small molecules to large biomolecular assemblies. Specifically, we examine data sets characterizing NCIs, compare molecular featurization techniques, assess ML models predicting NCIs explicitly, and explore inverse design approaches. ML enhances predictive accuracy, reduces computational costs, and reveals overlooked interaction patterns. By identifying current challenges and future opportunities, we highlight how ML-driven insights could revolutionize this field. Overall, we believe that recent proof-of-concept studies foreshadow exciting developments for the study of NCIs in the years to come.

## Graphical abstract

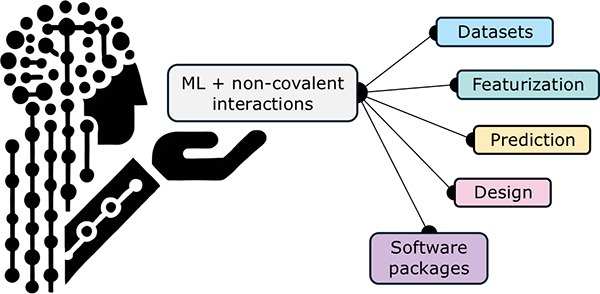

## 1 Introduction

Noncovalent interactions (NCIs) refer to usually weak attractive or repulsive forces occurring between separate atoms and within or between molecules, without significant electron sharing.^1^ Compared to covalent bonds, NCIs, whether intra- or intermolecular, are significantly weaker and have lower directionality.^1^ Individual interactions typically fall within the energy range of 1–5 kcal mol^–1^ but can reach over 40 kcal mol^–1^ in exceptional cases.^2–4^ While ionic bonds in solids are technically a type of NCIs, their high interaction strength and primary role in forming these compounds^5^ set them apart. In this review, we focus on weaker NCIs and therefore do not cover strong ionic bonds in solid crystals, which have been discussed elsewhere.^6^

NCIs are fundamental to nearly every substance, shaping the physical and chemical properties of matter. They govern intermolecular forces that affect reactivity, phase behavior, solubility, and molecular organization, thus playing critical role in complex chemical and biological processes. In the gas phase, weak noncovalent forces govern the behavior of nonideal gases,^7,8^ impact collision outcomes,^9^ explain rich and otherwise hard-to-assign microwave and infrared spectra,^10^ and drive processes in the atmosphere and interstellar space.^11^ In condensed phase, NCIs are essential for the existence of molecular liquids and crystals,^12–16^ and understanding them is key for accurately predicting crystal structures and determining relative polymorph stability.^17,18^ NCIs also drive physisorption,^19^ dictating how molecules bind to surfaces and within porous materials. They play a crucial role in solvation,^20^ influencing molecular properties, spectra, and reactivity. In catalyst design, especially for enantioselective reactions,^21^ these forces directly impact the stability of key reactive intermediates and transition states.

In biological systems, NCIs are indispensable, supporting protein structure, DNA formation,^22^ enzyme–substrate binding,^23^ and drug-target interactions.^24^ Their role in cellular function and drug design underscore their biological significance, as specific interactions can determine molecular recognition and influence therapeutic efficacy. Disruptions in these interactions can lead to deleterious effects, such as protein misfolding and aggregation, which are implicated in numerous diseases, including neurodegenerative disorders and cancers.^25,26^ Accordingly, given their ubiquity and critical roles, NCIs continue to be a major focus for experimentalists and theoreticians alike.

In recent years, machine learning (ML) has made a significant impact on chemistry and physical sciences, which was highlighted by the 2024 Nobel Prizes in Physics and Chemistry, awarded for groundbreaking advances in artificial neural networks and for innovations in predicting and designing protein structures, respectively.^27^ In chemistry, important applications of ML include property prediction based on molecular structure,^28–31^ inverse molecular design to attain the systems with desired properties,^32–36^ closed-loop optimization of experimental parameters or molecular properties,^37–39^ deducing the underlying structure in large molecular data sets along with utilizing it as a guide for molecular design,^40–42^ and the use of autonomous research agents via large action models.^43,44^ By integrating both experimental and computational data, ML enables rapid exploration of chemical space, making it increasingly valuable for advancing molecular science. Accordingly, ML has also made a significant impact in the study of NCIs. The abundance, diversity, and subtlety of NCIs makes them a challenging research object, but at the same time ideally suited for the inherently data-driven approaches of ML.

In this review, we provide a comprehensive overview of ML-assisted approaches for studying NCIs, restricting ourselves to small and midsized molecular systems. To avoid overlap with recent comprehensive reviews on ML for electronic structure theory^45–48^ and molecular force fields,^48,49^ we will focus on papers that either explicitly study NCIs through the use of ML, or enable such studies by providing systematic data sets that benchmark certain types of NCIs. Accordingly, this excludes the work on developing ML approaches with the primary purpose of replacing quantum chemistry or molecular force fields for computing electronic energies or molecular structures. This also excludes ML approaches to be used alongside electronic structure methods to correct for errors in the predicted electronic energy that do not make use of explicit NCI features. As mentioned above, we will not cover ionic bonding present in solid crystals either, even though it does formally belong to NCIs. For a recent review on the application of ML to crystals, including ionic crystals, we refer the reader to the relevant literature.^6^ After an overview of the specific classes of NCIs and ML architectures highlighted in this work, we divide the review into sections focusing on data sets for NCIs, NCIs as features for ML and predicting and designing NCIs with ML. We then proceed with a brief overview of the software packages that facilitate the study of NCIs with ML, followed by outlining the challenges in this research area. Finally, we provide an outlook on promising future applications, and conclude with a concise summary.

## 2 Overview of Noncovalent Interactions

NCIs are often nuanced, involving multiple appreciable interaction components, but, ultimately, can be broken down into the following widely invoked physical forces:^50–53^ electrostatic interactions (Coulomb forces), induction (Debye forces), dispersion (London forces),^54^ Pauli exchange,^55,56^ and charge transfer^57^ (without full covalent bond formation). The relative strength of each force may vary depending on the molecular environment,^58^ adding complexity to predicting and understanding NCIs across different systems. From combinations of these basic forces in different proportions, more complex interaction patterns arise, such as hydrogen bonding,^59^ halogen bonding,^60^ π-π interactions^61^ and others. In this section, we define some of the complex interactions that will be referenced throughout the review and how their physical interaction mechanisms can be elucidated computationally.

### 2.1 General Types of NCIs

The van der Waals (vdW) forces (Figure 1A) are nonlocal forces between molecules, which are classically defined as a combination of the interactions between two permanent dipoles (Keesom force), between a permanent dipole and an induced dipole (Debye force), between mutually induced dipoles (London dispersion),^62^ and repulsive interactions.^63,64^ The vdW forces are essential for stabilizing molecular conformations and influence molecular aggregation, especially in the condensed phase. The attractive component of the vdW interaction energy between two atoms asymptotically decays with the sixth power of the distance.^63^ While vdW forces always result in net attraction at sufficiently long interaction distances, the interaction becomes repulsive at short distances, governed by the Pauli exclusion principle.^65^ This interplay of attractive and repulsive interaction energy components is typically approximated via the so-called Lennard-Jones potential,^66^ or the Buckingham potential,^67^ the latter of which has an asymptotically correct exponential distance-dependence for the repulsive interaction energy component.^68^ For uncharged interaction partners, the vdW forces are dominated by London dispersion and the repulsive interactions,^69^ which, unlike the other components of vdW forces, are present between all atoms and all molecules.^70^ The additive nature of vdW forces means that they become particularly influential in larger systems, such as molecular crystals and biomolecules, where cumulative vdW energies significantly impact stability. The attractive vdW interaction energies between individual atoms usually constitute only a fraction of a kcal mol^–1^.^71^ However, due to their additive nature in molecular systems, vanishing directionality, and inability to saturate, combined interaction energies can readily surpass 40 kcal mol^–1^ for sufficiently large and specifically engineered systems.^2–4^

**Figure 1:**
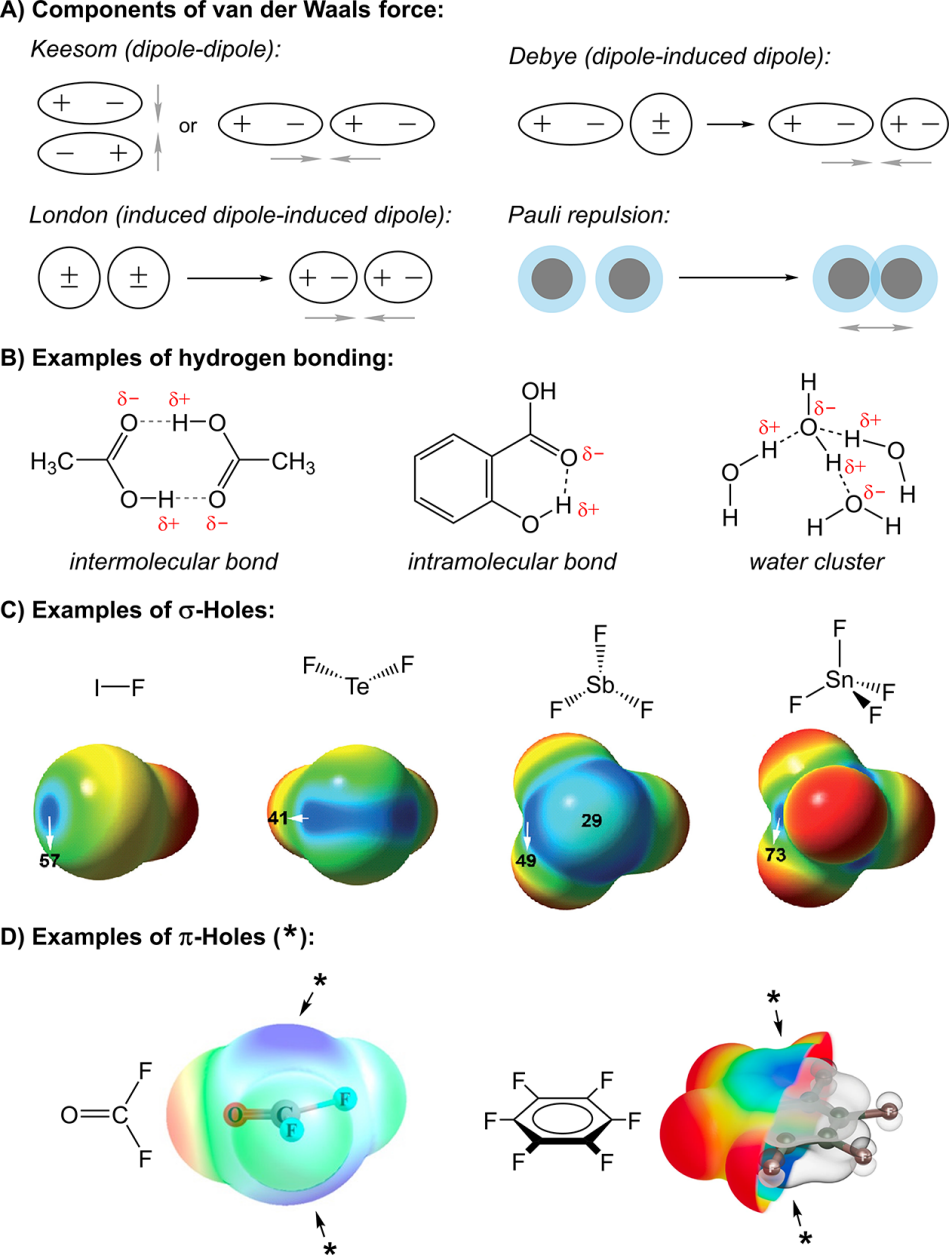
A) Components of the van der Waals force. B) Examples of hydrogen bonding. C, D) Molecular electrostatic potential surfaces, featuring σ- and π-holes, respectively. Red and blue areas represent negative and positive regions, respectively. σ-Holes are shown for halogen, chalcogen, pnictogen and tetrel fluorides, calculated at the MP2/aug-cc-pVTZ level of theory; potential values of the σ-holes are given in kJ mol^–1^.^83^ π-Holes (labeled with an asterisk) are given for carbonyl fluoride (M06–2X/aug-cc-pVDZ level of theory)^84^ and hexafluorobenzene.^85^ For the latter, the highest occupied molecular orbital (HOMO) is also shown. Subfigure C was adapted with permission from the literature.^83^ Copyright 2015 Wiley. Left-hand structure in Subfigure D was adapted with permission from the literature.^84^ Copyright 2021 American Chemical Society.

London dispersion is the only attractive component of the vdW force that is ubiquitous between atoms and molecules as it does not require permanent dipoles.^54^ It is best understood as an interaction between mutually induced temporary dipoles.^62^ Because it is a universal force, London dispersion significantly contributes to the stability of nonpolar substances and plays a critical role in molecular packing within the solid state. London dispersion interaction energies can be estimated based on the polarizabilities and the first ionization potentials of the interacting partners. At sufficiently large separations, the strength of London dispersion increases with the sixth power of the interatomic distance.^72^ In contrast, at close separations, this approximation no longer holds, and the interaction energy instead grows with the square of the interatomic distance.^73^ Whereas London dispersion interactions have long been appreciated in the gas phase to rationalize deviations from the ideal properties^74^ and in the solid state to understand the structure and stability,^75^ they have only been recently acknowledged as a non-negligible component of the interaction energy in solution.^76–78^

As briefly mentioned above, the repulsive interactions in the vdW forces originate from the Pauli exclusion principle, which is based on the indistinguishability of Fermions, such as electrons, and requires that no two Fermions may occupy the same state.^65^ Consequently, as two atoms approach one another, the Pauli exclusion principle forces the total wave function of the system to adjust. This causes a depletion of electron density in the region of overlap between the atomic wave functions, while the density shifts outward, closer to the areas surrounding each nucleus and away from the midpoint between them.^79^ As a result, nuclear charge screening is reduced, leading to increased electrostatic repulsion within the system.^79,80^ Accordingly, repulsive interaction energies can be modeled as a function of the squared overlap integral of the interacting atomic orbitals and the inverse of interatomic distance^68,79^ Whereas initially, repulsive interactions were modeled with the inverse twelfth power of the distance, this was merely a choice of algorithmic convenience for molecular modeling software rather than being grounded in physics.^66^ Based on quantum mechanics (QM), repulsive interactions are better modeled as an exponential function of the interatomic separation with a negative scaling factor in the exponent,^81^ which inspired the Buckingham potential.^67^ Repulsive interactions help define molecular boundaries and limit the degree of molecular overlap, providing structure and rigidity to condensed phases such as solids and liquids. In recent years, fueled by the increased usage of computational methods for energy decomposition analysis, the importance of repulsive interactions to understand both structure and reactivity of molecules has been increasingly appreciated.^82^

Hydrogen bonding (Figure 1B) occurs between a hydrogen atom that is bound to a more electronegative atom (the hydrogen bond donor) and another atom or group of atoms that is electron-rich (the hydrogen bond acceptor).^86^ Hydrogen bonding energies per bonded atomic pair span across 2 orders of magnitude, from 0.2 to 40 kcal mol^–1^,^87,88^ with a preferred interaction angle of 180°, although the exact value depends on the dominant interaction component,^89^ and the angles as low as 105° are also possible.^90^ Accordingly, this interaction tends to be significantly stronger than individual vdW forces between pairs of atoms (*vide supra*). Across the energy range, the exact nature of a hydrogen bond shifts, with varying contributions from electrostatic forces, dispersion forces, and charge transfer.^91,92^ Such a wide span of bonding strengths enables to contribute to both the subtle fine-tuning of molecular interactions in solution and the strong stabilization of macromolecular structures, for instance in protein folding and DNA base pairing.^93^ Consequently, the underlying mechanisms of hydrogen bonding have been a subject of a continuous theoretical and experimental debate^59,94–96^ since it was first proposed in the beginning of the 20th century.^97,98^ This debate is fueled by its relevance for a wide range of systems both in the gas phase and in condensed phases, including macromolecules and biochemistry.^99,100^

σ-Hole bonding encompasses several related types of NCIs, such as halogen, chalcogen, pnictogen, and tetrel bonding, each named after the group in the periodic table the interacting atom belongs to.^101^ It occurs when a positive electrostatic potential region, called a σ-hole (Figure 1C), forms on an atom that is covalently bound to an electronegative center with a bond that possesses σ-symmetry. This σ-hole normally forms on the extended bonding axis and can interact with the negative electrostatic potential of an electron-rich site (e.g., an anionic or neutral lone pair, or a π-bond), resulting in an attractive force.^101,102^ An atom with a positive interacting region is often termed a σ-hole donor, while an electron-rich site is called a σ-hole acceptor. Intuitively, this may contradict the electron-centered perspective, where the electron-rich site may be regarded as a donor instead. Halogen bonding is the most extensively studied NCI of this family and was also the first one to be discovered.^103,104^ It tends to have high directionality with a preferred angle of 180°,^105^ which makes the interaction geometries highly predictable, and provides high tunability by means of modifying both the halogen bond donor and acceptor moieties.^60^ While σ-hole bonding interactions tend to be unfavorable in the gas phase due to very significant entropic penalties,^106^ the enthalpic part of the interaction free energy can even exceed that of hydrogen bonding, varying from 1 to over 72 kcal mol^–1^ in extreme cases.^107,108^ In contrast, σ-hole bonding interactions are highly favorable in the condensed phase. Consequently, they are widely used in crystal engineering,^109^ and, recently, have also found application in organocatalysis.^110^ In terms of the underlying interaction mechanisms, σ-hole bonding is frequently compared to hydrogen bonding as they are both driven by the same combination of forces, namely electrostatics, dispersion, and charge transfer.^106,111,112^ Recent work even suggests that hydrogen bonding should also be considered a part of the σ-hole bonding family of interactions.^105,113^

In analogy to the definition of σ-hole bonding, π-hole bonding describes attractive interactions involving a π-hole (Figure 1D), i.e., the two regions of positive electrostatic potential that occur above and below the plane of the π-bound molecular motif. Similar to σ-holes, π-holes interact with the regions of negative electrostatic potential on the donor atoms, leading to net attraction.^84,114^ Typical examples of molecules with pronounced π-holes, and thus a tendency for π-hole bonding, include electron-deficient aromatic systems,^115^ and common interaction partners include anions and atoms with lone pairs.^115^ Therefore, before being classified as π-hole bonding, these interactions were initially described as anion-π interactions^116,117^ and lone pair-π interactions,^118^ respectively. Only the more recent literature focused on the similarities of π-hole bonding and σ-hole bonding, especially with respect to directionality and electrostatic potential structure.^115^ This paved the way for a common theoretical framework covering both these types of NCIs.^119^

While conceptually best described as a hole bonding interaction (*vide supra*), π-hole bonding formally belongs to the large group of π-interactions, a family of NCIs that relies on interactions involving π-electron density.^123^ Frequently, at least one of the interaction partners contains an aromatic system.^123^ Important subtypes of π-interactions are π-π stacking,^124^ ion-π interactions,^117,125^ and C–H···π interactions.^126^ π-π Stacking (Figure 2A) is governed by a combination of dispersion, Pauli exchange, and charge penetration – that is, electrostatic effects beyond the classical multipole expansion.^127^ The preferred interaction geometries of π-stacked dimers are largely determined by the so-called vdW potential that combines dispersion and exchange.^128^ The interaction energies for small closed-shell π-systems consisting of at most a handful of π-bonds typically fall within 0.5–2 kcal mol^–1^, reaching higher values only in exceptional cases.^129,130^ In contrast, cation−π interactions (Figure 2B) are governed by electrostatics, with significant contributions from both induction and dispersion, especially for sufficiently large organic cations.^131^ Cation−π interactions typically exhibit energies in the range of 2–4 kcal mol^–1^ in the condensed phase. For exceptional metal cation−π complexes, the energies can reach 6–13 kcal mol^–1^.^132,133^ Anion-π interactions (Figure 2B), a specific example of π-hole bonding (*vide supra*), are governed by electrostatics and induction, with dispersion forces playing a minor role.^134^ Energetically, these interactions rarely exceed 1 kcal mol^–1^ in solvated media.^135^ Finally, C–H···π bonds (Figure 2B) are mainly driven by dispersion, with the energy of an individual interaction being in the range of 1 kcal mol^–1^.^136^

**Figure 2:**
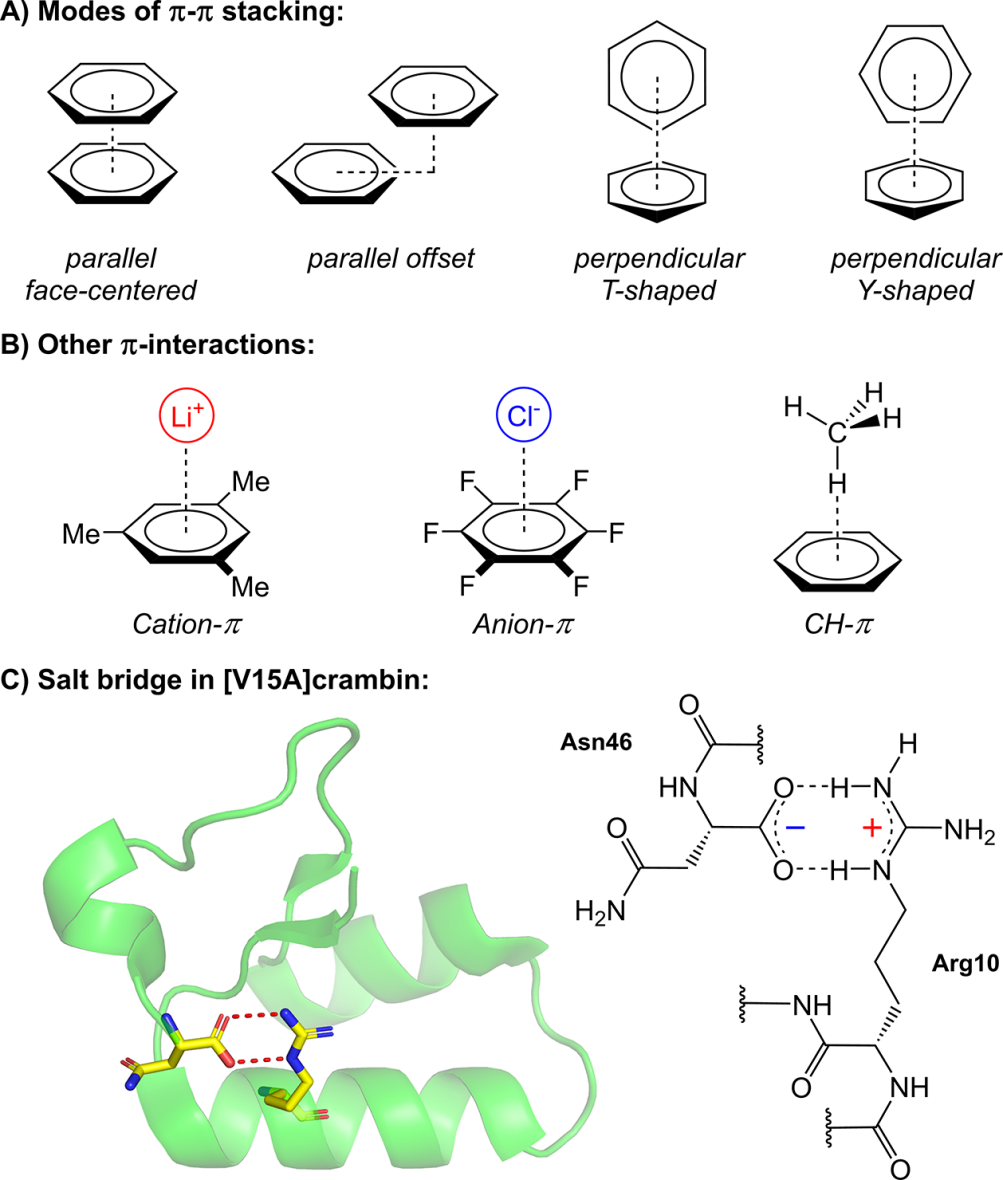
A, B) Various π-interactions, including different modes of π-π stacking;^120^ C) X-ray crystal structure of [V15A]­crambin (PDB accession code: 2FD7 ^121^) and the chemical structure of the salt bridge between the δ-guanidinium group of Arg10 and the α-carboxylate of the C-terminal Asn46. The protein structure is displayed in a ribbon representation, with the salt bridge highlighted in a stick representation.^122^

Ion pairing describes the association of two oppositely charged ions in solution and is governed by both the electrostatic attraction between the ions and the solvation of the individual ions.^137^ Ion pairs play a crucial role in mediating reactions in both organic and aqueous media, as they can significantly alter reaction rates and pathways depending on the solvent environment.^137^ Critical to the strength of ion pairing is the dielectric constant of the medium, which determines the attenuation factor of the Coulomb force.^138^ Together with hydrogen bonding, ion pairing gives rise to the so-called salt bridges (Figure 2C) – attractive interactions between at least two oppositely charged amino acid residues in proteins.^139^ There, the salt bridges contribute to the stability of the secondary and tertiary structure, influencing folding, conformational flexibility, and function, especially in enzymes and receptors.^140^ In contrast to ionic bonds in crystalline solids, these interactions are significantly weaker due to the screening of the electrostatic forces by the surrounding medium.^138^ The salt bridge strength in aqueous media is typically lower than 15 kcal mol^–1^, which is due to the large dielectric constant of water.^132^ Apart from that, salt bridges also strongly depend on the solution pH, which determines the protonation state, and thus the charge of the interacting residues,^139^ and on the ionic strength of the surrounding medium, as described by the Debye–Hückel theory.^141^

### 2.2 Identifying the Nature of NCIs

A widely used approach for identifying the nature of specific NCIs is the application of Symmetry-Adapted Perturbation Theory (SAPT). This theoretical framework decomposes the total interaction energy between interacting molecular subsystems into physically meaningful components, providing insight into the forces governing the corresponding molecular interactions. SAPT was first introduced by Jeziorski and Szalewicz^142^ and, as its name already implies, is based on perturbation theory. Specifically, the interaction energy between two monomers is treated as a perturbation to their individual isolated wave functions. Unlike the traditional supermolecular approach for computing interaction energies between monomers, which computes interaction energies as the difference between the total energies of dimer and monomers, SAPT avoids the need for basis set superposition corrections and provides a rigorous breakdown of the interaction into fundamental physical components. The total SAPT interaction energy between the interacting monomers is divided into electrostatics, exchange-repulsion, induction and dispersion.

SAPT is one of many energy decomposition analysis (EDA) methods^143^ used to dissect interaction energies. Other commonly used approaches include: Kitaura-Morokuma (KM) EDA,^144^ Absolutely Localized Molecular Orbitals (ALMO) EDA,^145^ Natural Energy Decomposition Analysis (NEDA),^146,147^ Ziegler-Rauk EDA,^148^ and Local Energy Decomposition (LED).^149^ While all these methods provide valuable insights, they often rely on arbitrary choices, such as the definition of reference states and orbital localization schemes or introduce varying degrees of basis set superposition dependencies. Among these, SAPT stands out as it is directly derived from perturbation theory rather than from orbital partitioning. Additionally, it is based on comparably few empirical assumptions, most importantly the choice of the unperturbed reference state and the grouping of the interaction energy terms. This provides SAPT with a robust theoretical foundation. The key assumption in its derivation is that the interaction is assumed to be sufficiently weak, making SAPT ideal for the study of noncovalent interactions, but less suitable for covalent bonding or other strong interactions.

Despite its appeal in terms of theoretical foundation, SAPT can be computationally expensive, especially at higher orders of perturbation theory. Its accuracy depends on both the truncation level of the perturbation expansion and the chosen basis set, often requiring relatively large basis sets for high accuracy.^150^ To strike a balance between accuracy and computational efficiency for larger and more complex chemical systems, SAPT­(DFT) was developed.^151,152^ This method relies on frequency-dependent monomer density susceptibilities based on time-dependent density-functional theory for dispersion, Kohn–Sham orbitals and their energies for exchange, electrostatic interaction energies based on monomer Kohn–Sham determinants, and Kohn–Sham static response theory for induction. It was found to provide highly accurate interaction energy components, on par with the best SAPT methods relying on wave function approaches but offering significantly reduced computational scaling with system size. This makes SAPT­(DFT) particularly appealing for generating very large high-quality reference data sets for downstream ML applications.

## 3 Overview of Machine Learning Approaches

ML encompasses a wide array of algorithms and computational frameworks that enable to identify patterns and make predictions from data automatically – a process that does not require explicit programming of task-specific rules.^153^ Broadly, the field can be divided into three main branches: supervised learning, unsupervised learning, and reinforcement learning.^154^ Each of these branches represents a unique approach to extracting insights from data, with specific methods suited to different kinds of problems and objectives.

### 3.1 General Classification

Supervised learning operates by training models on labeled data sets, where each instance has a known output, or a so-called label.^155^ This process is essential for prediction tasks, where the goal is to map input data to a corresponding output by minimizing the error between predictions and actual values.^156^ Supervised learning tasks can include classification, where the output is categorical (e.g., identifying the functional group of a molecule), or regression, where the output is continuous (e.g., predicting p*K*_a_ value). For example, in an image classification problem, a labeled data set might contain images tagged with object labels, such as “flask” or ”liquid.” By training on this data, a supervised model learns to generalize, allowing it to classify new, unseen images accurately.

Various ML architectures for supervised learning have been developed to address the specific needs of different data types and problem objectives. Some essential supervised algorithms include multiple linear regression (MLR), a fundamental statistical technique that models the relationship between multiple independent variables and a dependent variable.^157^ MLR serves as a baseline model for understanding and predicting linear relationships in data, making it a cornerstone for more advanced regression techniques. However, in high-dimensional data sets, MLR is prone to overfitting, where the model captures noise rather than the true underlying pattern. To mitigate overfitting and improve generalization, ridge regression, an extension of MLR, applies L2 regularization by penalizing large coefficients.^158^ Ridge regression helps prevent models from becoming too complex (a common issue in high-dimensional data sets) and ensures better stability. It provides a useful baseline for predicting relationships between variables while controlling variance. Kernel ridge regression expands the capability of ridge regression by allowing to model nonlinear data sets by means of the kernel trick, which allows projecting nonlinear data into a higher-dimensional space, where the linear separation is feasible.^159^ Similarly, support vector machines (SVMs) aim to find an optimal hyperplane that separates data points from different classes with a maximum margin.^160^ The SVM algorithm is highly effective in classification tasks, especially when combined with kernel functions that enable it to capture nonlinear relationships. Ensemble models, such as random forests (RFs) and gradient boosting algorithms, take a different approach by combining multiple learners to improve predictive power and robustness. RFs, for example, construct multiple decision trees from random subsets of the data and average their predictions, reducing overfitting and increasing stability. XGBoost (XGB),^161^ a type of gradient boosting, incrementally builds trees to correct errors made by previous trees, resulting in a highly efficient and accurate model that often excels in predictive benchmarks.

Unsupervised learning, unlike supervised learning, works with unlabeled data to find inherent patterns, clusters, or structures without predefined labels. It is commonly applied in tasks like clustering, dimensionality reduction, and generative modeling. For instance, clustering algorithms can group data points based on similarity, such as organizing molecules by reactivity or clustering visually similar images. Dimensionality reduction techniques, like principal component analysis (PCA)^162^ or t-distributed stochastic neighbor embedding (t-SNE),^163^ transform high-dimensional data into a lower-dimensional representation, making it easier to visualize and interpret complex patterns in data sets. Generative models, a specialized class within unsupervised learning, learn the data distribution for generating new samples. For instance, generative adversarial networks (GANs)^164^ and variational autoencoders (VAEs)^165^ are unsupervised models that create new data points that are, ideally, statistically indistinguishable from the original data. They are used widely in areas from molecule generation and image synthesis to anomaly detection. Another noteworthy technique, contrastive learning, aims to create useful embeddings by maximizing agreement between representations of similar data points while minimizing similarity for dissimilar ones, effectively enhancing the quality of representations for clustering or downstream supervised tasks.^166^

Reinforcement learning (RL) is a distinct branch of ML that involves training an agent to make decisions through interaction with an environment.^167^ Unlike supervised and unsupervised learning, RL models learn by receiving feedback in the form of rewards or penalties based on their actions. This approach is well-suited to tasks requiring sequential decision-making, such as robotics, game playing, autonomous driving, or reaction optimization. In RL, the actions of an agent are guided by a policy that is optimized to maximize cumulative rewards over time.^168^ The agent learns to balance exploration (trying new actions) with exploitation (choosing actions known to yield good results). Core RL frameworks, like Q-learning^169^ and policy gradient methods,^170^ provide foundational algorithms for training agents in both simulated and real environments, allowing for increasingly sophisticated control strategies as more data is gathered.

The workflow for training supervised ML models (Figure 3) begins with data acquisition and preprocessing, including feature generation.^171^ Features, such as pixel intensities in images or vectors representing functional groups and structural moieties in molecules, describe each data point, while labels indicate the target values for supervised tasks.^172^ Typically, data is divided into training, validation, and holdout sets, each serving a unique role in building and assessing the performance of a model. Careful selection and engineering of relevant features are crucial, as they directly influence the patterns the model can learn and the accuracy of its predictions. Accordingly, feature engineering is an intrinsic part of the overall model architecture. Once preprocessed, the training data is used to optimize a loss function that quantifies prediction errors. The aim is to minimize this error through an iterative learning process. This process is often implemented via variants of the stochastic gradient descent algorithm, which optimizes the model parameters.^173,174^

**Figure 3:**
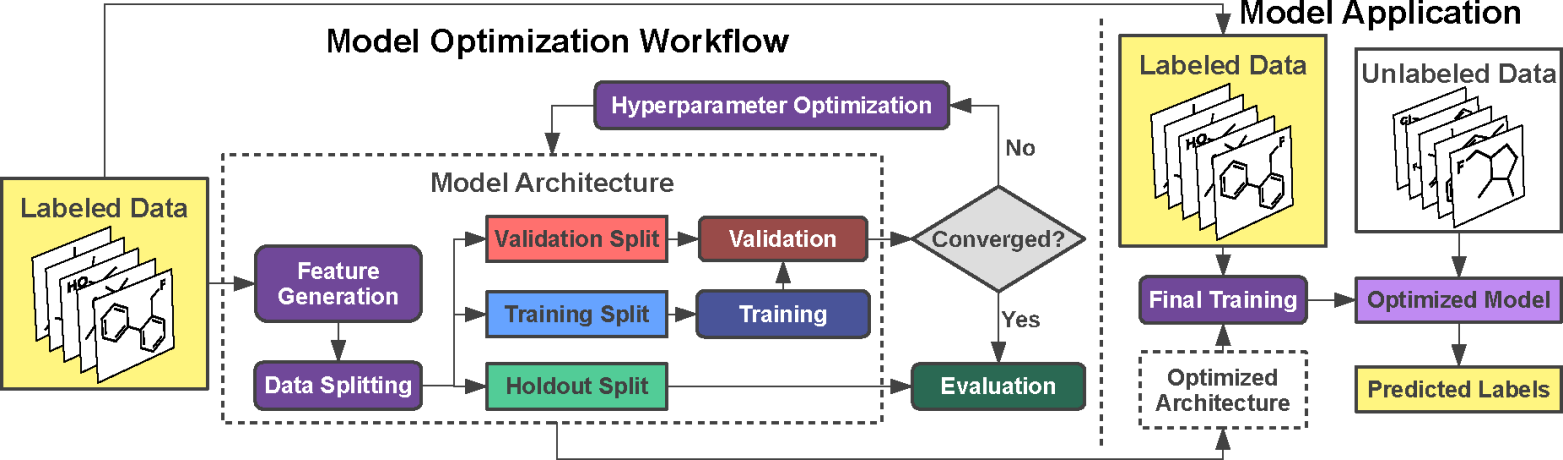
Model optimization workflow and model application in supervised learning.

Alongside these learning algorithms, hyperparameter optimization is essential for achieving optimal model performance. Hyperparameters, unlike parameters learned during training (such as neural network weights), control the learning process and must be defined before training, as analytical gradients cannot be readily computed for them.^175^ Examples of critical hyperparameters include the learning rate, which affects the convergence speed in gradient descent optimization; regularization strength, which prevents overfitting by penalizing complex models; and model depth, which impacts complexity by determining the number of layers or trees. Properly tuning these values is essential to balance model accuracy with generalization capability and avoid overfitting. Hyperparameter optimization techniques such as grid search and random search have traditionally been used to explore a range of parameter values.^176^ Grid search systematically evaluates combinations within a specified range, while random search samples hyperparameter values randomly, often achieving competitive results with fewer evaluations.^176^ More advanced methods, such as Bayesian optimization, build probabilistic models to predict the performance of different hyperparameter configurations, guiding the selection of promising candidates and often reducing the number of evaluations required.^177^ Automated approaches like gradient-based tuning and evolutionary algorithms enable efficient exploration of complex hyperparameter spaces, which is particularly useful for deep learning models.^178,179^ Effective hyperparameter optimization maximizes model performance and robustness, helping to prevent overfitting and ensuring reliable generalization to new data.

### 3.2 Deep Learning

Deep learning, a specialized subset of ML, has gained remarkable traction in recent years due to its ability to automatically extract complex patterns from large data sets. Unlike traditional ML models, which often require manual feature selection, deep learning models leverage multilayered neural networks to identify intricate relationships with minimal human intervention. These models have proven particularly useful in deciphering the nuanced, nonlinear interactions characteristic of noncovalent interactions. In this section, we will explore key deep learning architectures, including feedforward neural networks (FNNs), convolutional neural networks (CNNs), and other relevant approaches, highlighting their applications and advantages in modeling and predicting NCIs.

Feedforward Neural Networks (FNNs)^180^ are the foundational neural network model architecture, structured in layers, where each neuron connects only to the neurons in the next layer (Figure 4A). Information flows linearly from input to output, which simplifies training and interpretation. This architecture excels in settings where data is tabular or lacks explicit spatial or temporal structure, making it widely applicable for predictive modeling tasks. FNNs are ideal for relatively straightforward tasks like predicting physicochemical properties (e.g., solubility, boiling point) from molecular descriptors or other structured data sets. They consist of an input layer, one or more hidden layers, and an output layer. Each hidden layer performs a combination of a linear transformation via learned weights and biases, and a nonlinear transformation via a so-called activation function, enabling the network to capture nonlinear relationships in data. However, FNNs are limited in their ability to model complex, high-dimensional structures with important spatial or temporal dependencies. This is where more specialized architectures come into play, which will be discussed in the following paragraphs.

**Figure 4:**
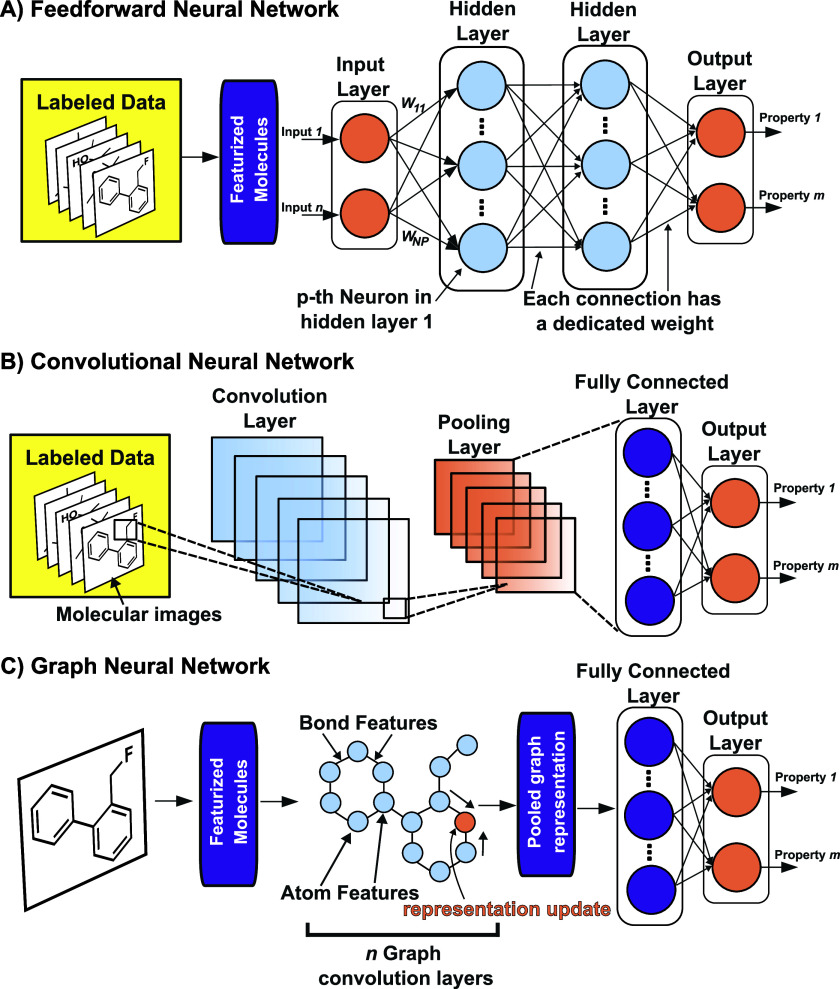
Neural network architectures used for molecular property prediction. A) Feedforward neural network: Molecular features (input) are processed through fully connected layers to predict molecular properties (output). B) Convolutional neural network: Molecular images are processed through convolution and pooling layers before reaching the fully connected layer providing the output. C) Graph neural network: Encodes molecules as graphs with atom and bond features. Graph layers update feature representations, and the pooled graph representation is fed to the fully connected layer, providing the output.

Convolutional Neural Networks (CNNs),^181^ originally developed for computer vision,^182^ have become indispensable in applications that involve spatial or structured data. A CNN uses convolutional layers (Figure 4B) to apply filters across local patches of data, allowing it to capture spatial patterns in an efficient and hierarchical manner.^183^ In the context of molecular data, CNNs can interpret chemical structures, such as drawings or even 3D grids of electron density.^184^ For instance, when applied to protein structures, CNNs can detect recurring patterns (like binding pockets or surface charges) critical for understanding molecular interactions.^185^ Each convolutional layer in a CNN is usually followed by a pooling layer, which reduces dimensionality and helps the network focus on the most salient features, improving efficiency and reducing overfitting. This type of architecture is well-suited for analyzing molecular structures represented in pixel-based or voxel-based formats, where spatial relationships are vital to understanding the corresponding molecules.^186^

Graph Neural Networks (GNNs)^187^ provide a specialized approach for analyzing data that is naturally structured as a graph, such as molecules, protein–ligand complexes, and molecular assemblies. In GNNs, molecular structures are typically represented as graphs, where nodes can correspond to atoms, functional groups, or even entire molecular fragments, while edges capture their relationships, such as chemical bonds or spatial proximity (Figure 4C). GNNs process information by propagating node features (e.g., atomic types, charges) through the graph, aggregating information from neighboring nodes and edges. This enables the model to learn meaningful representations that reflect the spatial arrangement and interactions of molecular components, which is crucial for predicting molecular properties, binding affinities, and reaction outcomes. Several GNN variants, such as Message Passing Neural Networks (MPNNs)^188^ and Graph Convolutional Neural Networks (GCNs),^189^ have been developed to optimize information flow across nodes, allowing the model to capture both local interactions and long-range dependencies. Recently, the connection between these two frameworks has been highlighted, interpreting GCNs as a special class of MPNNs.^190^ In recent years, GNNs have become the de facto standard for molecular property prediction.^191^

These architectures provide a rich set of tools to tackle challenges across diverse scientific fields, with each offering distinct advantages for specific types of problems and data. FNNs handle straightforward predictive tasks well, CNNs excel in identifying spatial patterns within images or 3D grids, and GNNs capture connectivity information, making them powerful for molecular applications. Apart from these approaches, transformer architectures mark one of the most significant advancements in ML, especially for sequence-based data like language or time series.^192^ Unlike recurrent neural networks,^193^ which process data sequentially, transformers enable each element in a sequence to interact directly with every other element. This self-attention mechanism captures long-range dependencies and contextual relationships, making transformers indispensable in tasks like natural language processing, where contextual information is crucial. Their adaptability has since led to applications across various research fields where sequence and relational data are central, with many application examples in chemistry.

These ML approaches provide a robust framework for advancing the study of NCIs by enabling nuanced analysis and prediction of complex molecular behaviors. For instance, ensemble methods and neural networks can capture the subtle energy contributions from multiple interaction types, which is essential for predicting the stability and behavior of molecular assemblies. GNNs and transformer-based models are particularly well-suited for capturing spatial and structural dependencies, which are critical for modeling the intricate and diverse types of NCIs that drive molecular organization. Furthermore, the generative capabilities of models like VAEs open avenues for designing molecular structures with targeted interaction profiles, aiding in applications like drug discovery and materials design.

In this review, we will explore how ML methods (and deep learning in particular) are applied to modeling, predicting, and designing NCIs, illustrating their transformative role in uncovering and utilizing the underlying principles that govern molecular interactions.

## 4 Data Sets for Noncovalent Interactions

Every ML project stands and falls with its training data. The amount and quality of the available data directly determines what predictions can and cannot be expected from an ML model. In contrast to the overwhelming diversity of the chemical compound^194^ and reaction space,^195^ systematically collected data that is immediately actionable for ML deployment is sparse and scattered.^196,197^ Therefore, our aim in this section is to provide a thorough overview of existing data sets relevant to the characterization of NCIs, including both structural and property-based information. We hope that this will inspire further research into applying ML models for the understanding, prediction, and, ultimately, design of NCIs.

High-accuracy NCI energies are essential benchmarks for potential energy surfaces and for refining approximate computational methods. Unlike covalent bonds, NCIs are subtle and often nonlocal, with only little impact on electronic structure, demanding precise experimental and theoretical approaches. Experimentally, isolating these effects requires highly sensitive techniques as NCIs are one to two orders of magnitude weaker than covalent forces.^198–201^ Theoretically, electrostatically driven NCIs, such as hydrogen bonding, are generally described accurately, at least qualitatively, at the Hartree–Fock (HF) level.^202^ However, Hartree–Fock theory, semilocal density functional theory (DFT)^203–205^ or even highly parametrized exchange-correlation functionals^206^ fail to capture dispersion in a correct manner already at interaction distances of around 3 Å until the asymptotic limit. This results in a substantial underestimation of the stability of dispersion-dominated complexes. Stemming from the long-range electron correlation, dispersion can be captured by post-Hartree–Fock methods, which do not consider separate electrons as moving independently. While the second-order Mo̷ller–Plesset perturbation theory (MP2) is one of the simplest methods that correctly does so, it tends to overestimate dispersion significantly, especially in π-systems.^207^ True quantitative accuracy is achieved only through advanced *ab initio* methods, ideally extrapolated to the complete basis set limit (CBS).

The long-standing ”gold standard” for calculating NCIs has been the coupled-cluster method with single, double, and perturbative triple excitations, CCSD­(T),^208^ converged to the CBS limit, a level of theory thoroughly detailed in a large number of research papers and reviews.^209–211^ For even greater accuracy, the so-called ”platinum standard”^210^ involves modified or post-CCSD­(T) methods with additional corrections for contributions such as relativistic effects, core correlations, and quantum electrodynamic effects. However, this approach is typically only feasible for very small systems, often limited to merely a few nuclei and electrons. Even the ”gold standard” itself, due to its steep computational scaling with the seventh power of the system size, is mostly applicable to small- and midsized organic molecules (i.e., less than ≈30 atoms). By now, the field of the corresponding NCI benchmark data sets is near its saturation point, as thoroughly reviewed by several authors.^209–215^ These data sets are widely used in computational chemistry and are often included in composite benchmarks, such as BEGDB^211^ and GMTKN55.^212^

The inclusion of larger systems is particularly important because they capture the collective nature of NCIs. In the context of the condensed phase, whether amorphous or periodic, the advancement of relevant computational methods is hindered by a lack of reliable data sets and the uncertainty regarding the applicability limits of current approaches.^216,217^ This creates a vicious cycle that, however, is beginning to be disrupted with the availability of dependable experimental benchmarks.

Overall, substantial advancements are still needed for data sets that include experimental data, large systems, heavy atoms, open-shell systems, and excited states.^209,211^ Computationally, modeling these effects is becoming more affordable with increasing computational resources and both the advent and adoption of new approximations to account for London dispersion in an efficient manner.^218^ Important developments include the dispersion corrections developed in the Grimme group,^219–223^ composite DFT methods such as r^2^SCAN-3c,^224^ local coupled cluster methods like DLPNO–CCSD­(T),^225^ the many-body dispersion (MBD) correction,^226,227^ and vdW density functionals such as VV10.^228,229^ A more recent development is the universal atom-pairwise interatomic van der Waals potential based on the quantum Drude oscillator model.^230,231^

In the following subsections, we review several key open-access data sets, or the most recent versions thereof, that are relevant for modeling large molecular, solid-state systems, and those involving heavy elements (cf. Tables 1–3). We also discuss the most recent generation of information-rich NCI benchmarks (Table 4), followed by the few available data sets for NCIs in the excited state. We include both experimental and computational data, and focus on the nature of the interacting molecules. It is worth noting that the majority of these data sets were initially created to aid with the development of computational methods rather than for training ML models. However, they provide valuable information that can immediately be used for training ML models without substantial data preprocessing.

**Table 1:** Data Sets Covering Noncovalent Interactions in Large Molecular Systems

Data Set	Description	Structure Source	Energy Source	Size
L7 ^241^	Oligomeric closed-shell organic complexes, mainly dispersion. **S Tier**.	DFT or X-ray diffraction	QCISD(T)/CBS	7 systems (48–112 atoms)
S12L^242^	Host–guest closed-shell organic complexes, various interactions. **A Tier**.	TPSS-D3/def2-TZVP	Experiment	12 systems (86–177 atoms)
S30L^243^	Host–guest organic complexes, flexible host molecules, anionic, neutral, and cationic structures, various interactions. **A Tier**.	TPSS-D3/def2-TZVP	Experiment	30 systems (up to 200 atoms)
HS13L^244^	Host–guest organic complexes, with heavy main group elements (Zn, As, Se, Te, Br, I), anionic, neutral, and cationic structures, various interactions. **A Tier**.	r^2^SCAN-3c//COSMO-RS	Experiment (back-corrected)	13 systems (37–266 atoms)
UpU46 ^245^	Conformers of uracil dinucleotides. **A Tier**.	TPSS-D3/def2-TZVP//COSMO	DLPNO–CCSD(T)/CBS	46 conformers (59 atoms each)
IONPI19 ^246^	Ion−π complexes, cationic and anionic, 10 elements (C, H, N, O, Li, Na, K, S, F, Cl). **A Tier**.	PBEh-3c or RI-MP2/aug-cc-pVTZ^247,248^	CCSD(T)/CBS, DLPNO–CCSD(T)/CBS, or W1–F12	19 systems (up to 133 atoms)
AQM^249^	Many-body dispersion in solvated organic molecules. **B Tier**.	DFTB3-MBD//GBSA	PBE0-MDB//MPB	1,653 systems (2–92 atoms)
Pollice et al. 1 ^250^	Dispersion in homo- and heterodimers of alkanes and perfluoroalkanes. **A Tier**.	DSD-PBEP86/def2-TZVP(spd)	CCSD(T)-F12/cc-pVDZ-F12 or DLPNO–CCSD(T)/CBS	18 systems (10–40 atoms)
Vik et al.^251^	Influence of n– π* interactions on transition state barriers of molecular rotors. **A Tier**.	B3LYP-D3(0)/6–311G*	Experiment and B3LYP-D3(0)/6–311G*	16 systems (31–50 atoms)
Lin et al.^252^	Influence of pnictogen interactions on transition state barriers of molecular rotors. **A Tier**.	B3LYP-D3(0)/6–311G*	Experiment and B3LYP-D3(0)/6–311G*	20 systems (33–59 atoms)
Yang et al.^253^	Influence of π–stacking on binding in hydrogen-bonded heterodimers. **A Tier**.	B3LYP/6–31G* and M06–2*X*/6–31G*	Experiment and SAPT0/aug-cc-pVDZ’	20 systems (79–115 atoms)
Pollice et al. 2 ^76,254^	Dispersion in proton-bound homodimers of 2,6-disubstituted pyridines. **A Tier**.	B97-D3/pc-2-sp(d)	Association free energies from experiment and computed via DLPNO–CCSD(T)/CBS, with SMD or COSMO-RS solvation	38 systems in the gas phase and in solution (23–171 atoms)
Wilming et al.^255^	Dispersion in conformer equilibria of 2,2’-disubstituted 9,9’-bifluorenylidene molecular balances. **A Tier**.	PBEh-3c	Experiment, revDSD-PBEP86-D4/def2-QZVPP//SMD, and DLPNO–CCSD(T)/def2-TZVP//SMD	13 systems (48–91 atoms)
Wang et al.^256^	Data set for binding free energies of protein–ligand complexes. **S Tier**.	Experimental (XRD) and simulated (OPLS)	FEP simulations (OPLS) and experimental binding affinities	529 systems
Schindler et al.^257^	Data set for binding free energies of protein–ligand complexes. **S Tier**.	Experiment (XRD) and simulated (OPLS)	FEP simulations (OPLS) and experimental binding affinities	296 systems

### 4.1 Data Set Accuracy

The utility of any data set in the realm of ML stands and falls with its underlying data quality. However, depending on the type of application, the required data accuracy can greatly differ. For supervised learning with NCI interaction energies as labels, high accuracy is critical. In contrast, when using these interaction energies as features, precision is more important than accuracy as models can rescale the input data via their trained parameters. Similarly, for designing NCIs, high training data accuracy is not necessarily required as the corresponding generative models can leverage general trends rather than absolute interaction energies. Accordingly, the data sets we included in the following sections have distinct levels of accuracy. Consequently, their ideal target applications are distinct as well.

When accurate interaction energies or structures of NCI complexes are to be predicted via ML models based on reference data, we recommend to rely on the existing accuracy evaluation metrics that are commonly used for benchmarking computational chemistry methods as a guide for data set selection. Typically, these accuracy metrics are based on errors in interaction energies or energy differences relative to either experiments or gold-standard electronic structure methods such as CCSD­(T)/CBS for NCIs.^232^ For geometries, the error metrics are either based on root-mean-square deviations of predicted bond distances and bond angles, relative to CCSD­(T)/CBS^233^ or experimental structures,^234^ or based on errors in predicted rotational constants relative to experimental results.^235,236^

Good practices for the generation of benchmark databases for electronic structure methods have been reviewed and summarized recently.^237^ We recommend these guidelines to be followed for any data set that is to be used for training ML models with the goal of predicting structures of NCI complexes or the corresponding interaction energies accurately. In contrast, for data sets that are to be used for ML models predicting the presence or absence of specific NCIs, for advanced featurization strategies, or for designing NCIs, precision is more important than accuracy. That is, a consistent description of the individual interactions in the data set is sufficient rather than providing absolute accuracy with regards to the corresponding interaction strength.

Based on these considerations, we decided to classify the data sets to be discussed in the following sections into three tiers, indicating their accuracy level and alluding to their ideal application domain. These accuracy classifiers are mentioned in the summarizing tables of each section. The **S Tier** indicates data sets of the highest quality that provide both benchmark quality structures and energies. The **A Tier** indicates data sets of benchmark quality for either energies or geometries, but not both. The **B Tier** indicates data sets with high precision for NCIs but insufficient accuracy in both energies and geometries to be considered benchmark quality for these properties. In general, we based this classification on the results of comprehensive benchmark studies of computational approaches for NCIs.^212,233,235,238^

### 4.2 Large Molecular Systems

Systematic benchmark data sets for NCIs that include molecules with more than ≈30 atoms are rare due to the associated prohibitive computational demand. However, advances in computing power, algorithm efficiency, and accurate electronic structure methods with reduced computational scaling have enabled accurate *ab initio* modeling of large molecular systems in recent years.^239^ Additionally, systematic experimental benchmarks based on the so-called molecular balances and binding energies of intermolecular complexes, such as host–guest interaction energies, have complemented computational approaches and served as a valuable addition to test the accuracy of state-of-the-art electronic structure methods.^240^ Below, we discuss currently available data sets that cover large molecular systems and are based on computational data, experimental data, or both. As a criterion for inclusion, we require a minimum of 7 different molecules to be present, as well as both structural information and data characterizing the corresponding interaction strength.

#### 4.2.1 L7 Data Set^241^

This is a computational set of 7 dimeric or oligomeric closed-shell organic complexes with bonding mainly dominated by dispersion, 48–112 atoms each. Selected complexes represent key dispersion-dominated motifs in biological chemistry. Molecular geometries were either optimized without constraints at the DFT level of theory or taken from X-ray crystallographic data published elsewhere. For interaction energies within the complexes, QCISD­(T)/CBS was chosen as a reference method because of higher computational efficiency and yet comparable precision to that of CCSD­(T)/CBS. Later, the interaction energies were recalculated at the DLPNO–CCSD­(T)/CBS level.^258^

#### 4.2.2 S12L Data Set^242^

This is an experimental set of 12 host–guest closed-shell organic complexes, 24 to several hundreds of atoms each, formed from 6 host molecules and 2 guests. Unlike the L7 set, where six out of seven complexes are primarily dominated by dispersion forces, the S12L complexes exhibit a broader range of NCIs, including hydrogen bonding, dispersion, π-stacking, and cation-dipole interactions. Molecular geometries were optimized at the TPSS-D3/def2-TZVP level of theory, and reference association free energies were taken from experiment.

#### 4.2.3 S30L Data Set^243^

This is a broader version of the experimental S12L set: 11 out of 12 original S12L host–guest organic complexes were complemented with others featuring more flexible hosts such as crown ethers and cyclodextrins with adaptable alkyl side chains. In total, it includes 30 complexes with up to 200 atoms and charges ranging from – 1 up to +4. The data set features key supramolecular interactions, including hydrogen bonding, halogen bonding, π–π stacking, dispersion, CH-π, and cation–dipole interactions. Molecular geometries were either taken from the S12L set or optimized at the TPSS/def2-TZVP level of theory. Reference association free energies were taken from experiment.

#### 4.2.4 HS13L Data Set^244^

This is a primarily experimental set of 13 host–guest complexes that exhibit a variety of NCIs involving heavier main group elements (Zn, As, Se, Te, Br, I) and relatively diverse charges, ranging from – 2 to +4. All complexes are within 37–266 atoms and display considerable HOMO–LUMO gaps. The authors performed a detailed conformational analysis and selected the lowest-lying conformers for each complex to be further refined at the r^2^SCAN-3c level combined with continuum solvation in the solvents also used for the experiments. Reference association free energies were taken from experiments in solution and complemented with simulated electronic energies at the DLPNO–CCSD­(T)/CBS level of theory, or the PWPB95-D4/def2-QZVP level when the former was infeasible.

#### 4.2.5 UpU46 Data Set^245^

This is a computational data set of 46 uracil dinucleotides, 59 atoms each, that represent all known RNA backbone conformation families. Accordingly, this benchmark collects all important structure-energy points on the dinucleotide potential energy surface. The molecular geometries were generated after constrained optimization with TPSS-D3/def2-TZVP//COSMO, and the reference energies were derived by means of DLNPO–CCSD­(T)/CBS. While the authors of the UpU46 data set do not explicitly use this data to study NCIs, every structure has the same bond composition but features varied degrees of π-stacking and dispersion, which may be of use for both parametrizing and benchmarking other computational methods and for ML applications.

#### 4.2.6 IONPI19 Data Set^246^

This is a computational data set of 19 intramolecular ion−π complexes that comprises 10 elements (C, H, N, O, Li, Na, K, S, F, Cl) and is split into ten cationic and nine anionic systems. The average system size in the data set is 32 atoms per molecule with 14 smaller systems (i.e., ≤ 30 atoms) and 5 larger ones (i.e., > 30 atoms, up to 133 atoms). Molecular geometries were either optimized at the PBEh-3c level of theory or taken from other sources that utilized RI-MP2/aug-cc-pVTZ for geometry optimizations.^247,248^ Reference interaction energies were calculated using one of CCSD­(T)/CBS, DLPNO–CCSD­(T1)/CBS, or W1–F12.

#### 4.2.7 Aquamarine (AQM) Data Set^249^

This is a computational data set designed for aiding with the modeling of large solvated molecules relevant to pharmaceutical or biological research, comprising the total of 8 elements (C, N, O, H, Cl, S, P, and F). AQM captures interactions with solvent molecules and includes collective dispersion effects by means of many-body dispersion (MDB).^226^ It comprises 1,653 molecules, ranging within 2–92 atoms, with the average being 50.9 atoms, and features 59,783 conformers spanning both low and high energy. After conformational search using CREST,^259^ the final geometries were optimized at the DFTB3-MBD level of theory, both in the gas phase and in implicit water via the GBSA implicit solvent model.^260^ The final energies and MBD corrections, together with over 40 local and global properties were computed at the PBE0+MDB level of theory, without and with implicit water via the modified Poisson–Boltzmann (MPB) solvation model.^261^

#### 4.2.8 Pollice et al. 1 ^250^

This is a computational data set for the binding and interaction energies of the dispersion-dominated intermolecular complexes of linear alkanes and perfluoroalkanes with up to 6 carbon atoms in the interacting molecules. The data set was used as a foundation to study the origin of the immiscibility of alkanes and perfluoroalkanes, the so-called fluorophobic effect.^262^ The data sets consists of 18 molecular systems with 10–40 atoms. Geometries were optimized at the DSD-PBEP86/def2-TZVP­(spd) level of theory and both binding and interaction energies were computed at the CCSD­(T)-F12/cc-pVDZ-F12 or the DLPNO–CCSD­(T)/CBS levels. These results were used to perform a comprehensive benchmark of both DFT and wave function-based methods (WFT) methods that account for dispersion interactions, demonstrating that many methods lead to considerable errors for intermolecular complexes of perfluoroalkanes. The majority of the tested methods underestimated the binding energies significantly. SAPT methods consistently confirmed that the interaction energies are dominated by exchange and dispersion, with attractive dispersion overriding repulsive exchange. This work demonstrated that fluorine in organic compounds contributes strongly to dispersion interactions but leads to unfavorable interaction geometries in intermolecular complexes involving perfluoroalkanes, which ultimately causes phase separation.

#### 4.2.9 Vik et al.^251^

This is a combined experimental and computational data set that was created for studying the influence of intramolecular *n* – π* interactions on the rotational barrier of *N*-aryl-substituted imides. Intramolecular *n* – π* interactions are of particular interest because of their role in determining conformations of peptides and proteins.^263^ The data set encompasses 16 molecules ranging from 31–50 atoms. The rotational barriers were determined via ^1^H NMR exchange spectroscopy (EXSY) in the slow exchange regime (−60–130 °C).^264^ The experimental results were complemented with simulations of the rotation barriers at the B3LYP-D3(0)/6–311G* level of theory. The authors isolated the electronic contribution of the interaction via the so-called steric B-values, which are based on rotational barriers of biphenyl compounds.^265^ The authors found significant energetic stabilization of the bond rotation transition states by up to 9.7 kcal mol^–1^.

#### 4.2.10 Lin et al.^252^

The same as the above class of compounds and methodology were used to quantify the impact of intramolecular pnictogen interactions, where pnictogen is a nitrogen atom, on the rotational barriers of *N*-aryl-substituted imides. These interactions belong to the large family of σ-hole bonding (*vide supra*). The data set encompasses 20 molecules ranging within 33–59 atoms. The authors found significant energetic stabilization of the bond rotation transition states by up to 8.4 kcal mol^–1^, and correlation with molecular electrostatic potential values at the interacting nitrogen atoms suggested electrostatics to be the dominant interaction energy component.

#### 4.2.11 Yang et al.^253^

This is a combined experimental and computational data set that is aimed at quantifying the contributions of π-π stacking on the heteromolecular association equilibrium of substituted naphthyridines and ureas that are mainly held together by three coplanar hydrogen bonds. This design minimizes geometric perturbations of the interaction geometries as a function of the substituents introduced in both the naphthyridine and the urea moieties. The data set encompasses 20 intermolecular complexes ranging from 79 to 115 atoms. The association equilibrium constants were determined via NMR titration.^266^ The experimental results were complemented with computed geometries at the B3LYP/6–31G* and M06–2*X*/6–31G* levels of theory. Based on these geometries, the authors performed energy decomposition analysis via SAPT0/aug-cc-pVDZ’ simulations, which indicated significant contributions from both exchange and dispersion. The most favorable π-π stacking contribution to the association free energies in solution was estimated to be −4.2 kcal mol^–1^. The authors concluded that, despite a competition with the solvent, π-π stacking can still be governed by dispersion.

#### 4.2.12 Pollice et al. 2 ^76,254^

This is a combined experimental and computational data set characterizing the importance of London dispersion in proton-bound homodimers of many 2,6-disubstituted pyridines and other related nitrogen bases both in the gas phase and in solution. The central hydrogen bond imposes constraints on the interaction geometries and primes substituents in the vicinity for additional interactions. The data set consists of 38 systems with 23–171 atoms, and provides both experimental association free energies in solution for 37 of the systems and bond dissociation energies in the gas phase for 5 of them. Gibbs free energies in solution were determined based on both variable-temperature ^1^H NMR studies^267^ and isothermal titration calorimetry.^268^ Bond dissociation energies in the gas phase were determined via threshold-collision induced dissociation.^269^ In addition, the data set also consists of computational data based on geometries at the B97-D3/pc-2-sp­(d) level with energies obtained from DLPNO–CCSD­(T)/CBS and solvation energies based on either SMD or COSMO-RS. The authors find that compensation of London dispersion through solute–solvent interactions is large but not complete, providing an estimate for compensation of around 80% in a range of polar aprotic solvents at room temperature. At temperatures as low as −100 °C, compensation is reduced to around 60%, especially in solvents with high bulk polarizabilities.

#### 4.2.13 Wilming et al.^255^

This is a combined experimental and computational data set for the quantification of London dispersion in the double bond geometry isomerization equilibria of 2,2’-disubstituted 9,9’-bifluorenylidenes. The corresponding double bond isomerization activation energy of roughly 26 kcal mol^–1^ enables determination of equilibrium constants via ^1^H NMR spectroscopy by simply integrating the peaks of the protons at the 1,1’,8, and 8’ positions of the bifluorenylidene backbone. Additionally, the rigidity of the bifluorenylidene backbone reduces geometric effects, and the absence of polar groups minimizes attractive contributions from interaction energy components other than dispersion. The data set consists of 13 molecules with 48–91 atoms. The authors also simulated the corresponding geometries at the PBEh-3c level and provide computed free energies of the double bond geometry isomerization at the revDSD-PBEP86-D4/def2-QZVPP//SMD and DLPNO–CCSD­(T)/def2-TZVP//SMD levels of theory. The authors find that the sterically more crowded *Z*-isomer is preferred for the majority of substituents, and the largest Gibbs free energy bias of −0.54 kcal mol^–1^ in benzene is found for the cyclohexyl-disubstituted analogue. Very large substituents, like diamantyl, introduce too much repulsion in the corresponding interaction geometries, tipping the balance to the *E*-isomer with a Gibbs free energy of 0.05 kcal mol^–1^ in benzene, and showing that substituents maximizing dispersion need to be adapted in size to the system at hand.

#### 4.2.14 Wang et al.^256^

The data set from Wang et al. is a robust benchmark specifically designed to evaluate the accuracy and reliability of free-energy perturbation (FEP) methods in predicting relative protein–ligand binding affinities. The data set includes over 200 ligands across eight pharmaceutically relevant targets, with 529 individual binding energies in total. Each target presents unique structural characteristics, covering a range of binding site environments from highly rigid to more flexible binding pockets. The data set is notable for the variety of ligand perturbations it includes, encompassing modifications from simple substitutions to more intricate changes involving up to 10 heavy atoms. Each experimental binding affinity was measured using standardized biochemical assays, supplying a consistent set of high-quality reference data. The predicted affinities for these ligands were generated through a well-defined, automated FEP workflow utilizing the OPLS 2.1 force field, which was later referred to as the FEP+ method.^270^ By providing extensive experimental binding data, this data set has become a valuable resource for benchmarking relative FEP calculations.

#### 4.2.15 Schindler et al.^257^

The authors present a mixed experimental and computational benchmark data set for binding free energy calculations in 8 pharmaceutically relevant protein targets. The data set contains 264 ligands and provides both experimental and computational results for 296 individual binding scenarios. The data set is intended to support binding affinity predictions for chemically diverse compounds. Each entry in the data set includes a ligand, an experimentally derived target structure (often determined via X-ray crystallography), and both calculated and experimental binding free energies. The computed data is based on the FEP+ method that was established in the study discussed in the preceding paragraph.^256^ Alchemical transformations occurring in the data set include changes in net charge, ring openings, and core hopping, reflecting the challenges of real-life drug discovery projects. Accordingly, this chemically varied data set also offers useful data on NCIs for modeling protein–ligand interactions via ML. Schindler et al. used this data to evaluate the predictive accuracy of computational methods against experimental data and obtained root-mean-square errors (RMSEs) below 1 kcal mol^–1^.

### 4.3 Solid State Molecular Systems

Obtaining experimental benchmark data on NCIs in solution is particularly challenging as it requires precise measurement of both interaction energies and geometries. Only a limited number of spectroscopic techniques can determine the interaction geometries of small molecules in solution. Consequently, even when high-quality experimental data on interaction energies are available, corresponding geometries are essentially absent from the literature. Contrarily, the data sets that are based on crystalline solids contain both single-crystal structures and experimental heats of sublimation or computed lattice energies, allowing to capture all NCIs present in the system in full structural detail. The intermolecular interactions within these crystals give rise to a net lattice energy, which is a solid-state equivalent of complexation energy in the gas phase and solution. This energy can be computationally accessed and matched with the value derived from experiment, therefore validating the accuracy of the employed computational method. Below, we discuss several data sets that combine experimental crystal structures with experimental heats of sublimation and lattice energies based on simulations (Table 2).

**Table 2:** Data Sets Covering Noncovalent Interactions in Solid State Systems

Data Set	Description	Structure Source	Energy Source	Size
XTMC43 ^271^	Crystals of complexes of 3*d*-, 4*d*-, and 5*d*-transition metals, both open- and closed-shell. **S Tier**.	X-ray diffraction	Heats of sublimation (experiment) and lattice energies (corrected)	43 crystals
C21 ^272^	Crystals of organic complexes and CO_2_. **S Tier**.	X-ray diffraction	Heats of sublimation (experiment) and lattice energies (corrected)	21 crystals
X23 ^273^	Crystals of organic complexes, CO_2_ and noble gases. **S Tier**.	X-ray diffraction	Heats of sublimation (experiment) and lattice energies (corrected)	23 crystals
ICE10 ^17^	Solid water polymorphs. **S Tier**.	Neutron diffraction	Heats of sublimation (experiment) and lattice energies (corrected or computed at PBE0-D3/PAW)	10 crystals
Bernardes and Joseph^274^	Organic drug-like aromatic compounds. **S Tier**.	X-ray diffraction	Heats of sublimation (experiment)	23 crystals
Schmidt et al.^275^	Crystals of small organic molecules. **S Tier**.	X-ray diffraction	Heats of sublimation (experiment)	30 crystals
POLY59 ^18^	Polymorphic crystals of medium-sized organic molecules. **A Tier**.	Experiment and TPSS-D3/PAW	Lattice energies (TPSS-D3/PAW)	59 crystals

#### 4.3.1 XTMC43 Data Set^271^

The experimental benchmark set XTMC43 (Crystal Transition Metal Complex) comprises meticulously curated experimental X-ray crystallographic geometries and heats of sublimation for crystals of transition metal complexes. The intermolecular interactions covered by the benchmark are generally weak and primarily molecular in nature, including vdW forces, electrostatic interactions, and hydrogen bonding. The XTMS43 data set includes 43 transition metal complexes spanning 3*d*-, 4*d*-, and 5*d*-transition metal elements, with various oxidation and spin states, coordinated by different ligands. Among these complexes, 18 are open-shell and 25 are closed-shell. The unit cells contain between 1 to 8 molecules, with the most common configurations being 2 (21 entries) and 4 (18 entries) molecules per unit cell.

#### 4.3.2 C21 Data Set^272^

The experimental C21 data set serves as a benchmark for NCIs in molecular solids. It is based on experimental sublimation enthalpies and X-ray diffraction-determined crystal geometries. The data set encompasses 20 small- to medium-sized organic molecules with up to 24 atoms, which also contain carbon dioxide. The data set was specifically designed to feature crystals with small unit cell sizes and includes a variety of NCIs, such as π-stacking, electrostatic forces, and hydrogen bonding, with lattice energies ranging within 5–40 kcal mol^–1^. The C21 data set was employed by the original authors to benchmark the performance of several DFT functionals. Later, a subset of C21, termed the **X16**, was used by different authors^276^ to benchmark methods accounting for many-body dispersion. In a follow-up publication, the same group expanded the C21 set with experimental enthalpies for hexamine, anthracene, and succinic acid to create the **X23** set,^273^ which was used for a more detailed assessment of the energetic contributions to the lattice energies. Shortly thereafter, the X23 set was further supplemented to include cohesive energies and lattice parameters for Ar, Kr, and Xe.^277^ Finally, the cell volumes and lattice energies for the X23 benchmark were recently revised to also include the effects of thermal expansion.^278^

#### 4.3.3 ICE10 Data Set^17^

This is a mixed experimental and computational data set of 10 experimentally characterized ice polymorphs. The benchmark contains both crystallographic information derived from low-temperature neutron diffraction and the sublimation enthalpies for every system. Experimental enthalpies are provided for the first 7 members of the data set, while the values for the remaining 3 were derived at the PBE0-D3/PAW level of theory. The 10 polymorphs have 8 to 28 water molecules per unit cell. Among them, only 4 are proton-ordered, that is hydrogen atoms have fixed positions within the crystal structure. The authors used this data set to establish a hierarchy of DFT and semiempirical methods for handling polymorphs with different densities.

#### 4.3.4 Bernardes and Joseph^274^

The authors present 23 experimental enthalpies of sublimation for 18 organic, drug-like aromatic compounds, some of which exhibit polymorphism. In addition to sublimation enthalpies, they also provide the relative stability of the polymorphs based on the corresponding lattice energy differences. These data are utilized to validate molecular force fields.

#### 4.3.5 Schmidt et al.^275^

The authors conducted long molecular dynamics (MD) simulations on 30 small organic molecular crystals. They benchmarked the computed sublimation enthalpies, melting temperatures, and solid densities against experimental data. The initial structures for the MD simulations are based on the experimental X-ray diffraction-derived geometries.

#### 4.3.6 POLY59 Data Set^18^

This is a computational data set of 59 polymorphic crystal structures for five medium-sized organic molecules. Out of these polymorphs, only 9 were obtained experimentally, all others are represented by the computed lowest-energy crystal structures, 10 per molecule. The authors use TPSS-D3/PAW geometries and relative lattice energies as a computational benchmark, which they employ as a reference for other DFT and semiempirical methods they have tested.

### 4.4 Molecular Systems with Heavy Elements

For a long time, the heaviest group of chemically reactive elements, whose involvement in NCIs was systematically studied, were the halogens. These elements attracted significant attention due to the ability to form strong, predictable, and highly directional σ-hole bonds. Such characteristics make halogen bonding a useful design handle in fields like crystal engineering and drug design. As a result, numerous comprehensive benchmarks have been developed for halogen bonding.^209–211^ Recent advancements in computational methods and increased computational resources have led to a growing interest in other types of σ-bonding interactions such as tetrel, pnictogen, and chalcogen bonding, together with a wider range of NCIs involving halogens and other heavier elements. Below, we explore some of the key computational data sets relevant to these emerging areas of research (Table 3).

**Table 3:** Datasets Covering Noncovalent Interactions in Molecular Systems with Heavy Elements

Data Set	Description	Structure Source	Energy Source	Size
CHAL336 ^108^	Chalcogen bonding (O, S, Se and Te). **A Tier**.	PW6B95-D3(BJ)/def2-TZVPD or PBE0-D3(BJ)/def2-TZVPD	W1–F12 or DLPNO–CCSD(T)/CBS	336
Atlas SH250×10 ^279^	σ-hole interactions (halogen, chalcogen and pnictogen), 10-point dissociation curves. **A Tier**.	B3LYP-D3(BJ)/def2-QZVP	CCSD(T)/CBS	250
HEAVY28 ^212^	Noncovalently bound complexes of binary hydrogen compounds primarily stabilized by σ- and hydrogen bonding. **A Tier**.	PBE0/def2-QZVP^220^	CCSD(T)/CBS	28
Atlas HB300SPX^280^	Hydrogen-bound complexes of 10 elements (H, C, N, O, P, S, F, Cl, Br, I). **A Tier**.	B3LYP-D3(BJ)/def2-QZVP	CCSD(T)/CBS	300
Atlas HB300SPX×10 ^280^	Hydrogen-bound complexes of 10 elements (H, C, N, O, P, S, F, Cl, Br, I) with 10-point dossociation curves. **A Tier**.	B3LYP-D3(BJ)/def2-QZVP	CCSD(T)/CBS	300
Atlas D1200 ^281^	London dispersion in dimers; 15 elements (H, B, C, N, O, P, S, F, Cl, Br, I, He, Ne, Ar, Kr). **A Tier**.	B3LYP-D3(BJ)/def2-QZVP	CCSD(T)/CBS	1200
Atlas D442×10 ^281^	London dispersion in dimers; 15 elements (H, B, C, N, O, P, S, F, Cl, Br, I, He, Ne, Ar, Kr), with 10-point dissociation curves. **A Tier**.	B3LYP-D3(BJ)/def2-QZVP	CCSD(T)/CBS	442
RG18 ^212^	Complexes of noble gases. **S Tier**.	TPSS-D3(BJ)/def2-TZVP or PBE0/def2-QZVP^220^ or CCSD(T)/cc-pV(Q+d)^282^	CCSD(T)/CBS	25
Atlas R739×5 ^283^	Repulsive contacts in molecular complexes of 15 elements (H, C, N, O, P, S, F, Cl, Br, I, He, Ne, Ar, Kr, Xe), each has a 5-point dissociation curve. **A Tier**.	BLYP-D3/def2-QZVP	CCSD(T)/CBS	739
Bauzá et al.^284^	σ-hole interactions (halogen, chalcogen and pnictogen). **S Tier**.	MP2/aug-cc-pVTZ or CCSD(T)/aug-cc-pVTZ	CCSD(T)/aug-cc-pVTZ	30
de Azevedo Santos et al.^285^	Chalcogen bonding (S, Se). **S Tier**.	ZORA-CCSD(T)/ma-ZORA-def2-QZVPP	ZORA-CCSD(T)/ma-ZORA-def2-QZVPP	8
Liu et al.^286^	Mainly intramolecular chalcohen bonding (X···Y, where X = O, S, Se, Te and Y = F, O, S, Cl) and O–H contacts. **A Tier**.	B3LYP-D3, ωB97X-D or CCSD(T)/def2-TZVP	B3LYP-D3, ωB97X-D or CCSD(T)/def2-TZVP	56
Setiawan et al.^287^	Pnictogen (N, P, As)-bound homo- and heterodimers. **S Tier**.	ωB97X-D/aug-cc-pVTZ or CCSD(T)/aug-cc-pVTZ	ωB97XD/aug-cc-pVTZ,^287^ and W1–F12 or W2–F12 ^212^	36
Palanisamy^288^	Pnictogen complexes of AsCl_3_, PCl_3_, and NCl_3_ with nitrogen-based donors. **A Tier**.	M06–2X/def2-QZVP	M06–2X/def2-QZVP, MP2/def2-QZVP, and CCSD(T)/def2-QZVP	31

#### 4.4.1 CHAL336 Data Set^108^

This data set is likely the most detailed chalcogen bonding benchmark to date. It features O, S, Se and Te, and includes 336 dimers of varying size. The dimers are categorized into four main groups. The first group, called the CHAL–CHAL subset, consists of 99 dimers where chalcogen-containing species act as both σ-hole donors and acceptors. The second group, the CHAL-π subset, includes 27 complexes characterized by the chalcogen-π bonding, which is distinct from π-hole interactions and involves the σ-hole of the chalcogen center and an unsaturated moiety. The third group, the CHAL–X subset, contains 119 complexes stabilized by chalcogen–halogen interactions. The fourth subset, CHAL–N, consists of 91 dimers with chalcogen–nitrogen interactions. Molecular geometries were preoptimized at the PBEh-3c level of theory followed by final optimization at the PW6B95-D3­(BJ)/def2-TZVPD or PBE0-D3­(BJ)/def2-TZVPD level of theory. The reference complex formation energies were calculated using the W1–F12 method,^289^ or, when W1–F12 was not feasible, the DLPNO–CCSD­(T)/CBS method was used as the most efficient and accurate alternative.

#### 4.4.2 Atlas SH250×10 Data Set^279^

This data set encompasses σ-hole interactions (halogen, chalcogen and pnictogen bonds) from the Atlas data set family.^290^ It consists of 250 complexes involving 7 elements (Cl, Br, I, S, Se, P and As), that interact with diverse electron donors. Each geometry was expanded into a 10-point dissociation curve. Molecular geometries were optimized at the BLYP-D3­(BJ)/DZVP level of theory followed by refinement via B3LYP-D3­(BJ)/def2-QZVP. Reference interaction energies were calculated using CCSD­(T)/CBS. The authors use this data set to benchmark a number of (dispersion-corrected) DFT functionals and semiempirical QM methods.

#### 4.4.3 HEAVY28 Data Set^212^

This data set provides formation energies for noncovalently bound complexes of binary hydrogen compounds, primarily stabilized by σ-hole and hydrogen bonding. It is a subset of the composite GMTKNN55 benchmark^212^ (*vide infra*). The data set features 10 elements (Pb, N, Sb, Bi, O, S, Te, Cl, Br, I) and includes 28 complexes in total. Geometries were either taken from the pre-existing benchmarks at the PBE0/def2-QZVP level of theory^220^ or optimized using the same level with or without effective core pseudopotentials. Reference interaction energies were derived at the CCSD­(T)/CBS level of theory.

#### 4.4.4 Atlas HB300SPX and HB300SPX×10 Data Sets^280^

This data set encompasses hydrogen bonds in molecular complexes from the Atlas data set family.^290^ The HB300SPX data set features 300 hydrogen-bound complexes of 10 elements (H, C, N, O, P, S, F, Cl, Br, I). In the HB300SPX×10 data set, the geometry of each complex was expanded into a ten-point dissociation curve. The geometries were optimized at the B3LYP-D3­(BJ)/def2-QZVP level of theory with the reference complexation energies calculated using CCSD­(T)/CBS. The data sets also contain SAPT0-calculated NCI component energies. The authors employed these data sets to benchmark several dispersion-corrected DFT functionals, along with a selection of semiempirical QM methods.

#### 4.4.5 Atlas D1200 and D442×10 Data Sets^281^

These data sets characterize London dispersion in dimers from the Atlas data set family.^290^ Both data sets include 15 elements (H, B, C, N, O, P, S, F, Cl, Br, I, He, Ne, Ar, Kr). The D1200 set features minimum geometries and was constructed to represent four groups of elements, i.e., HCBNO, PS, the halogens, and the noble gases approximately equally, with 1200 entries in total. In addition, the entries were selected to have interaction energies with at least 40% of dispersion according to the SAPT0 energy decomposition analysis.^291^ The D442×10 data set, where each geometry is expanded into a 10-point dissociation curve, samples from these four groups in almost equal proportions, with a slightly higher number of entries for the noble gases due to a growing number of combinations. All molecular geometries were optimized at the B3LYP-D3­(BJ)/def2-QZVP level of theory, and the reference complexation energies were calculated using CCSD­(T)/CBS. The data sets also contain SAPT0-calculated NCI component energies. The authors used these data sets to benchmark a number of dispersion-corrected DFT functionals and some semiempirical QM methods.

#### 4.4.6 RG18 Data Set^212^

This data sets consists of interaction energies for 25 complexes of noble gases and is a subset of the composite GMTKN55 benchmark^212^ for main-group thermochemistry, kinetics, and NCIs (*vide infra*). The data set features interaction energies for the dimers and trimers of Ne, Ar, and Kr. It also includes Ne and Ar tetramers, the Ne hexamer, and complexes of the noble gases with HF, ethyne, ethane, and benzene. Geometries were either optimized at the TPSS-D3­(BJ)/def2-TZVP level of theory or taken from previously published data sets that employed either PBE0/def2-QZVP^220^ or CCSD­(T)/cc-pV­(Q+d).^282^ Reference complexation energies for dimers were computed using counterpoise-corrected CCSD­(T)/CBS, while the values for all other species were calculated at the CCSD­(T)/CBS level without counterpoise correction.

#### 4.4.7 Atlas R739×5 Data Set^283^

This data set encompasses repulsive contacts in molecular complexes from the Atlas data set family.^290^ It comprises 739 complexes of 15 elements (H, C, N, O, P, S, F, Cl, Br, I, He, Ne, Ar, Kr, Xe) represented by neutral singlet dimers. The geometry of each complex is extended into a five-point dissociation curve that extends into both the repulsive and attractive regions. The equilibrium geometries were derived at the BLYP-D3/def2-QZVP level of theory, and the reference interaction energies were calculated using CCSD­(T)/CBS. This data set was utilized by the authors to benchmark various (dispersion-corrected) DFT functionals and semiempirical QM methods.

#### 4.4.8 Additional σ-Bonding Benchmarks below the Gold Standard

The last five benchmark data sets that we will discuss in this section all encompass σ-hole bonding, but, in contrast to the other benchmark data sets discussed above, use reference energies below the ”gold standard”. Bauzá et al.^284^ conducted a study on 30 complexes featuring halogen, chalcogen, and pnictogen interactions, with elements such as N, P, As, S, Se, F, Cl, and Br. The study evaluated the performance of DFT for describing σ-hole bonding, offering recommendations on the most appropriate methods for different subtypes of interactions. Reference geometries were optimized at the MP2 or CCSD­(T) level of theory using the aug-cc-pVTZ basis set, while reference interaction energies were calculated at the CCSD­(T)/aug-cc-pVTZ level.

The study by de Azevedo Santos et al.^285^ presents a comparable benchmarking effort focused on characterizing chalcogen bonding in 8 model complexes of the form D_2_Ch···A^–^ (Ch = S, Se; D, A = F, Cl). The authors perform a hierarchical *ab initio* and DFT benchmark, with geometries and energies evaluated at progressively higher levels of theory, culminating in ZORA-CCSD­(T)/ma-ZORA-def2-QZVPP as the highest level considered.

The mixed experimental and computational study by Liu et al.^286^ focuses on intramolecular NCIs in 56 heteroaromatic compounds, mainly including chalcogen bonding (X···Y, where X = O, S, Se, Te and Y = F, O, S, Cl) and O–H contacts that are relevant to organic semiconductors. Geometries and electronic properties were calculated using the B3LYP-D3 or ωB97X-D level of theory. For 11 compounds with stronger NCIs, electronic structure calculations were also performed at the CCSD­(T)/def2-TZVP level. The study reveals that orbital interactions play a dominant role in the formation of NCIs in these systems. Additionally, the authors propose a simple geometric descriptor to characterize the strength of these interactions, eliminating the need for high-level computations.

In a study of Setiawan et al.,^287^ a data set of 36 pnictogen-bound homo- and heterodimers, represented as R_3_E···ER_3_ and R_3_E···E*′*R_3_ *′* (E = N, P, As; R = H, CH_3_, C≡CH, BH_2_, OH, NH_2_, F, Cl, Br, I, NO_2_, CN), was investigated to assess the strength of the pnictogen bond using local E···E stretching force constants, which correlate with the degree of charge transfer between donor and acceptor pnictogens. The study examines the forces that stabilize these dimers, including pnictogen bonding, hydrogen bonding, electrostatic interactions, and London dispersion. Molecular geometries were optimized using the ωB97X-D/aug-cc-pVTZ level of theory, with selected structures reoptimized at the CCSD­(T)/aug-cc-pVTZ level, yielding similar results. Dimer binding energies were taken directly from the levels of theory used for geometry optimization. Subsequently, this data set was partially incorporated into the GMTKN55 ^212^ collection as PNICO23, with some modifications. In particular, the authors excluded all dimers containing As, Br, or I and used the geometries of the remaining 23 dimers from the original study. Bonding energies were recalculated at the W2–F12 level of theory, and for the three systems where this level of theory was not feasible, the W1–F12 level was employed instead.

Palanisamy’s study^288^ examines 31 complexes involving AsCl_3_, PCl_3_, and NCl_3_ with various nitrogen-based electron donors. Molecular geometries were optimized using the M06–2X/def2-QZVP level of theory, and interaction energies were computed at the M06–2X/def2-QZVP, MP2/def2-QZVP, and CCSD­(T)/def2-QZVP levels.

### 4.5 Composite Data Sets

In recent years, the development of composite benchmarks has gained momentum, incorporating a wide variety of interactions, often both covalent and noncovalent, and featuring diverse nonequilibrium geometries. These benchmarks are designed to maximize coverage of the potential energy surface and/or chemical space, with a typical data set size exceeding 5000 data points. They may contain brand new data or integrate already existing smaller databases, with or without modifications. Common modifications may include: 1) recalculation of energies at higher levels of theory, 2) augmentation with underrepresented interaction types, 3) expansion of equilibrium geometries into multipoint dissociation curves (considering both radial and angular degrees of freedom) or random sampling of the potential energy surface, and 4) the exclusion of elements that do not fit the common theme of the data set. These data sets may go beyond NCI energies and also incorporate covalent interactions, such as atomization energies, reaction barrier heights, and excitation energies. Such comprehensive scope is particularly beneficial for ML approaches, which sometimes require large amounts of data to achieve high accuracy and precision. Sufficient data also allows for reliable statistical validation and testing through data set partitioning. Below, we outline open-access computational data sets of this type (Table 4), with a focus on their coverage of NCIs.

**Table 4:** Composite Data Sets Covering Noncovalent Interactions in Diverse Molecular Systems with Heterogeneous Sources for Structures and Energies

Data Set	Description	Structure Source	Energy Source	Size
NENCI-2021 ^292^	Intermolecular complexes relevant to both biological and chemical systems, various interactions. **S Tier**.	Counterpoise-corrected MP2/cc-pVTZ and nonequilibrium structures	CCSD(T)/CBS	7,763
OrbNet Denali^293^	Bio- and organic chemical systems, most common biochemical elements (H, Li, B, C, N, O, F, Na, Mg, Si, P, S, Cl, K, Ca, Br and I), neutral and charged molecules, both covalent bonding and NCIs, conformers and nonequilibrium geometries. **B Tier**.	GFN1-xTB	ωB97X-D3/def2-TZVP	2.3 million
QMugs^294^	665,000 biologically- and pharmacologically relevant molecules up to 100 atoms, covering 10 elements (H, C, N, O, S, P, F, Cl, Br, I), three conformers for each. **B Tier**.	GFN2-xTB	GFN2-xTB and ωB97X-D/def2-SVP	2 million
GMTKN55 ^212^	General main group thermochemistry, kinetics and noncovalent interactions. **A Tier**.	Predominantly DFT	Predominantly WFT	1,505
Atlas family^290^	Diverse collection of data sets for various NCIs, covers 18 elements (H, B, C, N, P, As, O, S, Se, F, Cl, Br, I, He, Ne, Ar, Kr, Xe). 2,964 dimers with multiple geometries. **A Tier**.	DFT	CCSD(T)/CBS	19,123
*Masumian and Boese* ^295^	DFT-SAPT interaction component energies for dissociation curves of 19,293 dimers from the Atlas D442×10, SH250×10, R739×5, HB300SPX×10, HB375×10, IHB100×10 data sets, combined with the X40×10 and S66×8 data sets. Charge-transfer interaction energies for HB375×10 and HB300SPX×10 hydrogen bonds. Covers 18 elements (H, B, C, N, P, As, O, S, Se, F, Cl, Br, I, He, Ne, Ar, Kr, Xe). **A Tier**.	DFT	PBE0AC-SAPT and B3LYPAC-SAPT	354,106
DES370 K^296^	General-purpose benchmarks for parametrizing NCI energies, covering 24 elements (H, Li, C, N, O, F, Na, Mg, P, S, Cl, K, Ca, Br, I, Ar, Ne, Kr, Xe) and various motifs including protein functionalities. 3,691 dimers in many nonequilibrium geometries. **A Tier**.	QM or MM	CCSD(T)/CBS	370,959
DES15K^296^	General-purpose benchmarks for parametrizing NCI energies, covering 24 elements (H, Li, C, N, O, F, Na, Mg, P, S, Cl, K, Ca, Br, I, Ar, Ne, Kr, Xe) and various motifs including protein functionalities. 3,258 dimers in many nonequilibrium geometries. **A Tier**.	QM or MM	CCSD(T)/CBS	14,651
DES5M^296^	General-purpose benchmarks for parametrizing NCI energies, covering 24 elements (H, Li, C, N, O, F, Na, Mg, P, S, Cl, K, Ca, Br, I, Ar, Ne, Kr, Xe) and various motifs including protein functionalities. 3.961 dimers in many nonequilibrium geometries. **A Tier**.	QM or MM	SNS-MP2	4,955,938
SPICE^297,298^	Fragments relevant to interactions between drug-like small molecules and proteins, spanning 17 elements (H, Li, B, C, N, O, F, Na, Mg, Si, P, S, Cl, K, Ca, Br and I). 13,999 molecules in many nonequilibrium geometries. **B Tier**.	MM	ωB97M-D3(BJ)/def2-TZVPPD	2,008,628
GEMS^299,300^	Fragments relevant to interactions of proteins, including both covalent and NCI systems, with and without explicit solvation, covering 5 elements: H, C, N, O, and S. **B Tier**.	QM and MM	PBE0+MBD	2,713,986
SO3LR^301,302^	Both covalent and NCI systems, small and large, with and without explicit solvation, covering 8 elements: H, C, N, O, F, P, S, and Cl. **B Tier**.	QM and MM	PBE0+MBD	4 million
Splinter^303^	Benchmark for NCIs in protein–ligand complexes, contains protein-like and drug-like molecular fragments. **A Tier**.	B3LYP-D3/cc-pVDZ or B3LYP-D3/aug-cc-pVDZ	CCSD(T)/CBS	1.5 million
PDB^304,305^	Comprehensive resource of biomolecular structures, including proteins, nucleic acids, and complexes. **S Tier**.	Experimental (X-ray, NMR, cryo-EM) and simulations (AlphaFold, homology modeling)	Experiment	over 170,000 structures
BFDb^306^	NCIs in experimentally measured crystal structures of proteins. **S Tier**.	X-ray diffraction, relaxed with ff99SB41 (MM) in water	CCSD(T**)-F12/aug-cc-pV(D+d)Z	10,972
MISATO^307^	Comprehensive library of protein–ligand interactions, enriched with QM and MD data. **A Tier**.	MM	Experiment	19,443
PLAS-20k^308^	Protein–ligand binding affinities, including MD data. **B Tier**.	MM	MMPBSA	19,500

#### 4.5.1 NENCI-2021 Data Set^292^

The Non-Equilibrium Non-Covalent Interaction (NENCI-2021) data set provides energies for a diverse range of intermolecular complexes that are relevant to both biological and chemical systems. The data set includes 7,763 data points and covers an extensive chemical space, making it valuable for validating computational models and training ML algorithms. The data points of NENCI-2021 represent all major types of NCIs according to SAPT energy decomposition analysis:^291^ electrostatics-dominated (3,499 complexes), induction-dominated (700 complexes), dispersion-dominated (1,372 complexes), or a mixture of multiple prominent interaction energy components (2,192 complexes). The core of the data set comprises 141 small- and medium-sized molecular dimers (containing H, C, N, O, F, P, S, F, Cl, Br, Li and Na), 101 of which were taken from the S66 ^309^ and S101 ^310^ benchmarks, along with another 40 being new cation−π and anion−π complexes. The 101 initial dimers feature a variety of intermolecular bonding motifs, such as intermolecular hydrogen bonds, halogen bonds, ion–dipole and dipole–dipole interactions, π-π stacking, dispersion, and X–H···π interactions, along with a significant number of dimers involving water, which serves as a crucial first step toward generating benchmarks for aqueous environments. Minimum bonding distances were found by using the counterpoise-corrected MP2/cc-pVTZ level of theory. The intramolecular potential energy surfaces of these 141 dimers were then sampled, generating seven distances and nine angles per distance (five angles for ion−π complexes). This provided 7,763 benchmark complexes, whose energies were calculated at the CCSD­(T)/CBS level of theory. The energies span a wide range, from – 38.5 to 186.8 kcal mol^–1^, with an average interaction energy of −1.06 kcal mol^–1^.

#### 4.5.2 OrbNet Denali Data Set^293^

OrbNet Denali is an ML potential for electronic structure calculations in biological and organic chemistry, designed to serve as a direct replacement for ground-state DFT energy calculations. Trained on a comprehensive namesake data set of 2.3 million DFT calculations, it covers the most common biochemical elements (H, Li, B, C, N, O, F, Na, Mg, Si, P, S, Cl, K, Ca, Br and I) and both neutral and charged molecules. The data set encompasses both covalent and NCIs, multiple conformers, and nonequilibrium geometries, aiming to maximize the coverage of chemical space and potential energy surfaces. As far as NCIs are concerned, the OrbNet Denali data set includes 271,084 conformations of 21,735 salt complexes, each involving from 1 to 3 salt molecules. Additionally, it integrates structures from the JSCH-2005 ^311^ benchmark, which encompasses interactions of small model complexes, DNA base pairs, and amino acid pairs, and also the SSI subset of the BFDb benchmark (*vide infra*), which consists of side chain-side chain interactions in free proteins.^306^ The reference interaction energies were computed at the ωB97X-D3/def2-TZVP level of theory.

#### 4.5.3 QMugs Data Set^294^

The Quantum-Mechanical Properties of Drug-like Molecules (QMugs) data set is a rigorously curated benchmark comprising over 665,000 biologically and pharmacologically relevant molecules from ChEMBL^312^ (release 27), which are expanded into three conformers each, totaling approximately 2 million data points. Each compound has an average of 30.6 non-hydrogen atoms with up to 100 atoms in total, covering 10 elements (H, C, N, O, S, P, F, Cl, Br, I). Molecular geometries were optimized using the GFN2-xTB method.^313^ Quantum chemical properties such as orbital energies, dipole moments, partial charges, and bond orders were computed at both the GFN2-xTB and the ωB97X-D/def2-SVP levels of theory. While not explicitly designed for NCIs, QMugs captures implicit NCI patterns, which was utilized to train the Δ-quantum ML model DelFTa^314^ that successfully predicts intra- and intermolecular NCIs in biomacromolecules, including polynucleotides and polymers (*vide infra*). This demonstrates the potential of repurposing quantum chemistry-based data sets for studying NCIs.

#### 4.5.4 GMTKN55 Benchmark^212^

The GMTKN55 is a collection of smaller data sets, an extensively expanded successor to the GMTKN24 ^315^ and GMTKN30 ^316^ databases, designed to survey general main group thermochemistry, kinetics, and NCIs. This benchmark includes 1505 data points and largely builds on previously published benchmarks, incorporating modifications and refinements at higher levels of theory where appropriate. It serves as a valuable and frequently used tool for the development and evaluation of computational methods.^317,318^ GMTKN55 spans a wide variety of chemical reactions and interactions, with 55 distinct subsets tailored to evaluate different types of reactions and molecular systems. These interactions range from covalent bond formation and breaking to weak NCIs. Reference energies and molecular geometries were calculated using various levels of theory, from DFT to WFT, with a preference for the higher-level methods. Many interaction energies are provided at the CCSD­(T)/CBS level, particularly for subsets with NCIs. These subsets target both intra- and intermolecular interactions. Examples are the RG18 and HEAVY28 data sets discussed above, along with others focused on solvent clusters, hydrogen bonding, σ-hole bonding, induction- or dispersion-bound complexes, and the energies of distinct conformers of various compounds.

#### 4.5.5 Atlas Family of Data Sets^290^

The NCIs Atlas project comprises a collection of data sets designed to offer a well-curated, balanced, and diverse representation of various NCI types, including nonequilibrium geometries. It covers 18 elements (H, B, C, N, P, As, O, S, Se, F, Cl, Br, I, He, Ne, Ar, Kr, Xe) at the time of writing (first of October 2024). The Atlas project currently includes seven data sets, featuring 2964 noncovalent complexes expanded into a total of 19,123 geometries, with reference energies computed at the CCSD­(T)/CBS level of theory. These data sets are organized by dominant NCI types. While some of these subsets were discussed previously, the entire Atlas family of benchmarks characterizes the following NCIs: London dispersion in D1200 and D442×10,^281^ σ-hole interactions in SH250×10,^279^ repulsive contacts in R739×5,^283^ and hydrogen bonding extended to sulfur, phosphorus, and halogens in HB300SPX and HB300SPX×10.^280^ The additional data sets HB375×10 and IHB100×10 provide 10-point dissociation curves for hydrogen- and ionically bound organic molecules. Every data set, except for SH250×10 and R739×5, also contains SAPT0-calculated NCI component energies.^319^

#### 4.5.6 Masumian and Boese^295^

This is a comprehensive benchmark based on commonly used NCI data sets, recalculated using the DFT-SAPT energy decomposition scheme, with a particular focus on dissociation curves. The benchmark incorporates geometries from all specialized Atlas data sets (*vide supra*) that include dissociation curves as of the time this work was published (i.e., D442×10, SH250×10, R739×5, HB300SPX×10, HB375×10 and IHB100×10). It is further enhanced with coordinates from the halogen-bonding X40×10 ^320,321^ and the general S66×8 NCI benchmark.^309^ It covers 18 elements (H, B, C, N, P, As, O, S, Se, F, Cl, Br, I, He, Ne, Ar, Kr, Xe), similar to the Atlas data set. The data set provides four interaction energy components, namely electrostatics, exchange, induction, and dispersion, for 19,293 intermolecular dimers across 2312 potential curves with two hybrid xc-potentials, PBE0AC and B3LYPAC, totaling 154,344 data points. For 5778 structures with only first-row atoms and hydrogen, the authors performed additional high-precision calculations and recalculated induction terms for 1000 ionic hydrogen bonds. Charge transfer energies were also evaluated for systems in the HB375×10 and HB300SPX×10 data sets. Altogether, they generated 354,106 quantum chemical data points, categorized by method and interaction type, providing a valuable resource for semiempirical modeling, force field development, and ML applications.

#### 4.5.7 DES370 K, DES15K, and DES5M Data Sets^296^

These data sets are general-purpose benchmarks for parametrizing NCI energies. The core of the DES370 K data set consists of 392 closed-shell monomers, each containing up to 22 atoms, including both neutral species and ions. These monomers were combined into 3,691 distinct NCI-bound dimers, expanded into 370,959 nonequilibrium geometries, which were derived through QM methods or MD. The interaction energies for these dimers were calculated at the CCSD­(T)/CBS level of theory. The data set encompasses 24 elements (H, Li, C, N, O, F, Na, Mg, P, S, Cl, K, Ca, Br, I, Ar, Ne, Kr, Xe) and features various structural motifs, including water and protein functional groups, aiming to cover a broad chemical space. DES15K is a condensed version of the DES370 K data set with 274 monomers, 3258 dimers, 14,651 total geometries and is tailored for more computationally intensive tasks while maintaining comprehensive chemical space coverage. Additionally, DES5M is an expanded version of DES370 K, containing 4,955,938 dimer geometries with energies computed using the SNS-MP2 method,^322^ an ML approach that achieves accuracy comparable to the original coupled-cluster training data by correcting the results of simulations at the MP2/CBS level of theory. Later on, aside from systems with noble gases, DES370 K was partially augmented with forces, charges, multipole information, and bond index information. This augmented data set was integrated into the more holistic SPICE benchmark,^297,298^ which is aimed at training transferrable ML potentials for protein–ligand binding and will be discussed next.

#### 4.5.8 SPICE Data Set^297,298^

This is a quantum chemistry benchmark designed for training ML potentials relevant to simulating the interactions between drug-like small molecules and proteins, sampling from both covalently bound systems and NCIs. The data set encompasses systems ranging in size from 2 to 110 atoms, comprising 13,999 molecules and clusters with a total of 2,008,628 conformations, spanning 17 elements (H, Li, B, C, N, O, F, Na, Mg, Si, P, S, Cl, K, Ca, Br and I). Aside from geometries and energies, the SPICE data set contains forces, charges, multipole information, and bond index information. The current version of the data set^297^ is built upon several subsets for NCIs, which also includes the DES370 K data set^296^ discussed above. It contains various NCIs, in particular explicitly solvated amino acids and drug-like molecules, amino acid-ligand pairs, ion pairs, and water clusters. These subsets were further augmented with forces, charges, multipole information, and bond index information. In addition, covalent information is represented by subsets containing dipeptides and drug-like molecules. Forces, energies, and other properties were calculated at the ωB97M-D3­(BJ)/def2-TZVPPD level of theory. This comprehensive data set provides a valuable resource for developing and testing ML-based models for both covalent bonds and NCIs in drug discovery and computational organic chemistry.

#### 4.5.9 GEMS and SO3LR Data Sets^299,301^

SO3LR is an ML force field designed to enhance molecular simulations by integrating the SO3krates neural network^323^ with universal pairwise force fields. This integration allows SO3LR to efficiently and accurately model both semilocal interactions and universal pairwise forces, covering short-range repulsion, long-range electrostatics, and dispersion interactions. The training set for SO3LR is a comprehensive collection of quantum mechanical data, encompassing both small and large molecules, together with noncovalent systems, both with and without explicit solvation, comprising eight key elements commonly found in biological systems: H, C, N, O, F, P, S, and Cl. This set was built as a combined collection of five data sets: 2.7 million bottom-up GEMS fragments,^299,300^ 1 million QM7-X molecules,^324^ 60k AQM gas-phase molecular drugs (*vide supra*),^249^ 33k SPICE dipeptides (*vide supra*),^297,298^ and 15k DES molecular dimers (*vide supra*).^296^ The first three data sets were computed at the PBE0 level of theory, incorporating a many-body treatment of van der Waals interactions (PBE0+MBD). To ensure consistency in reference data, the last two data sets were recalculated at the same level. While this data set is not directly referenced in Version 2 of the original preprint on ChemRxiv,^301^ it can be found separately on Zenodo.^302^

#### 4.5.10 Splinter Data Set^303^

The SAPT0 Protein–Ligand Interaction (Splinter) data set was developed as a benchmark for NCIs in protein–ligand complexes, with the goal of supporting the development of molecular mechanical and ML methods. An NCI-bound complex in the Splinter set consists of two components: 1) one of 25 protein fragment-like molecules (17 neutral, 4 cationic, and 4 anionic) along with four species commonly associated with proteins (water, Na^+^, Cl^–^, and SO_4_^2–^ ions), and 2) one of 303 ions or molecules (248 neutral, 23 cationic, and 32 anionic, including Na^+^ and Cl^–^ ions) representing moieties and functional groups typically found in or associated with small drug-like molecules. This yields 9463 unique molecular dimers, whose geometries were perturbed to sample relevant regions of the potential energy surface. The SAPT0/jun-cc-pV­(D+d)­Z and SAPT0/aug-cc-pV­(D+d)­Z^291^ levels of theory were then used for characterizing these configurations, partitioning the interaction energy into the interaction energy components: electrostatics, dispersion, induction, and exchange. Only configurations with realistic interaction energy magnitudes were selected, resulting in approximately 1.5 million data points. The optimization of dimer geometries was performed at the B3LYP-D3/cc-pVDZ or the B3LYP-D3/aug-cc-pVDZ level of theory, with final interaction energies calculated using CCSD­(T)/CBS.

#### 4.5.11 Protein Data Bank (PDB)^305,325^

The PDB is a fundamental and comprehensive repository for three-dimensional structural data on proteins, nucleic acids, and other biomacromolecules, containing over 170,000 entries. This extensive data set has enabled in-depth exploration of biomolecular structure and interactions since its establishment. Initially, the PDB focused exclusively on experimentally derived structures obtained via X-ray crystallography, nuclear magnetic resonance (NMR) spectroscopy, and cryo-electron microscopy (cryo-EM). In recent years, however, the repository has expanded to include computationally derived models, especially those produced through advanced structure prediction methods like AlphaFold^326^ or homology modeling. Each entry in the PDB provides atomic coordinates along with extensive metadata detailing experimental conditions or computational parameters. In addition, many PDB entries include experimentally determined binding affinities, which provide valuable quantitative data on NCIs. This data allows for an in-depth examination of NCIs such as hydrogen bonding, salt bridges, van der Waals forces, and π-π stacking. These NCIs are fundamental to maintaining biomolecular structure, stability, and function, playing critical roles in protein folding and intermolecular interactions. The diversity, scale, and detailed annotation of the PDB make it a foundational resource for studying and modeling NCIs across a wide array of biomolecular systems.

#### 4.5.12 BFDb Data Set^306^

The BioFragment Database (BFDb) benchmark samples from noncovalent potential energy surfaces in experimentally measured crystal structures of proteins. Instead of structuring the data set based on a rigid classification of NCI types, the BFDb benchmark provides SAPT energy decomposition results for every interaction. To build the benchmark, the authors sampled the PDB^305,325^ for crystal structures without nonstandard amino acid residues, bound ligands, nucleic acids, or metals, and with a resolution better than 2 Å. This yielded a set of 47 structures, from which the authors removed water, added hydrogen atoms, and relaxed the geometries with the ff99SB41 force field, supplemented with a generalized Born implicit water solvent model. Upon identifying NCIs based on the sum of vdW radii, the protein structures were fragmented around the interaction points, which yielded 10,972 biomolecular fragment interactions. Out of those, 4,640 interactions were between protein backbone moieties (e.g., from α-helices and β-sheets), 3,558 side chain-side chain interactions, and 2,774 backbone-side chain interactions. Out of the backbone interactions, 100 complexes were randomly selected to form the BBI subset, and, out of the side chain-side chain interactions, 3380 were selected for the SSI subset. The respective reference energies were calculated at the ”silver-standard” DW-CCSD­(T**)-F12/aug-cc-pV­(D+d)­Z level of theory. The authors used this data set to benchmark a number of DFT and WFT-based methods for predicting interaction energies.

#### 4.5.13 MISATO Data Set^307^

The Molecular Interactions Are Structurally Optimized (MISATO) benchmark provides a comprehensive library of protein–ligand interactions, derived from 19,443 protein–ligand structures that is enriched with extensive QM metadata and MD information. It aims to capture the dynamic features that are common in protein–ligand interactions. To create this data set, 19,443 protein–ligand structures were sourced from PDBbind,^327^ a comprehensive collection of experimentally measured binding affinities for the protein–ligand complexes. The authors meticulously reviewed and corrected the structures for inaccuracies related to measurement resolution and the absence of hydrogen atoms. QM calculations were then performed on the ligands, with atomic properties determined *in vacuo* and molecular properties calculated *in vacuo*, water, and wet *n*-octanol. The atomic properties include partial charges based on various approaches, atomic polarizabilities, bond orders, hybridization, Fukui indices, and atomic softness. The provided molecular properties encompass electron affinities, chemical hardness, electronegativity, ionization potentials, static *log P*, and polarizabilities. Additionally, 10 ns MD simulations were performed, capturing largely small-scale structural fluctuations of the protein–ligand complexes with occasional larger-scale events. The MD trajectories were also included in the MISATO data set. To demonstrate the utility of the benchmark, the authors trained a GNN model to assess binding pocket flexibility, addressing a previously unexplored challenge.

#### 4.5.14 PLAS-20k Data Set^308^

This data set provides protein–ligand binding affinities and includes MD information akin to the MISATO data set described above, with the same goal of capturing the dynamics of protein–ligand interactions. PLAS-20k features 19,500 protein–ligand structures and is an immediate extension of the PLAS-5k data set,^328^ which contains 5,000 complexes. It consists of binding affinities together with interaction energy components for electrostatics, vdW interactions, and both polar and nonpolar solvation energy, all of which was calculated based on MD simulations using the Molecular Mechanics Poisson–Boltzmann Surface Area (MMPBSA) method. In addition, PLAS-20k includes MD trajectories for every complex, compounding to 97,500 independent MD simulations.

### 4.6 Excited States

NCIs in excited states (ES-NCIs) govern many photophysical and photochemical processes, including fluorescence, phosphorescence, and charge transfer, which play foundational roles in applications spanning organic electronics, photochemistry, and biological systems.^329–331^ These interactions can alter molecular geometries, stabilization energies, and electron densities, fundamentally influencing molecular behavior. Such shifts impact processes ranging from energy transfer in light-harvesting complexes^332^ to charge separation efficiencies in organic photovoltaics.^331^ Due to changes in the electron density upon excitation, ES-NCIs often display different strengths and characteristics compared to ground state NCIs. As seen above, there have been many rigorously conducted NCI studies for ground-state systems with reliable benchmarks, either based on experiments or computed at a high level of theory. For the ES-NCIs, similar studies are significantly underrepresented. Accurately modeling these interactions requires advanced computational methods capable of handling excited-state wave functions, such as time-dependent density functional theory (TD-DFT), perturbation theory-based methods, or even multireference approaches.^333,334^ However, the most reliable of these methods are computationally intensive, often require expert supervision to verify the electronic nature of the computed states, and typically limited to small systems, posing a barrier for large-scale data set generation. Experimentally, characterizing excited-state geometries and interaction energies remains challenging due to the transient nature of these states. Techniques like transient absorption spectroscopy can offer insights, yet often lack the precision needed for systematic data set creation.^335^ Consequently, data sets for ES-NCIs are extremely rare, making them essential for advancing understanding and predictive modeling.

To our knowledge, *Hancock and Goerigk* ^336^ were the first to establish a systematic and reliable benchmark for ES-NCIs. In their study, they generated single-reference wave function dissociation curves that pass through equilibrium geometries for benzene, naphthalene, anthracene, and pyrene excimers in the first excited state. They thoroughly discuss the choice of an appropriate reference method, suggesting that linear-response CCSDR(3)/CBS with a perturbative triples correction is a suitable parallel to CCSD­(T)/CBS for ground-state calculations. However, due to the prohibitive computational cost of CCSDR(3)/CBS for larger systems, they benchmarked the faster SCS-CC2/CBS­(3,4), where CBS­(3,4) denotes extrapolation using triple- and quadruple-ζ basis sets, as the next best option for the excimer of the benzene dimer. The authors also note that BSSE correction methods have not yet been developed for excited states. Hence, their SCS-CC2/CBS­(3,4) energies are uncorrected for the BSSE. The authors try to partially compensate for that by using large basis sets and performing CBS extrapolations. However, they also point out that the CBS extrapolation techniques were originally intended for ground-state problems and remain untested for ES-NCI energies. Using their benchmark data set, the authors assess the performance of various TD-DFT methods, both with and without ground-state dispersion corrections. They observe that while ground-state-parametrized dispersion corrections reduce the errors in functionals that do not account for dispersion, they generally fall short of achieving chemical accuracy, likely because these corrections are not designed for excited states. Their results highlight that some of the more recent TD-DFT methods, particularly range-separated double hybrids such as ωB2PLYP and ωB2GP-PLYP, are among the most robust and accurate approaches for excited states. This aligns well with findings from benchmarking excited state methods for individual molecules.

A follow-up study from the same group^337^ expands this benchmark by applying the same level of theory, SCS-CC2/CBS­(3,4), to five additional excited-state complexes (exciplexes), namely benzene–neon, benzene–argon, benzene–naphthalene, toluene-tetracyanoethylene, and styrene-trimethylamine. The authors also provide a qualitative energy decomposition of the ES-NCI binding energies into electrostatic, polarization, Pauli exchange, and charge transfer components using absolutely localized molecular orbitals (ALMO-EDA), despite known limitations of this approach.^338^

### 4.7 Limitations

As evident from the numerous data sets discussed above, a substantial amount of data relevant to modeling NCIs already exists in the literature, albeit scattered and heterogeneous in both source and format. This highlights the need for a coordinated effort by the NCI research community to clean up and harmonize this data, transforming it into a unified framework that can drive the development of advanced ML approaches and accelerate NCI research. The limited consolidation attempts underscore a significant challenge, as harmonization is essential for reliable model training and meaningful comparison across studies. Additionally, data harmonization and curation will facilitate the use of these data by nonchemists, particularly computer scientists and ML experts interested in developing foundation models with a broad set of applications in chemistry.

Recent computational data sets show a clear trend toward large-scale collections with millions of entries, specifically designed for training ML models. They enrich ground-state structures with nonequilibrium conformations, effectively sampling the relevant regions of the full potential energy surface. A major challenge in producing such large data sets is balancing the quality of simulations with the required computational resources. As a result, most of these extensive data sets do not employ the ”gold-standard” computational methods for computing interaction energies. Nevertheless, to drive the progress in the field, effective strategies are needed to create ”gold-standard”-quality data sets with millions of data points for medium- to large-sized molecules containing more than 30 atoms.

Apart from the need to reconcile data set size with data accuracy and precision, most available data sets focus on a similar molecular space: closed-shell organic molecules and interactions relevant to biomolecules and biochemistry. Systematic data sets for NCIs in open-shell organic molecules are essentially absent in the literature, despite the significance of these molecules in organic materials^339–341^ and the unique reactivities and functions such interactions facilitate.^342–345^ Additionally, while protein–ligand data sets provide valuable interaction insights, high-quality binding affinity data, especially experimental values, are scarce and often limited to select target classes, leaving gaps in training data for certain interaction types. For instance, protein–ligand data sets cover hydrogen-bonding, ion pairing, and van der Waals interactions comprehensively, however, they include σ-hole and π-hole interactions to a lesser extent. Additionally, experimental data on molecular systems with hole interactions and heavy elements are very scarce, leaving quantum chemistry simulations as essentially the only viable source of reference information.

Additionally, the systematic benchmark data sets discussed thus far focus primarily on main group elements, leaving NCIs that involve transition metals and lanthanides largely unexplored. Transition metals, such as palladium, rhodium, and iridium, serve as powerful catalysts, while platinum-based complexes are also widely used in therapeutic applications,^346,347^ and metals like iron, manganese, and copper are central to biochemistry.^348^ Furthermore, transition metals give rise to distinctive NCIs, such as metallophilic interactions.^349^ This lack of data is true for both experimental and computational data sets. Therefore, new NCI data sets that include these elements would fill a crucial gap, enabling more accurate modeling, understanding of their characteristic interactions, and application of ML approaches to more diverse problems.

Finally, while some recent systematic benchmarks characterize NCIs in excited states (*vide supra*), we believe this remains a severely underdeveloped research area that would benefit greatly from the availability of comprehensive data sets for ML model development. To our knowledge, systematic experimental data sets for NCIs in excited states essentially do not exist. This is particularly critical as the accuracy of excited state quantum chemistry simulations is somewhat less well studied compared to the ground state due to the smaller number of experimental reference data sets, making it more complicated to assess the accuracy of computational reference data. Hence, further work is needed to provide a reliable foundation allowing for the application of ML to model NCIs in excited states.

## 5 Noncovalent Interactions as Features

Proper feature selection directly impacts the ability of models to generalize and make accurate predictions. In the context of NCIs, this means ensuring that the features can meaningfully represent the noncovalent forces. Poor or incomplete feature selection can lead to the model missing critical factors, resulting in inaccurate predictions, overfitting, and loss of interpretability. Below, we review the principal methods for featurizing NCIs in the literature, organized by the level of abstraction: *direct*, where each individual NCI is explicitly encoded; *condensed*, where interactions of the same type are grouped under one or a few descriptors that measure the propensity of the system toward a certain type of NCI; and *global*, where the overall propensity of the system for NCIs of any type is captured under unified metrics. While this classification is to a certain extent artificial, it provides a useful framework for systematically evaluating the strengths and limitations of different featurization approaches. Each of these method offers unique insights. Direct featurization captures the precise contributions of specific interactions, condensed featurization simplifies these into representative systemic descriptors for efficiency, and global featurization assesses the cumulative effect of all NCIs, offering a holistic perspective. Below, we discuss each of these approaches in detail, considering their applications, benefits, and potential drawbacks for ML models aimed at predicting and interpreting NCIs.

### 5.1 Direct Featurization

A direct method for featurizing NCIs involves encoding each known interaction into a specific representation. This could take the form of a list of atomic contacts within a defined radius to capture NCIs based on spatial proximity, a set of geometric, energetic, or other parameters characterizing each interaction, or even a simple binary vector that indicates the presence or absence of particular NCIs without going into the geometric or physical details. This allows for a detailed and systematic representation of interactions, preserving the individuality of each specific contact.

Such a featurization approach was used by the so-called PotentialNet Spatial Graph Convolution neural network,^350^ which was designed for predicting protein–ligand binding affinities. It expands the notion of adjacency by transforming the adjacency matrix *A* into a tensor of shape *N* × *N* × *N*_*et*_, where *N*_*et*_ accounts for the various edge types representing both covalent bonds and NCIs. The authors note that adjacency can also be defined using a distance threshold, where atoms within a specified proximity are considered adjacent. This approach eliminates the need for domain-specific knowledge about interaction types, making the model less reliant on expert input and more agnostic to the underlying physics. In complex systems like protein–ligand complexes, the adjacency matrix can be viewed as a block matrix, as illustrated in Equation 1, with diagonal blocks representing interactions within the same group of atoms (e.g., ligand–ligand, *A*_*L*:*L*_, or protein–protein, *A*_*P*:*P*_, interactions), and off-diagonal blocks capturing interactions between different groups (e.g., protein–ligand, *A*_*P*:*L*_, interactions). Intramolecular NCIs are ignored in this work.

$$A=\left(\begin{array}{cccc} A_{11} & A_{12} & \cdots & A_{1N}\\ A_{21} & A_{22} & \cdots & A_{2N}\\ \vdots & \vdots & \ddots & \vdots \\ A_{N1} & A_{N2} & \cdots & A_{NN} \end{array}\right)=\left(\begin{array}{cc} A_{L:L} & A_{P:L}\\ A_{P:L} & A_{P:P} \end{array}\right)$$ 1

The convolution procedure in PotentialNet consists of the following steps: 1) covalent-only propagation, where the information spreads separately within local covalently bound networks *A*_*P*:*P*_ and *A*_*L*:*L*_; 2) further covalent propagation, but now supplemented by the noncovalent one, where the protein and the ligand ”learn” about each other with the information traveling across the full adjacency tensor *A*; 3) a graph gather operation exclusive on the ligand, where the feature maps of the ligand are derived based on both the covalent bonding and the spatial proximity to the protein. Thus, the deep learning-focused PotentialNet reaches state-of-the-art performance for affinity prediction, comparable to the more physics-based RF-Score,^351,352^ X-Score,^353^ and TopologyNet^354^ models.

A similar featurization approach was later adopted by the authors of InteractionNet,^355^ which is another GNN aimed at predicting protein–ligand interactions. Unlike PotentialNet, which aggregates both covalent and NCIs as edge features in a single graph, InteractionNet introduces two distinct convolution layers, one dedicated to covalent bonding and the other to NCIs (cf. Figure 5). This separation allows for an independent assessment of how each interaction type influences the predictions of the model. When combined with Layer-Wise Relevance Propagation,^356^ InteractionNet offers detailed insights at the atomic level, enabling the identification of the factors affecting predicted binding affinities. While the separate treatment of interactions adds to the explainability of InteractionNet, it displays similar performance compared to PotentialNet on the benchmark data sets tested. A similar two-layer convolution approach was employed in CocrystalGCN,^357^ a graph convolutional neural network (GCNN) developed for predicting cocrystal formation. CocrystalGCN marginally outperformed the baseline methods including RF, SVM, and XGB, achieving roughly 80% in accuracy and precision.

**Figure 5:**
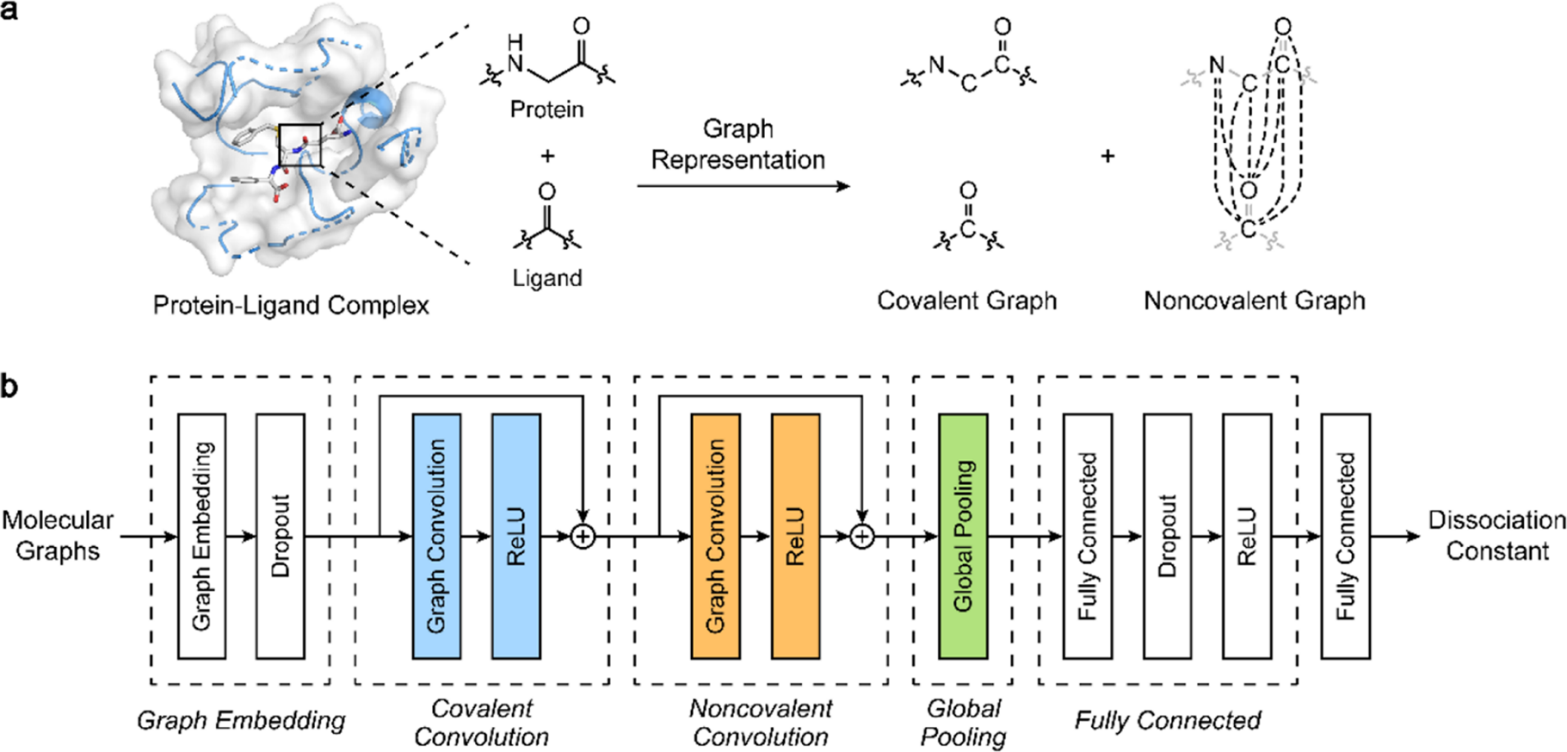
Schematic graph representation within InteractionNet (a) and its architecture (b) for predicting ligand dissociation constants.^355^ Both covalent and noncovalent interactions are encoded into the corresponding adjacency matrices, which are then learned by InteractionNet to yield the dissociation constants.

Torng and Altman^358^ introduced a GCNN framework aimed at predicting drug-target interactions by representing both protein pockets and small molecules as graphs. Unlike previously mentioned models that encoded only individual atoms as graph vertices, this approach encodes both individual atoms in small-molecule ligands and entire protein residues within binding pockets. With graph edges denoting spatial proximity, this approach allows for flexible pocket sizes and weak geometric constraints. (cf. Figure 6) The model operates in two stages: 1) an unsupervised graph autoencoder, trained on a set of protein binding sites, generates fixed-size latent embeddings to capture general pocket features, and 2) two supervised GCNNs are trained separately on pocket and ligand graphs, using binding classification labels for supervision. This setup facilitates the automatic extraction of interaction-specific features without relying on preformed protein–ligand complexes. The ligand is represented as a molecular graph, where each atom node is characterized by a 62-dimensional feature vector, including a one-hot encoding of element, atom degree, number of attached hydrogen atoms, implicit valence, and an indicator for aromaticity. Bond descriptors, encompassing six features, represent bond type (single, double, triple, or aromatic), conjugation status, and ring membership, capturing essential structural and electronic properties. In head-to-head comparisons, the GCNN approach performed on par with traditional and other ML-based scoring methods across benchmark data sets. Visualizations of importance scores for protein pockets and ligands provided interpretative insights by identifying residues and atoms most critical for binding interactions, further highlighting the capability of the model for interpretability and effective feature utilization in predicting binding.

**Figure 6:**
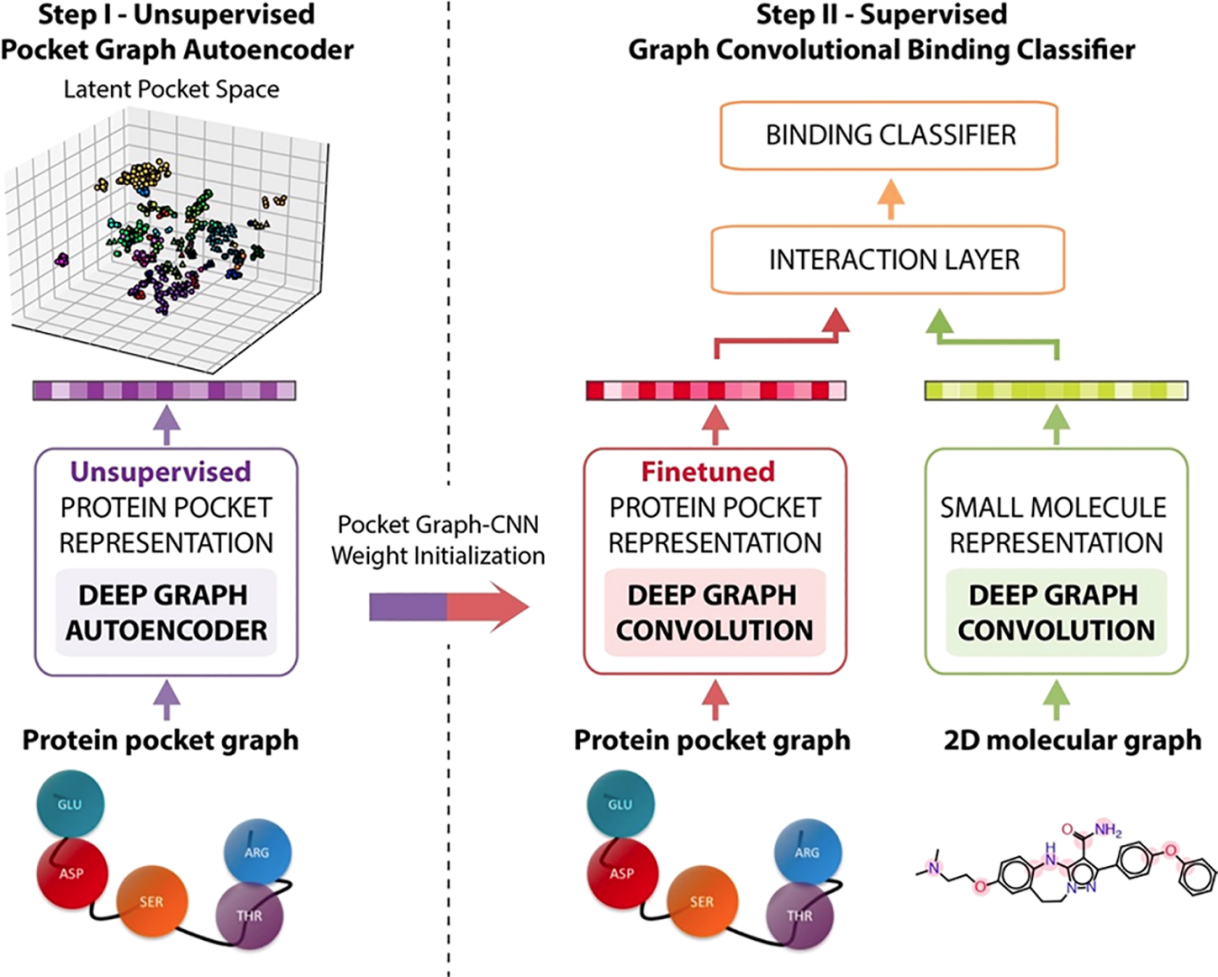
Graph Convolutional Neural Network (GCNN) framework for drug-target interaction prediction by *Torng and Altman*.^358^ The framework represents protein pockets and small molecules as graphs to predict binding interactions. In Step I, an unsupervised deep graph autoencoder learns fixed-size latent embeddings from protein binding sites, capturing general pocket features without geometric constraints. In Step II, two supervised GCNNs are trained separately on the protein pocket and ligand graphs, with binding classification guiding the training. The protein pocket graph represents residues as nodes with edges indicating spatial proximity, while the ligand is represented as molecular graph. The figure was adapted with permission from the literature.^358^ Copyright 2019 American Chemical Society.

Instead of using graph-based representations, the model of Ragoza et al.^185^ directly employs electron density to encode NCIs within protein–ligand complexes, followed by a 3D CNN, focusing on accurate binding affinity predictions. The authors achieved this by discretizing the density of the protein–ligand structure into a 3D grid centered around the binding site. Each point on this grid contains information on atom types, represented across channels analogous to RGB channels in image data, with 34 atom types (16 for receptors and 18 for ligands). The grid covers a 24 Å^3^ area at a 0.5 Å resolution, capturing the spatial distribution of the electron density of each atom using a combination of Gaussian and quadratic functions. This configuration effectively translates the three-dimensional structure into a machine-interpretable format while preserving the continuous density variations around each atom center. The model also utilized data augmentation techniques like random rotations and translations to improve generalization and robustness. This CNN-based scoring function was trained to classify binding poses as either favorable or unfavorable by predicting binding affinities, using a supervised approach that incorporated both positive and negative examples based on ligand–receptor binding poses. The model outperformed traditional scoring functions, particularly in tasks related to pose prediction and virtual screening, highlighting the utility of spatial and physicochemical encoding in deep learning models for NCI interactions.

An alternative direct way to represent NCIs as atomic-contact vectors was used to predict stereoselectivity of Diels–Alder and Michael additions (Figure 7A-B).^359^ In these reactions, a general guideline stipulates that the attack occurs on the less crowded face, which is helpful only when there is a significant difference in steric bulk between the two faces. When both faces have substituents of similar bulk, the situation becomes more complex. In order to resolve this, the authors constructed two transition state-like pairs of starting materials, one corresponding to the experimentally observed product and another to the one that is not (Figure 7C.I). Next, the contacts between reactant atoms were binned according to interatomic distances (2.0–2.5, 2.5–3.5, 3.5–4.5, 4.5–5.5 and 5.5–6.5 Å) and the atom types involved: C_sp^3^_-C_sp^3^_, C_sp^2^_-hydrogen, C_sp^3^_-heteroatom_sp^2^_, etc. (Figure 7C.II). These contacts were then counted and represented as atomic-contact vectors (Figure 7C.III), which were used as input features in an RF classifier. Overall, this led to 89.9% prediction accuracy for Michael additions and 92.0% for Diels–Alder reactions. The authors show that their atomic-contact vectors lead to predictions superior by at least 10% compared to featurization with force field energies and either extended connectivity or three-dimensional fingerprints. Additionally, the accuracy of human expert predictions was also evaluated and was within 40–60% with an average score of 52%.

**Figure 7:**
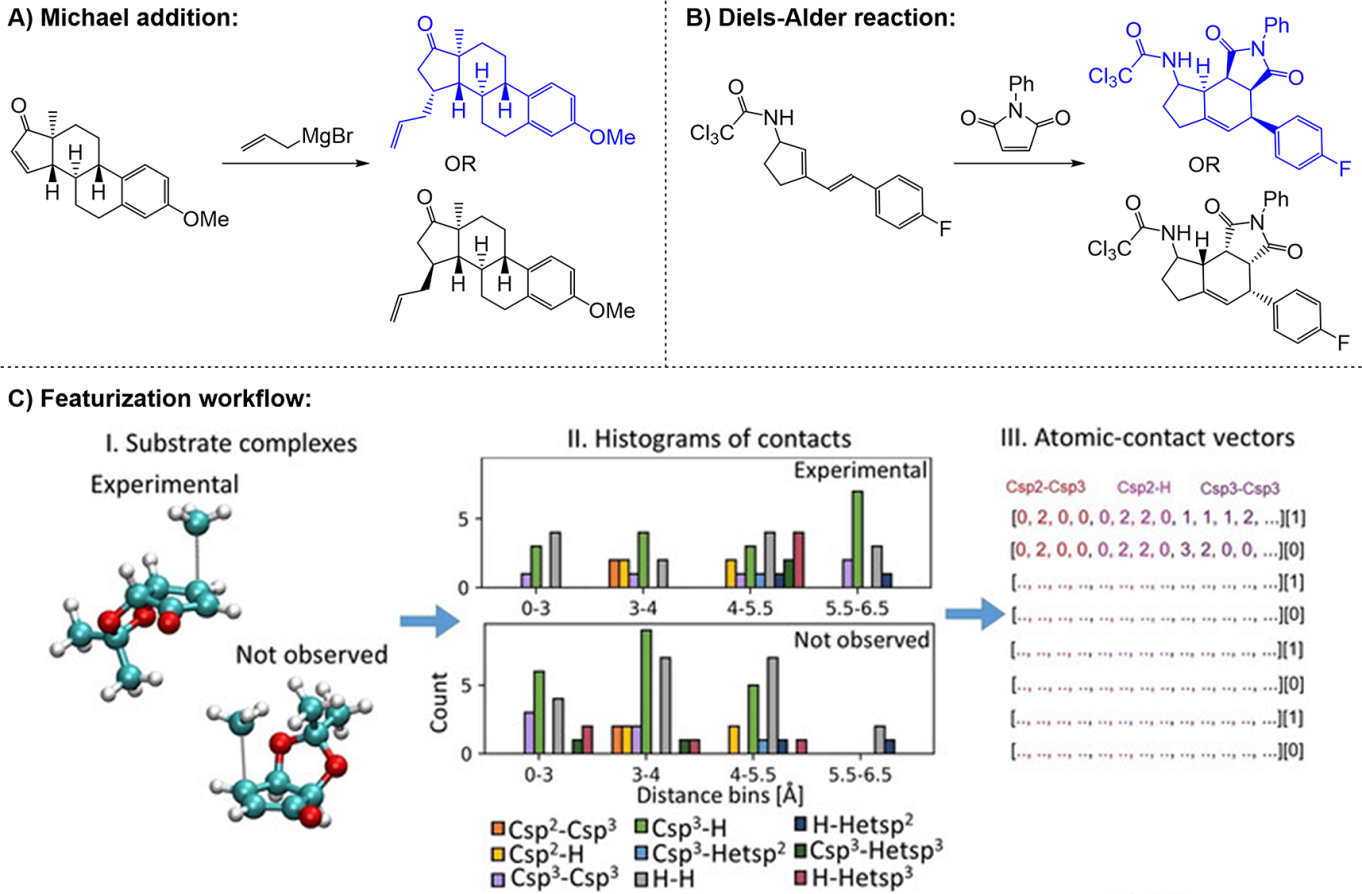
A) Example of a Michael addition. B) Example a Diels–Alder reaction. Experimentally observed products are shown in blue. C) A workflow for deriving atom-contact vectors,^359^ which starts from (I) a pair of transition state-like complexes, followed by (II) distance-binning of contacts between specific atom types and (III) condensing each histogram into a vector, two per reaction. Subfigure C was adapted with permission from the literature.^359^ Copyright 2021 Wiley.

A more geometric featurization approach was used by Liu et al.,^286^ by defining a chalcogen interaction propensity descriptor *S* based on the computational analysis of 56 di- and triheteroaryl systems (Figure 8). Their metric is a function of several geometric parameters. For an X···Y interaction, *S* depends on Δ*d* – the deviation from the sum of vdW radii, α – the angle of alignment between the *XY⃗* vector and the distal σ-bonding axis of X, and Θ – the dihedral angle between the aromatic planes. In case of triheteroaryls, when 0°≤Θ≤ 90°, an X···Y interaction is observed, otherwise when 90° < Θ ≤ 180°, an X···H contact is present. The authors find a remarkably strong correlation (*R* ^2^ = 0.992) between *S* and the second-order NBO perturbation energy for the X···Y chalcogen bonds, modeled at the CCSD­(T)/def2-TZVP//ωB97XD/def2-TZVP level of theory. Along with very low electrostatic attraction energies, this implies the predominant orbital nature of the X···Y interactions. Subsequently, the authors used *S* as a convenient proxy for the NCI strength. They linked *S* to the optical and charge reorganization energy of the studied structures, and thus the optical properties themselves. Validation of the predictive power of *S* across 12 additional heteroaryls with literature-documented solid-state structures confirms that this metric can be easily accessed computationally, while possessing virtually the same descriptive power as the values derived from experiment.

**Figure 8:**
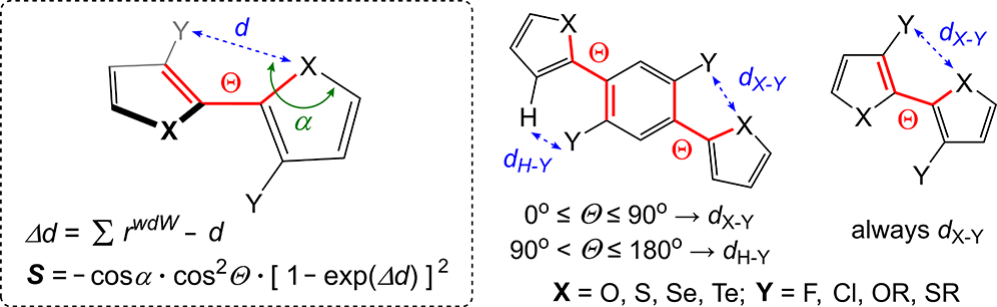
Intramolecular chalcogen interaction metric *S*.^286^

Sigman and co-workers^360^ developed a method to featurize aromatic π-interactions. Regardless of the configuration of the interaction, whether sandwich or T-shaped (Figure 9A), two key features are considered: the distance between the centroids of the interacting rings (*Dπ*) and the interaction energy (*Eπ*), typically computed using DFT. When an NCI can occur in multiple conformations, the associated properties are Boltzmann-weighted according to their energies, as illustrated in Figure 9A. The resulting parameters were denoted as *Dπ*_*w*_ and *Eπ*_*w*_. The authors showcased the effectiveness of their metrics in two case studies: the kinetic resolution of benzyl alcohols and the enantiodivergent fluorination of allyl alcohols.

**Figure 9:**
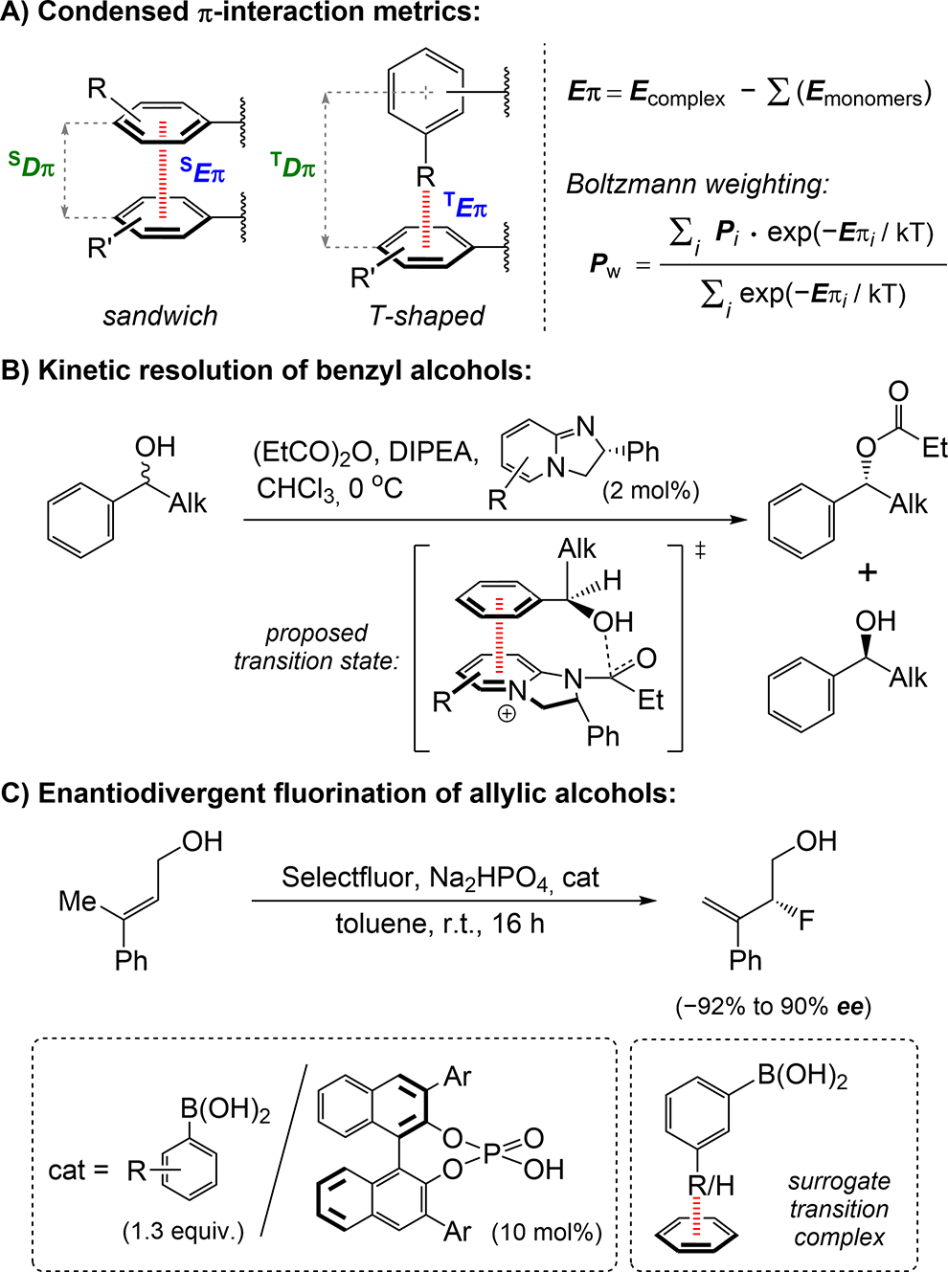
Condensed π-interaction metrics (A) and example use cases (B–C).^360^

In the first case, the use of a chiral catalyst leads to a kinetic resolution, that is, acylation of only one enantiomer, while another stays intact (Figure 9B). In the original study on this reaction,^361^ π-interactions were demonstrated to play a pivotal role in stabilizing the stereoselectivity-determining transition state. Sigman and co-workers used the B97-D/def2-TZVP level of theory to calculate 28 pretransition-state π-stacked complexes for reactions with known experimental outcomes. By applying MLR and robust cross-validation, they developed a model with an *R* ^2^ = 0.92, where the key descriptors were ^*S*^ *Dπ*_*w*_, the cross-term ^*S*^ *Dπ*_*w*_^*S*^ *Eπ*_*w*_, and the Sterimol parameters of the substituents on the benzylic alcohol. An analysis of the coefficients of the model suggests that the enantioselectivity is primarily driven by π-stacking interactions, with steric effects playing a secondary role, which aligns well with the results of the original study.

In the reaction selected as their second case study, the enantiodivergent fluorination of allylic alcohols (Figure 9C), the role of NCIs in determining enantioselectivity had not been well established yet. However, it was hypothesized that the transition state might involve a π-stacking interaction between the boronic acid and the chiral 1,1’-binaphthyl phosphate.^362^ To investigate this hypothesis, 18 T-shaped surrogate transition state π-complexes (Figure 9C) were simulated for various boronic acids using the level of theory described earlier. One molecule of benzene served as a probe and a substitute for the bulky 1,1’-binaphthyl phosphate. Preliminary analysis of the enantioselectivity data showed a strong correlation with a single descriptor, ^*T*^ *Dπ*_*w*_ (*R* ^2^ = 0.77), supporting the relevance of T-shaped interactions in the transition state. Subsequently, a more comprehensive multivariate linear regression yielded a model with an *R* ^2^ of 0.84, which, in addition to ^*T*^ *Dπ*_*w*_, included the Sterimol metrics of the boronic acid and the phosphate, together with the symmetric stretching intensity of the phosphate, which was hypothesized to represent the ability to engage in both hydrogen bonding and electrostatic interactions. Previous efforts to correlate the measured enantioselectivities using MLR were unsuccessful, however, the introduction of the new NCI parameters supplied the missing element needed to explain the key factors driving the reaction and deliver a useful predictive model. The benzene ring introduced into a surrogate transition state was not used to encode any specific NCI but rather to characterize the general propensity of boronic acids toward π-interactions. Consequently, this featurization approach might be better classified as ”condensed.” However, due to the close connection between the applications originating from the same metrics, we have chosen to discuss them together in one section instead of spreading them over two distinct sections.

### 5.2 Condensed Parameters

Rather than encoding individual NCIs separately, a generalized parameter can be selected that correlates with the properties of interest. Condensing individual interactions of the same type into a broader descriptor has the potential to encode both local and global effects that influence reactivity, stability, and selectivity, providing a more efficient and scalable way to represent, and ultimately predict, the NCIs of interest.

Such condensed parameters are often relevant in the realm of steric effects, which can be rationalized based on electrostatics, exchange, and dispersion, and thus are effectively a specific type of NCI that is often predominantly viewed as repulsive. Condensed metrics characterizing steric effects can provide a straightforward way to capture the overall shape and volume of a molecule, the proximity of reactive sites, and the likelihood of steric repulsion. Alternatively, they can sometimes also capture attractive NCIs. Multiple different schemes can be used to gauge steric effects. Purely energetic approaches include energy decomposition analyses,^291,363–367^ experimental or computational enthalpies,^368^ and calculations of force field potential energies.^369^ Electron density-based methods aim to define the concept of steric repulsion through dedicated quantities and quantum topology.^370–372^ Structural methods focus on molecular geometry and the spatial arrangement of substituents, resulting in metrics like the Tolman cone angle,^373^ the percentage of buried volume (%*V*bur),^374,375^ and the Sterimol parameters.^376–378^ Finally, various experimental descriptors link observable properties to steric effects. Among those are molar refractivity^379^ and kinetic difference to a benchmark reaction without the corresponding substituent (Winstein–Holness^380^ and interference parameters^381,382^ ), often utilized for deriving linear free energy relationships (Taft^383–385^ and Charton^386–388^ parameters). Below, we examine how some of the aforementioned condensed parameters and other related metrics have been incorporated into ML models for predicting catalyst performance and chemical reactivity.

The Charton parameter (υ)^386–388^ for an arbitrary substituent R is defined as the difference between the minimal vdW radius of R and that of a hydrogen atom (1.20 Å). It is based on the same reaction as the Taft *E*_*S*_ constants^383–385^ (i.e., acid-catalyzed hydrolysis of methyl esters), and was found to linearly correlate with the logarithm of the hydrolysis rate constant (*log k* in Figure 10A). Importantly, υ can only be directly determined for groups of the form AB_3_ (such as CH_3_, CCl_3_, CMe_3_) or single atoms (like H, F, Cl, Br). However, by leveraging the established correlation between *log k* and υ, one can estimate υ for groups that do not allow for a direct calculation. The Charton parameter has demonstrated strong predictive ability for enantiomeric excess in various asymmetric catalytic reactions, including the allylation of carbonyl compounds, alkene protonation and aziridination,^389^ iridium-catalyzed asymmetric isomerization of primary allylic alcohols,^390^ and 1,2-sulfone rearrangement.^391^ It is worth noting that the Charton parameter is one of the most studied and well-documented metrics of steric effects, especially compared to the alternatives discussed below.

**Figure 10:**
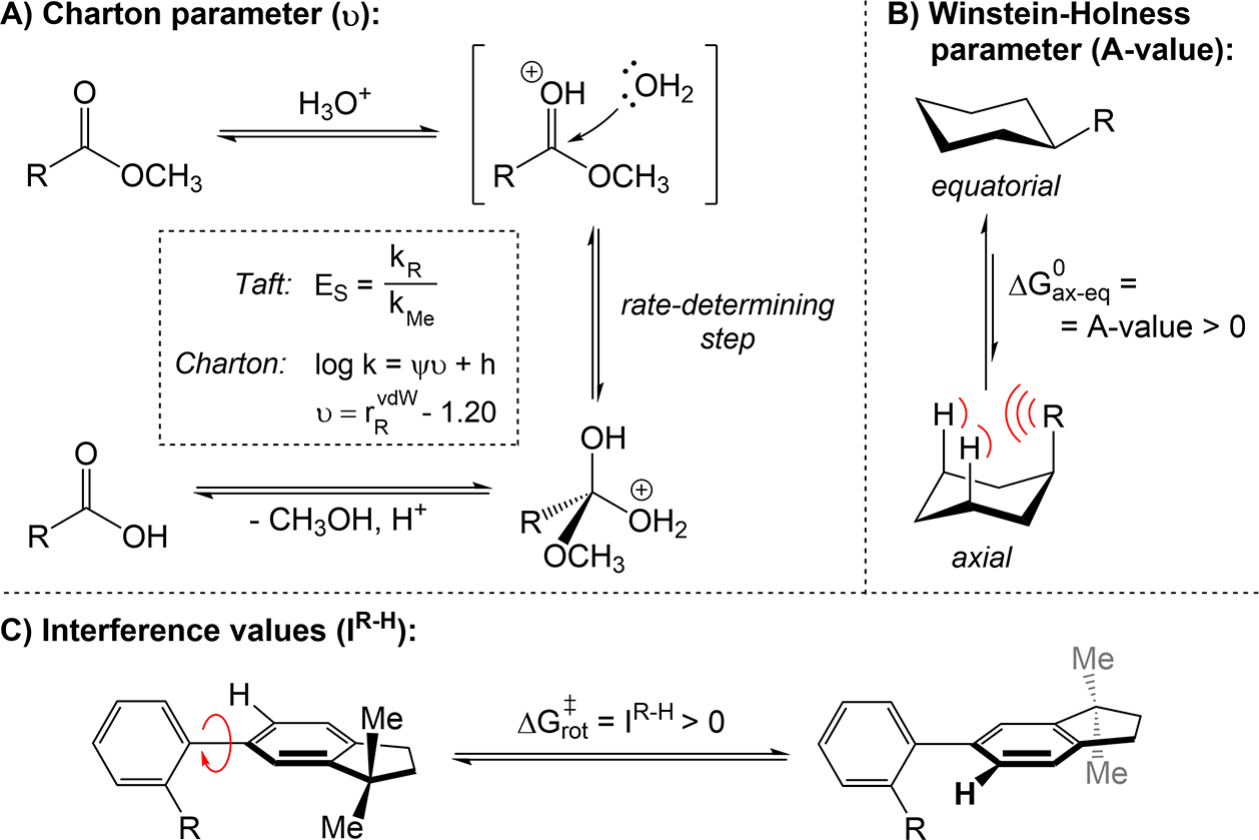
Common steric parameters.

However, as demonstrated by Sigman and co-workers,^392^ the Charton parameter has several limitations. While it correlates well with the enantioselectivity of catalytic desymmetrization in substituted diarylmethanes for certain bulky groups, it fails to result in a linear trend for others. To address this shortcoming, the authors incorporated alternative metrics, such as the Winstein-Holness A-values^380^ and interference values.^381,382^ The A-values describe the free energy difference between axial and equatorial conformers of substituted cyclohexanes (Figure 10B), while interference values represent the rotational barrier in substituted 2,2’-biphenyls (Figure 10C). These parameters provided somewhat stronger linear correlations, underscoring the importance of selecting appropriate metrics when modeling steric interactions, especially when relying on linear models.

This discussion was continued in another study from the same group,^393^ where the authors outline why the Charton parameter may sometimes be inadequate. To reiterate, it is based on the average minimal vdW radius, averaged over rotation. This approach works well when the rate of rotation around the bonding axis is much faster than the reaction rate, allowing for the bulky group to be approximated as a symmetric object, a solid of revolution. However, in enantioselective reactions, where product distributions depend on the relative energies of diastereomeric transition states, this approximation becomes unreliable. This is especially true for larger groups, which often exhibit significant anisotropy along their three principal axes. Moreover, the authors compared the Charton parameter to other steric descriptors like A-values, interference values, and Tolman cone angles, and found a general collinearity between them. This suggests that, to some extent, the choice of steric parameter may not significantly impact the results, provided reliable reference data are available. This realization highlighted the need for a generalized steric parameter that would account for the anisotropy of chirality-inducing groups. To that end, the authors reintroduced the Sterimol parameter, first proposed in the 1970s by Verloop et al.^376,377^

Sterimol is a structural descriptor based on a space-filling model of a molecule, where each atom is treated as a sphere with its respective vdW radius. Rather than a single value, Sterimol consists of several subparameters that capture various geometric aspects of a functional group. In its initial form,^376^ Sterimol employed five parameters. *L* represents the length of the group, measured along the axis of the bond connecting the substituent to the rest of the molecule (R–C bond in Figure 11). B_1_, the smallest width of the group, is measured perpendicular to the R–C bond axis. This parameter is essential for capturing the proximal steric effects close to the R–C bond, where steric hindrance could influence the reaction mechanism. B_2_–B_4_ describe the widths at progressively greater distances from the R–C bond axis (but still perpendicular to it), offering a detailed profile of how the steric bulk of the substituent evolves as one moves further from the bond. However, in the original study, the authors noted a moderate correlation between the B_2_ and B_3_ parameters and a weaker one between each of the B_2_ and B_3_ parameters and B_1_, suggesting that these additional quantities hardly contribute any new information. Because of this redundancy, later refinements^377^ replaced B_2_–B_4_ with B_5_, which represents the maximum width of the substituent, complementing B_1_ (the minimum width) and simplifying the resulting steric representation.

**Figure 11:**
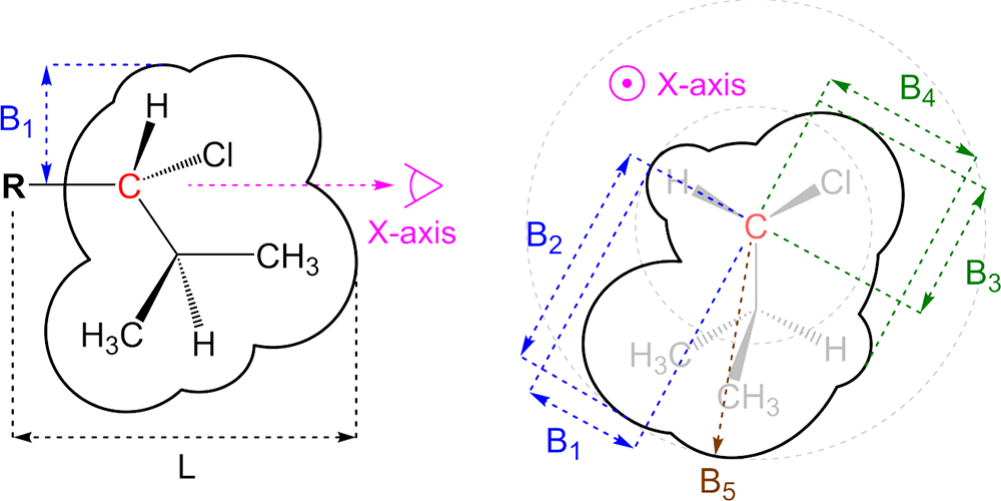
Parameters considered in the Sterimol featurization.

Sterimol has become particularly popular for fitting enantiomeric excess of catalytic reactions, usually expressed as ΔΔ*G* ^‡^ between the diastereomeric transition states. When applied to asymmetric allylation of benzaldehyde and acetophenone, propargylation of acetophenone, and desymmetrization of bisphenols, models that used Sterimol have shown greater correlation compared to those incorporating Charton parameter.^393^ Additional examples include catalytic Michael-like conjugate additions,^394,395^ where regression models employing Sterimol parameters provided more insight into the steric and electronic reaction requirements, leading to better chiral ligand designs through rational modification. In another study,^396^ an MLR for asymmetric C–N dehydrogenative coupling achieved an *R* ^2^ of 0.91 by incorporating Sterimol parameters to account for steric effects, alongside various IR vibrational frequencies to capture other influences. MLR models involving Sterimol metrics, combined with other parameters, such as IR frequencies and absorption intensities, NBO charges, and orbital energies have also been used in practice.^397–403^

Overall, these many applications suggest that Sterimol provides a more accurate measure of steric effects, allowing for better explainability and predictions. Nonetheless, a major limitation of the Sterimol is its reliance on static geometries, neglecting the conformational flexibility of substituents. The recently proposed Boltzmann-weighted modification (wSterimol)^378^ addresses this by incorporating the variability of substituent conformations.

Sterimol is not the only type of steric metric that underwent updates and refinements. The nature of Winstein-Holness A-values^380^ mentioned above (Figure 10B) was recently scrutinized^404^ using DFT simulations. The authors analyzed the relative axial–equatorial conformational stability of substituted cyclohexanes both without and with the Grimme’s D3­(BJ) dispersion correction,^220,222^ which resulted in two electronic energy differences: the purely sterics-based value and that corrected for London dispersion. This led to the creation of two complementary substituent scales either ranked by steric hindrance (always repulsive, *E*_*ster*_ > 0) or by dispersion energy donation (always attractive, *E*_*disp*_ < 0). These contributions compensate each other to an extent, yielding the final electronic energy difference of the conformer equilibrium, which is one major component in the A-value (apart from zero-point, thermal, and solvent corrections). While normally *E*_*ster*_ > *E*_*disp*_, the value of the dispersion contribution can reach >80%, undermining the original idea of the A-value being a purely sterics-based metric. This is especially true for smaller groups that exhibit comparable *E*_*ster*_ and *E*_*disp*_ components. Normalized experimental A-values for a number of functional groups exhibit a complex interrelationship with Sterimol B_1_ parameters (Figure 12A). While some A-values follow a similar trend as B_1_, there is a number of outliers (e.g., F, Cl, CN, SiH_3_, GeMe_3_, SnMe_2_Ph). Importantly, the difference between normalized experimental A-values and B_1_ values for these outliers displays a good linear correlation (*R* ^2^ = 0.8086) with polarizability (Figure 12B), again hinting at the involvement of London dispersion. At the same time, the DFT-computed steric contribution alone shows a good linear correlation with the combined *B*_1_ and *B*_5_ Sterimol parameters (*R* ^2^ = 0.925, cf. Figure 13), thus reconciling the two scales.

**Figure 12:**
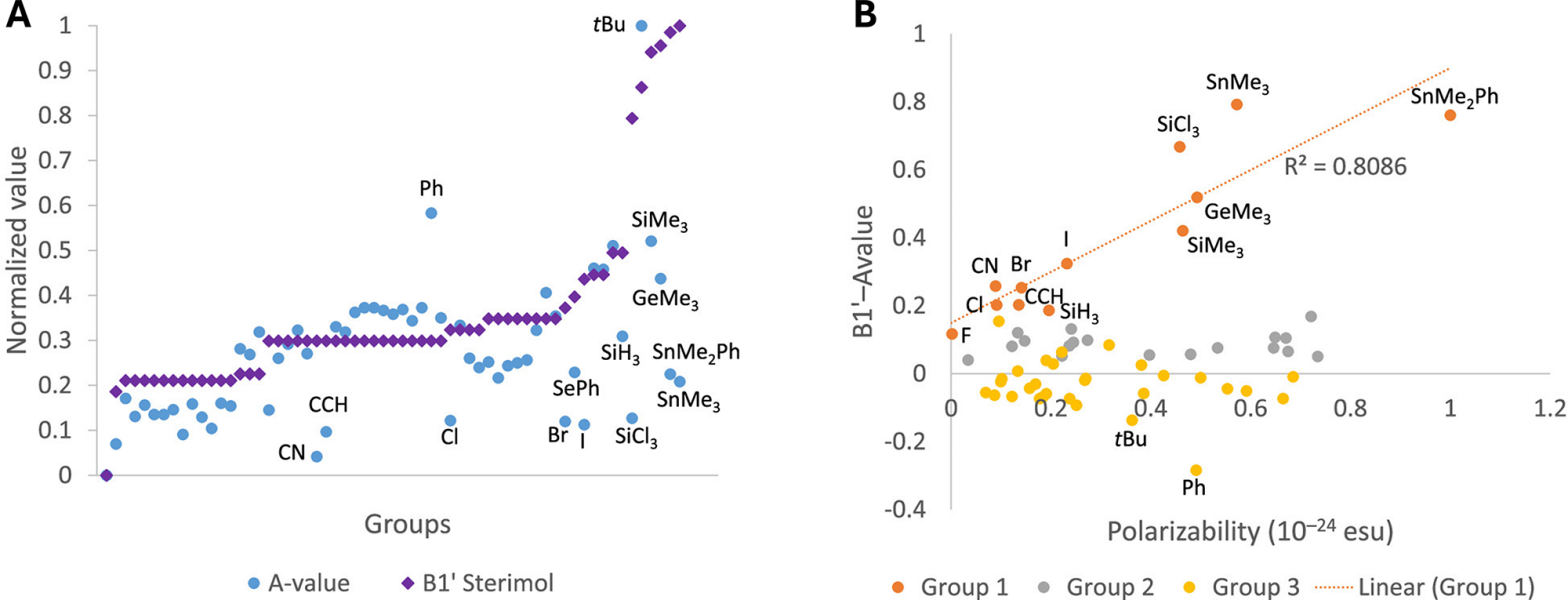
**A**: Comparison of the trends in experimental A-values and the corrected Sterimol parameter B_1_’. **B**: Correlation between the London dispersion component of the A-value and polarizability. Group 1 displays a linear relationship, Groups 2 and 3 have A-values slightly smaller and larger compared to B_1_’, respectively, but no dependence on polarizability. The figure was adapted with permission from the literature.^404^ Copyright 2021 American Chemical Society.

**Figure 13:**
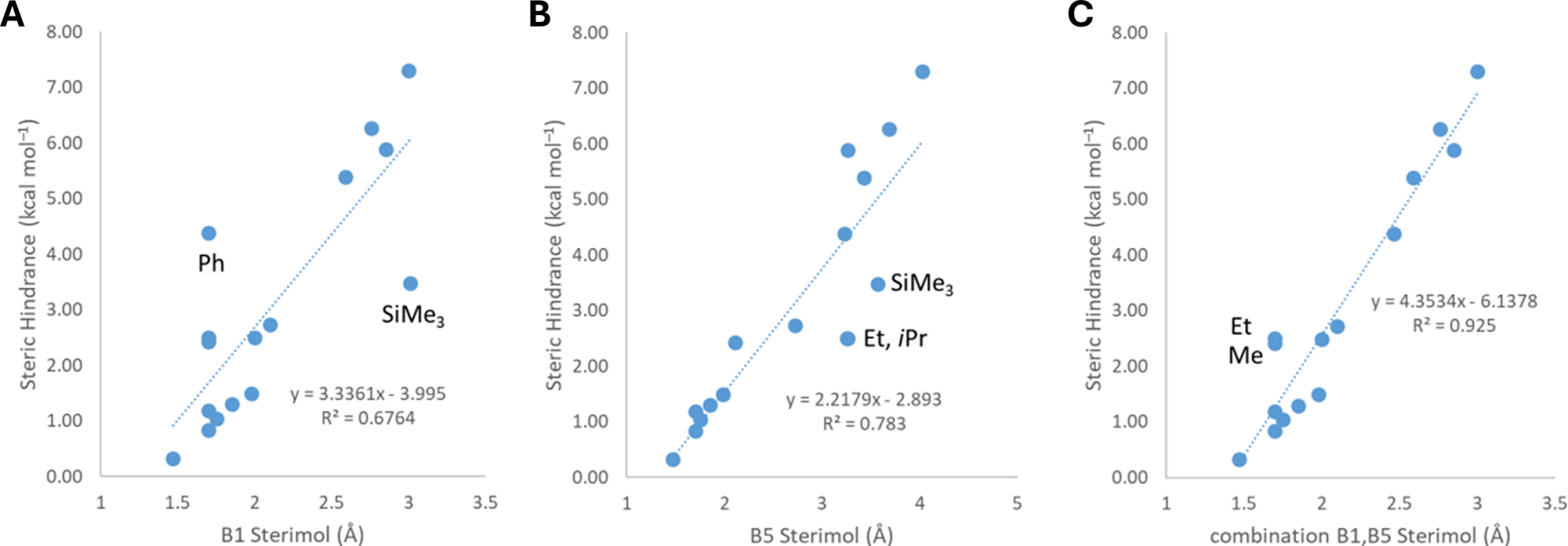
Correlation between the steric part of the computed A-values and the Sterimol parameters B_1_ and B_5_. The figure was adapted with permission from the literature.^404^ Copyright 2021 American Chemical Society.

A more universal way to quantify London dispersion was proposed by *Pollice and Chen*,^405,406^ who postulated a universal quantitative descriptor of dispersion interaction potential, *P*. It is related to the dispersion interaction energy *E*_*disp*_^*i*, *j*^, calculated as a function of heteroatomic dispersion coefficients *C*_*n*_^*i*, *j*^ and the distance between the interacting centers *R*_*i*,*j*_ (Equation 2). The parameter *P* itself can be defined in terms of homoatomic dispersion coefficients *C*_*n*_^*i*, *i*^ (Equation 3). Additionally, the authors introduce the average and maximal values, *P*_*int*_ and *P*_*max*_, over the vdW surface at an electron density isovalue of 0.001 e Bohr^–3^.

$$E_{disp}^{i,j}=-\underset{n}{\sum }\frac{C_{n}^{i,j}}{{R_{i,j}}^{n}}\approx -P_{i}P_{j}$$ 2

$$P_{i}=\underset{n}{\sum }\sqrt{\frac{C_{n}^{i,i}}{{R_{i,j}}^{n}}}$$ 3

The authors demonstrate validity of these descriptors for a number of mono- and polyatomic systems, including, but not limited to, the T-shaped benzene dimer, chalcogen bonds, and an organometallic gold complex. One particular application concerns the enantioselectivity of Pd(0)-catalyzed diarylation of benzyl acrylates (Figure 14).^407^ The original mechanistic study of this reaction utilized the Sigman ^*S*^ *Eπ* metric (*vide supra*) to rank the propensity of the substrate aryl group for π-π interactions with the anthracenyl group of the ligand. A correlation was observed between the ^*S*^ *Eπ* and experimental enantioselectivity for 17 benzyl acrylates (*R* ^2^ = 0.58), supporting the hypothesis that π-stacking does play a role in the stereodefining step. *Pollice and Chen* reanalyzed this dependence, employing either their *P*_*int*_ or the product of *P*_*int*_ and the surface area of the functional group in place of the original ^*S*^ *Eπ*, arriving at models of comparable quality.

**Figure 14:**
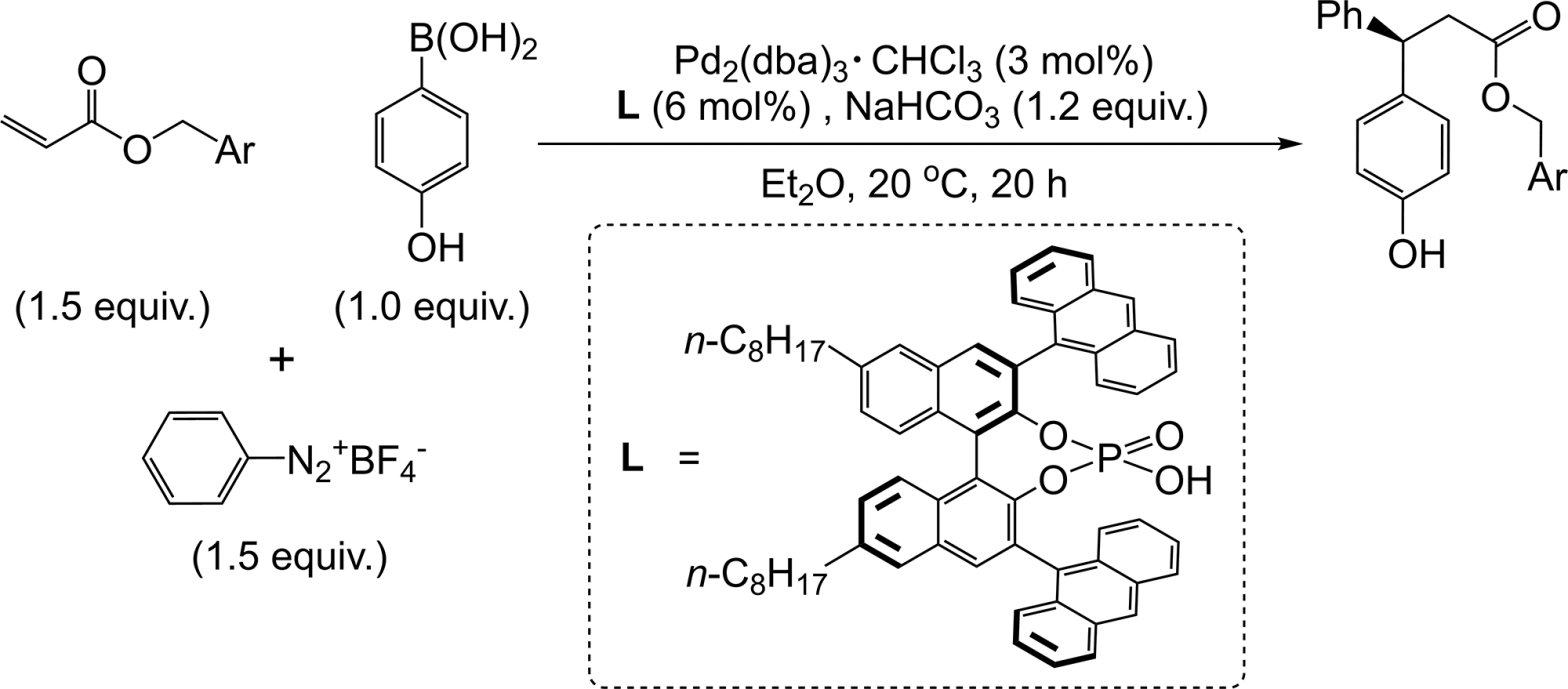
Case study reaction of 1,1-diarylation of benzyl acrylates.^407^

Conceptually very similar dispersion interaction metrics have been introduced soon after this initial demonstration. The so-called van der Waals potential, which was proposed by Lu and Chen in 2020, is conceptually closely related as it also relies on the common atom-pairwise description of dispersion interactions.^408^ However, in contrast to the dispersion interaction potential, *P*, the van der Waals potential does not attempt to separate the influence of the interaction partners, making the absolute value dependent on the probe atom.^408^ Additionally, as implied already by its name, the van der Waals potential does not only account for London dispersion, but also includes an atom-pairwise repulsive term, which is based on the popular Lennard-Jones potential.^66^ In 2025, Kayal et al. combined ideas from both the dispersion interaction potential and the van der Waals potential.^409^ The original ansatz also relied on the dispersion term as used for atom-pairwise dispersion corrections with density functional approximations, with the main difference to the dispersion interaction potential being the inclusion of the functional dependent scaling factor and damping function.^409^ Moreover, just like for the van der Waals potential, the influence of the probe was not attempted to be separated from the interacting molecule.^409^

While dispersion also plays a role in aromatic ion-π interactions, these NCIs are rather dominated by electrostatics and induction. Thus, the quadrupole moment of the arene^410^ or the values of the extrema on the electrostatic potential isodensity surface^411^ can effectively parametrize the interaction energy. This approach can be further enhanced by incorporating the polarizability of the arene to account for induction.^412^ Yet, it has also been demonstrated that orbital electrostatic energy (OEE) may also be a good measure of the electrostatic interaction component.^413^ OEE is defined based on the electron density ρ^*M*^(*r⃗*) of a monomer (i.e., an ion or a π-system), which is the sum over the respective occupied orbitals ∑ _*i*_^*occ*^|ϕ_*i*_^*M*^(*r⃗*)|^2^, and is expressed via four contributions as shown in Equation 4.

$$\begin{array}{c} OEE=-\overset{\pi }{\underset{A}{\sum }}Z_{A}\int \frac{\rho^{ion}\left({\mathrm{r⃗}}^{\prime }\right)}{\left|\overset{⃗}{R_{A}\;}-{\mathrm{r⃗}}^{\prime }\right|}d{\mathrm{r⃗}}^{\prime }-\overset{Ion}{\underset{B}{\sum }}Z_{B}\int \frac{\rho^{\pi }\left(\mathrm{r⃗}\right)}{\left|\overset{⃗}{R_{B}\;}-\mathrm{r⃗}\right|}d\mathrm{r⃗}+\\ +\int \int \frac{\rho^{ion}\left({\mathrm{r⃗}}^{\prime }\right)\rho^{\pi }\left(\mathrm{r⃗}\right)}{\left|\mathrm{r⃗}-{\mathrm{r⃗}}^{\prime }\right|}d\mathrm{r⃗}d{\mathrm{r⃗}}^{\prime }+\overset{\pi }{\underset{A}{\sum }}\overset{Ion}{\underset{B}{\sum }}\frac{Z_{A}Z_{B}}{\left|\overset{⃗}{R_{A}\;}-\overset{⃗}{R_{B}\;}\right|}, \end{array}$$ 4

In this equation, *A* and *B* refer to the corresponding arene-π system and the ion, respectively, while *Z*_*A*_, *Z*_*B*_, *R*_*A*_ and *R*_*B*_ represent charges and positions of the corresponding nuclei. The first two semiclassical terms in Equation 4 capture the electrostatic attraction between the arene and the ion (and *vice versa*), whereas the other two terms account for interelectronic and internuclear Coulomb repulsion. Thus, OEE considers both the nature of the arene and the ion, unlike the arene-only metrics mentioned above. In the original work, which focuses on substituted six-membered arenes and small ions, both inorganic and organic, the authors show that while anion-π interaction energies can be effectively parametrized using OEE alone, cation-π interactions are best captured by an MLR model that combines OEE and polarizability. These findings underscore the critical role of polarization effects in cation-π interactions.

Given the pivotal role of electrostatic interactions in halogen bonding,^414–416^ it appears intuitive to characterize it through a metric tied to the electrostatic potential, such as atomic charges. However, since there is no unique rigorous scheme for measuring atomic charges in molecules, the multiple methods that have been proposed for doing so often yield inconsistent results,^417,418^ each inherently assuming a spherical charge distribution around atoms. This simplification is frequently inaccurate, emphasizing the need for a more physically grounded metric. Typically, one defines the maximum electrostatic potential value, *V*_*max*_, which corresponds to the center of a σ-hole on the isodensity surface of the halogen atom involved in the bond (most commonly using an isovalue of 0.001 au). While this atom is often referred to as the ”σ-hole donor”, it is worth noting that, from an electron-centered perspective, one might intuitively consider it a halogen bond acceptor. For a given electron donor, *V*_*max*_ was found to linearly correlate with the complexation energy with typical *R* ^2^ values above 0.95.^106,419–421^ This correlation can be further generalized for different electron donors by including the most negative electrostatic potential on the binding site of σ-hole acceptors, *V*_*min*_. Multivariate linear models that include both *V*_*max*_ and *V*_*min*_ as separate features, or the product *V*_*max*_ · *V*_*min*_, were able to achieve *R* ^2^ values above 0.90.^106^ Recently, more complex ML models have been developed to predict halogen bonding energy, either directly or indirectly through *V*_*max*_ predictions.^422,423^ These approaches will be discussed in more detail in the section about predicting NCIs with ML.

Aside from tailor-made metrics for featurizing the propensity for specific types of NCIs, data-driven approaches can be used to identify more general parameters that exhibit a strong correlation with the interactions of interest. One such study involves an analysis of enantioselective reactions catalyzed by chiral pyrrolidine-based hydrogen-bond donors,^400^ where the transition states are stabilized by attractive NCIs. The authors developed dedicated MLR models (*R* ^2^ = 0.90 for validation and 0.79 for prediction), where the NBO charge of the N–H hydrogen bond donor group had one of the highest absolute weights. Thus, it has been suggested that the corresponding group is involved in hydrogen bonding and thus contributes to the enantiomeric excess, which was expressed as ΔΔ*G* ^‡^.

### 5.3 Global Descriptors

Thus, far, our exploration of the features used to represent NCIs has progressed from the most granular level, where each individual interaction is distinctly characterized, to more condensed parameters that summarize all interactions of a given type under a unified metric, without detailing the specific components. Next, we introduce the highest level of abstraction in the description of NCIs. This level aims to capture the cumulative effects of various interactions combined, distilling complex molecular behaviors into global descriptors that remain both powerful and predictive, allowing for greater generalization across diverse chemical systems.

One such descriptor is the DFT electronic energy, which was used in an ML approach^424^ to introduce NCI corrections to DFT calculations across several GGA and hybrid functionals (M06–2X, ωB97X-D, B3LYP, and PBE), with or without dispersion corrections and implicit solvation. The model of choice was a Generalized Regression Neural Network (GRNN), trained on a data set derived from established benchmarks at the CCSD­(T)/CBS level of theory, namely S22,^311,425^ S66,^309,426^ and X40.^320^ Molecules from these benchmarks could be categorized into four types based on the corresponding dominant NCI, specifically dispersion, hydrogen bonding, halogen interactions, and mixed complexes.

Partial Least Squares (PLS) analysis revealed that by far the major feature that defines CCSD­(T)/CBS-level NCI reference energies was the DFT NCI energy. Further analysis of 43 DFT-derived and structural descriptors, followed by GRNN refinements, identified another universal feature – the number of valence electrons. Two pairs of additional features were also shown to be important, with either pair being relevant depending on the functional used. These pairs were found to be 1) the arrangement of the monomers and the electronic spatial extent; 2) the dipole moment and the energy of the second lowest unoccupied molecular orbital (*E*_*LUMO*+1_). The number of valence electrons was consistently selected across all functionals, indicating the electronic nature of NCIs. The dipole–dipole interactions affect charge transfer, electronic transitions, and excited-state properties, all influencing NCIs. The arrangement of the monomers impacts the number of close contacts between them, directly affecting the interaction strength. The electronic spatial extent, which characterizes the distribution of the electron density, correlates with the molecular interaction region. However, the relevance of *E*_*LUMO*+1_ in NCI predictions was less clear. The authors suggest that its selection might have been a numerical coincidence, as other principal components with slightly lower but comparable magnitude could have been chosen as well. Overall, the GRNN-based correction significantly improved the accuracy of DFT in predicting NCIs compared to the CCSD­(T)/CBS benchmark values. RMSEs were reduced from 1.43–3.98 kcal mol^–1^ for uncorrected DFT to 0.46–0.62 kcal mol^–1^ for corrected DFT, and Mean Absolute Errors (MAEs) decreased from 1.00–3.01 kcal mol^–1^ to 0.33–0.46 kcal mol^–1^ after applying the ML correction, achieving what is often referred to as chemical accuracy. The smallest RMSEs of less than 1.5 kcal mol^–1^ were achieved in protein-like environments, which offer a good balance between the understabilization of NCI complexes in water and their overstabilization in vacuum.

Another study^427^ with a similar goal – providing NCI corrections to DFT energies – used kernel ridge regression (KRR) along with DFT energies and topological descriptors. The authors targeted correcting the B3LYP-D3, PBE, PBE-D3, and M06–2X levels of theory. While three of these methods already account for dispersion, they still exhibit deviations from the CCSD­(T)/CBS reference energies taken from the S66×8 ^309^ data set (*vide supra*).

For the systems analyzed, the authors derived promolecular electron densities (i.e., the sum of the densities of noninteracting atoms), which did not require costly self-consistent field calculations and could readily be expressed in analytical form, and thus easily differentiated and integrated. Despite disregarding chemical bond formation, promolecular densities are known to contain some information about the would-be chemical bonds.^428^ The authors of this work took this approach one step further by looking at the promolecular densities of NCI-bound complexes, targeting the regions of low, yet structured, electron density at the interface between two interacting monomers. The said structure manifested as a low reduced density gradient, where the density distribution at the periphery of the nuclei followed a dependence other than exponential decay.^429^ These regions were then classified based on the magnitude of ρ and the sign of the second eigenvalue of the electron density Hessian matrix, sign­(λ_2_): a negative sign­(λ_2_) suggested an attractive interaction, while a positive value indicated repulsion. The regions were further categorized into three groups according to the value of ρ·sign­(λ_2_), namely attractive (−0.1 to – 0.02 au), vdW interactions (−0.02 to +0.02 au), and repulsive (+0.02 to +0.1 au). The descriptors derived from integrating ρ in these promolecular regions were then used alongside the DFT energy in the KRR. In all cases, the correction yielded better results than the baseline DFT methods, with or without accounting for the basis set superposition error. To exemplify the results, the mean absolute percentage error (MAPE) computed for 159 NCI complexes was reduced from 57% for uncorrected PBE/6–31+G­(d,p) to 17% for the KRR-corrected method. The same applies to the dispersion-corrected B3LYP-D3/6–31+G­(d,p) level of theory with 17% and 6% before and after the KRR correction, respectively.

Subsequently, the same research group^430^ expanded this approach to study larger systems. They focused on a homodimer of the peptide NYNYN, which consisted of asparagine (N) and tyrosine (Y) residues. These amino acids introduced polar groups and aromatic rings, providing a compact system for examining a wide variety of NCIs. Each monomer was modified by acetylation on the *N*-terminus and amidation on the *C*-terminus, resulting in an overall neutral structure. A 10 ns classical MD simulation was performed, selecting 1000 evenly spaced frames, for which reference energies were computed using the BSSE-corrected PBE-D3/6–31G+(d,p) level of theory. Ridge regression on a data set of 700 structures, validated using 5-fold cross-validation, yielded an average prediction error of 9%, with a training score of 0.62 and a test score of 0.59.

These studies provide a proof of concept that DFT interaction energies can be effectively corrected using topological descriptors derived from the promolecular electron density at the monomer interface, with minimal computational cost at that. Interestingly, while these descriptors are based on promolecular densities, they seem to capture information beyond the capabilities of DFT. Furthermore, due to the simplicity of obtaining promolecular densities, this method shows great potential in terms of scalability. In later work, the same research group^431^ enhanced these topological descriptors by redefining them based on QM densities derived from extremely localized molecular orbitals (ELMOs) rather than promolecular densities, the former of which are generally faster to compute than traditional, delocalized MOs. This led to an improved correlation coefficient with the reference NCI energies in the S66 benchmark data set,^309,426^ increasing from 0.958 obtained via promolecular densities to 0.981 when using densities from ELMO/6–31G­(d,p).

## 6 Predicting Noncovalent Interactions

Having established the key methods for featurizing NCIs, we now turn our focus to the application of ML techniques for predicting these interactions. ML models, when properly trained, offer the ability to predict the occurrence, strength, and influence of NCIs with remarkable accuracy. In this section, we explore a range of methods from traditional regression models to advanced neural networks, from purely data-driven approaches to the frameworks that aim to capture the underlying physics. We also discuss the types of data sets employed and the predictive performance of these models across diverse chemical subspaces.

### 6.1 Data-Driven models

We start our discussion with data-driven approaches that do not always capture the physical mechanisms underlying NCIs but rather focus on learning patterns from large data sets to reproduce the overall distributions of the data. While they may not explicitly incorporate physical principles like electrostatics or vdW forces, these models are still capable of accurately predicting the behavior and strength of NCIs by identifying and generalizing from correlations in the data.

One key advantage of data-driven models is their simplicity. They bypass the need for detailed knowledge of the interaction physics, making them easier to implement and scale across diverse chemical systems. This simplicity, coupled with flexibility, allows for efficient training and prediction across large data sets. Additionally, with the right data set, these models can extrapolate beyond the training data, at least to some extent, rendering them useful in scenarios where capturing complex interactions through analytical methods would be cumbersome or computationally expensive. However, the absence of explicit physical constraints in data-driven models can also lead to significant limitations. Without understanding the underlying forces, these models may sometimes produce unphysical results, especially when applied far beyond the scope of the training set. While their flexibility and simplicity are valuable assets, careful validation is necessary to ensure that the predictions remain reliable, particularly in novel or less-explored chemical spaces.

One such model is DelFTA,^314^ which corrects GFN2-xTB semiempirical energies of drug-like molecules to the ωB97X-D/def2-SVP level of theory, increasing accuracy for NCIs in the process. The model uses 3D-coordinates as inputs and employs a E(3)-invariant three-dimensional MPNN with harmonically encoded interatomic distances and five message-passing layers, followed by an additional network that would derive the final target values, i.e., HOMO and LUMO energies, HOMO–LUMO gaps, dipole moments, Mulliken charges, Wiberg bond indices, and conformer pairwise energy differences. DelFTa exploits the Δ-learning approach. It is not trying to predict the DFT-derived properties directly but rather a differential responsible for the loss of accuracy at the semiempirical level. The model was trained on the QMugs data set,^294^ a rigorously curated data set comprising over 665,000 biologically and pharmacologically relevant molecules from ChEMBL^312^ (release 27), which was expanded into approximately 2 million conformers (*vide supra*). While DelFTa does not explicitly target NCIs, QMugs monomers feature intramolecular NCIs. This allowed DelFTA to successfully extrapolate to other systems, predicting both intra- and intermolecular interactions in biomolecules such as weak and strong hydrogen bonds in α-helices, RNA base pairs, β-turns/sheets, and the glutamate binding pocket (Figure 15). However, the authors observed decreased performance for interactions involving charged species such as hydrogen bonding with phosphate groups, presumably because the model was trained on uncharged molecules.

**Figure 15:**
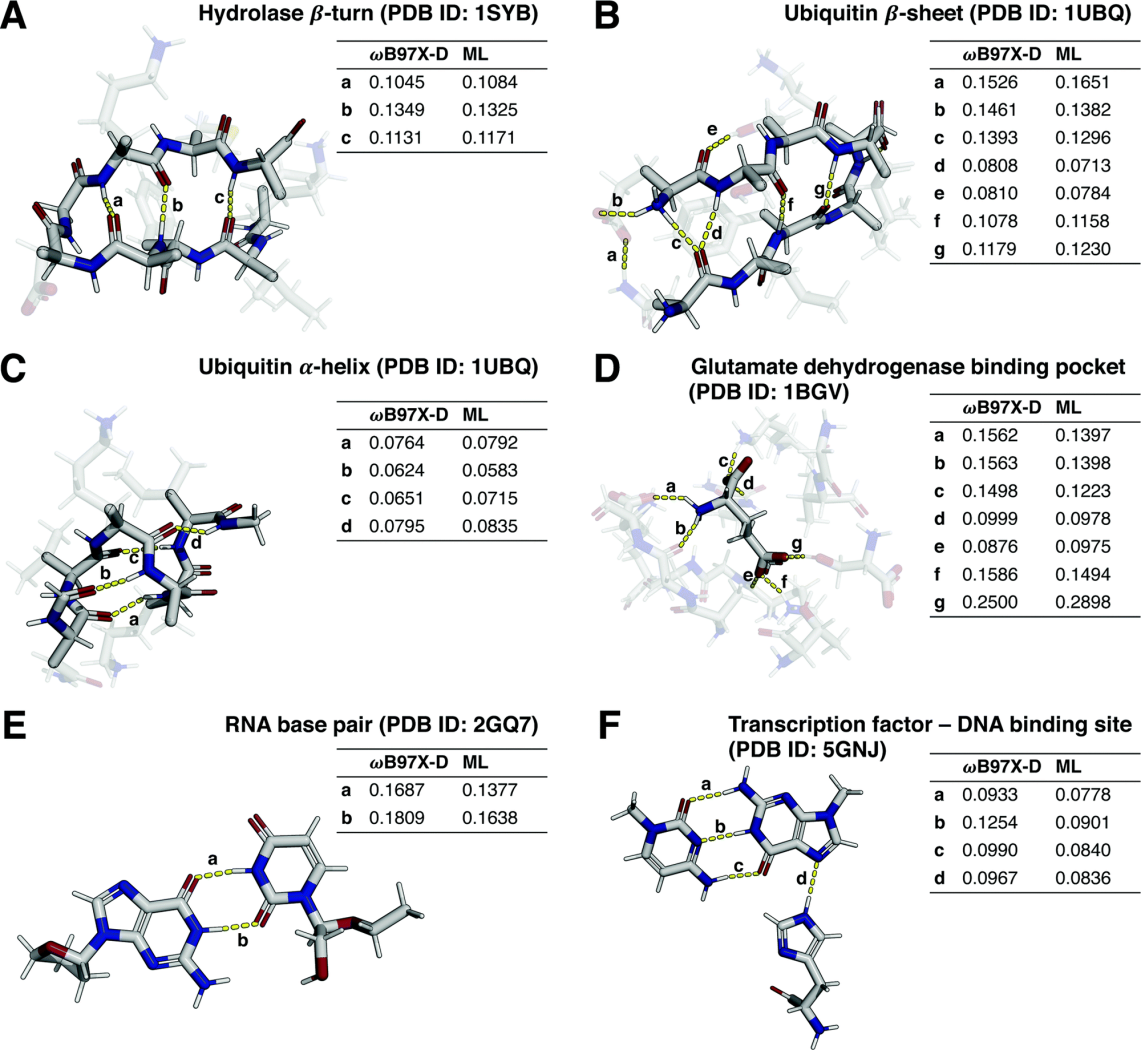
Wiberg bond orders for NCIs in selected biomacromolecules calculated at the ωB97X-D/def2-SVP level of theory and predicted by DelFTa (denoted as ML).^314^ Only interactions with Wiberg bond orders within 0.05–0.8 are shown. H atoms are shown in white, C atoms in gray, O atoms in red, and N atoms in blue. The figure was adapted with permission from the literature.^314^ Copyright 2022 Royal Society of Chemistry.

VmaxPred^422^ is a regression model based on the SVM architecture, designed to predict electrostatic potential at the center of a σ-hole (*V*_*max*_) on a halogen atom in halogenated five- or six-membered aromatic rings, including fused systems. The predicted value can then be used to gauge the halogen bonding energy as *V*_*max*_ is widely regarded as a standard descriptor for halogen bonding strength.^106,419–421^ Calculating *V*_*max*_ with quantum-mechanical methods at a reasonable level of theory is time-intensive, limiting its practicality for large compound libraries in drug discovery. VmaxPred was developed to mitigate this issue by speeding up the computations by 5 to 6 orders of magnitude compared to the MP2 level of theory. The model outputs the most positive electrostatic potential on the halogen atom isodensity surfaces at both 0.001 au and 0.02 au, which represents the σ-hole. These two isodensity values were specifically chosen because the former typically corresponds to the distance at the onset of the halogen bond formation, while the latter provides an intrinsic measure of the halogen bonding potential without any influence of the neighboring groups. It was noticed that the value of *V*_*max*_, while significantly increasing from 0.001 au to 0.02 au, does so disproportionally for different systems, which eventually led to models of different quality.

The SVM model of VmaxPred uses a feature vector (Figure 16) that includes information about both positive and negative charges in the molecule, and divides the molecule into spheres of atoms, each *N* = 2, 3, 4, ... bonds away from the interacting halogen. The vector contains both electronegativity and the lone-pair electron index (a measure of electrostatic interactions)^432^ for both the halogen and for the substituents in each sphere. Additionally, the halogen is characterized by electrotopological state indices (metrics for electronic environment and molecular connectivity)^433^ of itself and its connected carbon atom. Finally, each sphere of the aromatic ring is represented by both the summed electronegativities of the constituent atoms and their lone-pair electrons index.

**Figure 16:**
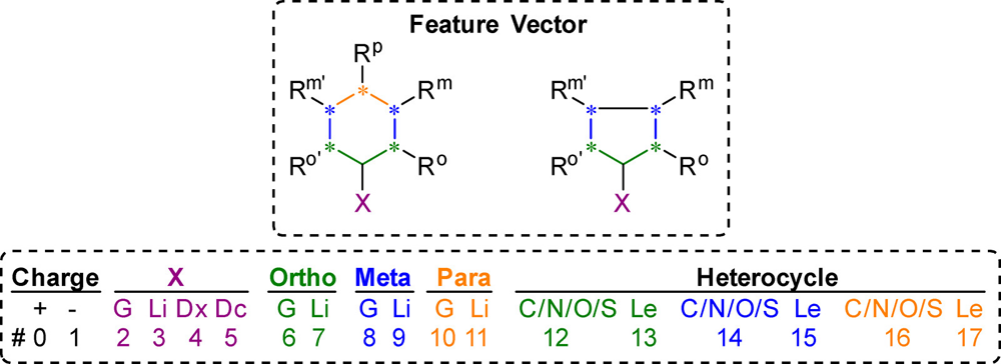
Feature vector in the VmaxPred model.^422^ + and – : the metrics for positive and negative charges;^422^ G: group electronegativity; Li: lone-pair electrostatic interaction measure;^432^ Dx, Dc: electrotopological state indices of the halogen (X) and its bound carbon atom (C);^433^ C/N/O/S: total Pauling electronegativity of atoms within the respective sphere; Le: lone-pair electron index. The figure was adapted with permission from the literature.^422^ Copyright 2019 American Chemical Society.

Initial training and hyperparameter optimization were conducted using two data sets. Data set 1 (training) included 3,243 uncharged and 162 charged benzene derivatives, along with 16,323 substituted heterocycles, with equal numbers of chlorine, bromine, and iodine atoms. Data set 2 (validation) consisted of 100 structures randomly selected from an in-house library of approximately 300,000 purchasable fragments (based on Aldrich Market Select) and 400 structures from the Protein Data Bank (PDB, downloaded on 01–14–2015), each containing up to 22 heavy atoms and at least one heavy halogen. The final training and validation were carried out on the combined data set from both 1 and 2, complemented by data set 3 with 586 molecules from an in-house, diversity-optimized, halogen-enriched fragment library,^434^ which includes all different protonation states and tautomeric forms. A standardized preparation process was applied to all 20,814 structures, involving MP2/def2-TZVPP geometry optimization, coordinate transformation for visualization, electrostatic potential grid calculation, and *V*_*max*_ determination.

Final training based on the 0.02 au isosurface with 10-fold cross-validation resulted in a correlation coefficient of 0.9778 with an RMSE of 0.0081 au. For the model trained on the 0.001 au surface, a correlation coefficient of 0.9647 with an RMSE of 0.0061 au was obtained. This further translated into complexation energy metrics (using *N*-methylacetamide as a donor) of *R* ^2^ = 0.928 and RMSE = 0.659 kcal mol^–1^ for 0.02 au, and *R* ^2^ = 0.875 and RMSE = 0.868 kcal mol^–1^ for the 0.001 au isosurface.

The study of Devore and Shuford^423^ tackles a similar challenge as VmaxPred – the prediction of the maximum point on the electrostatic potential surface (*V*_*max*_) for a halogen X···NH_3_ bond along with the corresponding bond energy (*E*_*bind*_) and local force constant (*k*_*X*···*N*_^*a*^). The study focuses on DFT-calculated halogen-bound complexes (1,210 in total) of symmetrically substituted halobenzenes and 1-aryl-2-haloacetylenes (halogen = Cl, Br, I, At) as σ-hole donors. It employs three ML models: RF, XGB, and SVM.

In their study, the authors focus on two main tasks: classification for identifying the interacting halogen atom, when several are present in the molecule, and regression to derive the target properties. For classification, they begin by identifying the 5 most important features using recursive feature elimination (RFE) as implemented in the scikit-learn Python package.^435^ The results demonstrated that the set of relevant features depends on the structure of the σ-hole donor. For substituted halobenzenes in particular, the most effective features were primarily related to the energetics of the halogen-bound complex, namely the complex binding energy (*E*_*bind*_) and formation energies of the bound partners (*E*_*def*_^*NH*_3_^ and *E*_*def*_^σ–*donor*^). These were followed by the C–X bond length in the complex and the magnitude of charge transfer from ammonia to the σ-hole donor (Δρ). Contrarily, the important features for the 1-aryl-2-haloacetylene complexes were more related to the electronics at the X···NH_3_ bond critical point, specifically the Laplacian of the electron density (∇ρ^*BCP*^), the sign of the second eigenvalue of the electron density matrix multiplied by the electron density (*sign*(λ_2_) · ρ), and the total energy density (*H*(*r*)^*BCP*^). The fourth and fifth most important features were the complex binding energy (*E*_*bind*_) and formation energy of the σ-hole donor (*E*_*def*_^σ–*donor*^). Feature identification was followed by the use of RF, XGB, and SVM to predict the interacting halogen. For both types of σ-hole donors, the prediction accuracy of the RF and XGB models was approaching 100%, as confirmed by 5-fold cross-validation. The SVM models, however, were underperforming, showing only 96.69% prediction accuracy for substituted halobenzenes and 87.7% for 1-aryl-2-haloacetylenes.

The regression tasks were implemented using two alternative types of descriptors: either Morgan molecular fingerprints^436,437^ or features identified via the same type of RFE used for classification. For Morgan fingerprint-based models, the binding energies of complexes with ammonia show MAE and RMSE values ranging between 0.5–1.0 kcal mol^–1^ (compared to a typical bonding energy within −20 kcal mol^–1^). For the X···NH_3_ local force constant, MAE and RMSE fall between 0.005–0.022 mDyn/Å (with typical force constants within 0.3 mDyn/Å). These results are consistent across all the three ML models tested. However, RMSE and MAE for *V*_*max*_ are quite large across all ML models, ranging within 2.20–7.39 kcal mol^–1^ (with typical *V*_*max*_ values up to 70 kcal mol^–1^). The authors attribute this to the high sensitivity of *V*_*max*_ with respect to the electronic environment, which can be altered in the process of conformational changes that are not captured in the features.

Compared to fingerprint-based learning, the descriptor-based prediction performs considerably better. The majority of RFE-selected features are primarily found in the halogen-bound complex, and very few are based on either of the monomers alone. When predicting *V*_*max*_, the energetic features (*E*_*bind*_, *E*_*def*_^*NH*_3_^ and *E*_*def*_^σ–*donor*^) were found to have the largest prevalence with the electronic ones (especially electron and energy density at the bond critical point of X···NH_3_) having comparable importance. Similar trends can be seen when predicting the *E*_*bind*_ or *k*_*X*···*N*_^*a*^. Interestingly, in this case, there was a difference in predictive performance for *V*_*max*_ between the models. RF and XGB gave MAE within 0.56–1.15 kcal mol^–1^ and RMSE within 0.88–1.69 kcal mol^–1^, SVM performed considerably worse with MAE within 2.60–3.66 kcal mol^–1^ and RMSE within 3.28–4.51 kcal mol^–1^. Interestingly, when evaluated on a data set separated by the type of interacting halogen, MAE and RMSE of SVM improve to 0.64–2.10 kcal mol^–1^ and 0.92–2.60 kcal mol^–1^, respectively. The authors suggest that the SVM algorithm may have difficulty separating densely packed data points and performs more effectively with sparse data sets, while the RF and XGB algorithms may be better equipped to distinguish groups or trends within tightly clustered data. Same trends are observed for *E*_*bind*_ and, to a lesser extent, *k*_*X*···*N*_^*a*^. For *E*_*bind*_, RF and XGB give MAE within 0.05–0.12 kcal mol^–1^ and RMSE within 0.10–0.17 kcal mol^–1^. For *k*_*X*···*N*_^*a*^, the corresponding values are 0.001–0.002 mDyn/Å and 0.001–0.004 mDyn/Å, respectively. SVM on undivided data set provides error metrics that are typically 5–10 times higher. Even after using the divided data sets, these values remain up to several times higher compared to the other methods.

Overall, this study is a proof of concept that ML models can effectively predict key properties of halogen bonds, such as *V*_*max*_, *E*_*bind*_ and *k*_*X*···*N*_^*a*^. By leveraging different feature sets and ML algorithms, the study shows how models like RF and XGB can achieve high accuracy in both classification and regression tasks, paving the way for more efficient predictions of halogen bonding properties in various chemical environments.

The work of Tabet et al.^438^ deals with kernelized SVR-based prediction of equilibrium binding constants for 1:1 host–guest complexes of small drug-like molecules with cucurbit[7]­uril (CB[7]; Figure 17). CB[7] is a water-soluble, macrocyclic receptor that binds guest molecules through hydrophobic interactions within its cavity and polar interactions with its carbonyl oxygens at the periphery. Binding with CB[7] modifies drug release and pharmacokinetic profiles by reducing active concentration, thereby increasing the half-life of pharmaceutically active compounds *in vivo*. Consequently, predicting binding affinity with CB[7] could be a valuable tool for drug development.

**Figure 17:**
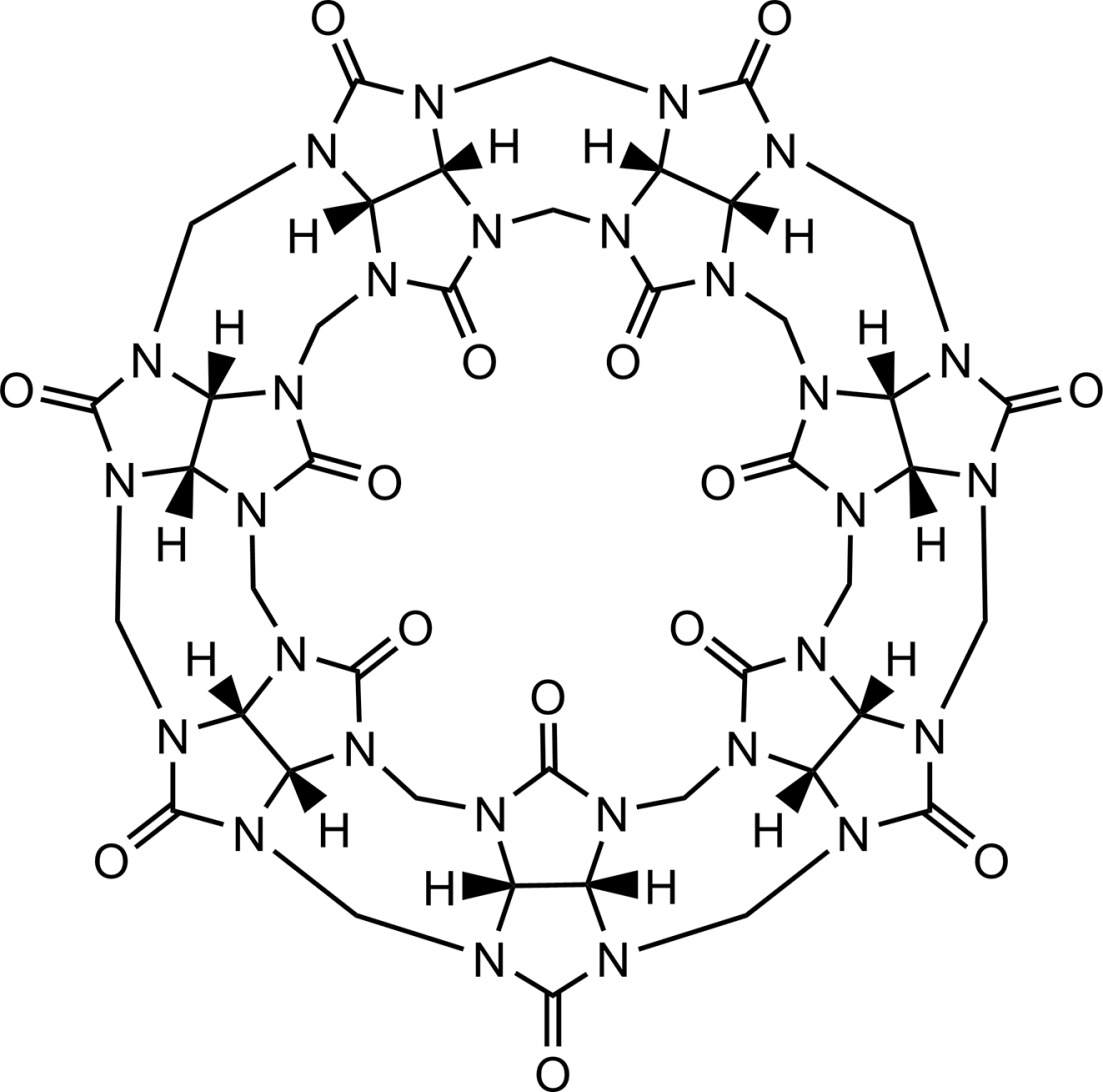
Structure of cucurbit[7]­uril.

The training set contained 194 experimental binding coefficients for 146 complexes (some constants were measured several times in different experimental conditions). Additionally, in order not to skew the model with extreme values, the authors included 3 verified nonbinding molecules, whose affinity was set to zero. The SVR model used 17 features in total, both computational and experimental. For computational features (geometry, electrostatic properties, electric field gradient, etc.), all complex geometries were optimized at the B3LYP/6–311++G** level of theory *in vacuo*. Experimental features were drawn from the literature and included pH, temperature, counterion, and salt concentration, among others. This led to a relatively small but high-dimensional data set.

After rigorous assessment of the kernelized SVR model, hyperparameter optimization, and leave-one-out cross-validation, the best trained model had an MAE of 1.6266 and an *R* ^2^ of 0.3820. This was sufficient to predict the binding affinity of two promising drugs for pediatric low-grade gliomas, one of which was estimated by the model to statically bind, while the other to exhibit no affinity. Both predictions were experimentally confirmed by the authors. This study shows that ML may prove valuable in drug development, and the opportunities will continue to grow as new data sets are developed and refined.

Among the data-driven models that are also capable of predicting NCIs are TFRegNCI and TFViTNCI.^439^ Their original purpose is to provide NCI-corrections to DFT-calculated energies. However, owing to Gradient-weighted Regression Activation Mapping (an adapted version of Gradient-weighted Class Activation Mapping, Grad-CAM^440^), the model is capable of locating the regions of important input features within the electron density, i.e., the very regions related to the emergence of NCIs.

Both TFRegNCI and TFViTNCI are built on the previous work from the same authors^441^ and employ a multimodal approach that incorporates both electron density and QM descriptors as features. In each model, 3D electron density features are initially extracted from the input density cube file, using either the CNN-based RegNet architecture^442^ (in TFRegNCI) or Vision Transformer (in TFViTNCI).^443^ To reduce computational time, the models can also use a 2D pattern input derived from transformed 3D electron densities, achieving comparable prediction accuracy. In either case, the feature extraction is followed by a transformer-based encoder that merges the feature embeddings from the electron density with chemical property descriptors, ultimately producing the final energy corrections. The training data set was assembled from four benchmarks: the original S22×5,^425^ S66×8,^309^ and X40×10 ^320,321^ data sets (covering molecular systems dominated by hydrogen bonding, dispersion, and halogen interactions, together with mixed interactions), along with a subset from the dispersion-dominated D442×10 benchmark,^281^ selecting 199 complexes, each represented by 3 points along the dissociation curve. Electron densities were calculated at the M06–2X/6–31G* level of theory *in vacuo*. On a test set, both TFRegNCI and TFViTNCI, with and without transformed 2D electron density, showed RMSEs within 0.15–0.18 kcal mol^–1^ and MAEs within 0.10–0.14 kcal mol^–1^, with TFRegNCI-2D providing the best results at 0.15 kcal mol^–1^ and 0.10 kcal mol^–1^ for RMSE and MAE, respectively.

For the purpose of this review, TFRegNCI and TFViTNCI are notable for their visual interpretability that is enabled by the Grad-CAM module. Generally, the TFRegNCI model that uses 3D electron density for feature selection (TFRegNCI-3D) demonstrated a more chemically meaningful and less noisy electronic density feature subspace compared to TFViTNCI-3D. This aligns with the higher general predictive power of TFRegNCI-3D compared to TFViTNCI-3D as mentioned above. Figure 18 shows the feature selection density subspaces obtained from TFRegNCI-3D alongside the most relevant molecular orbitals and NCIPLOT isosurfaces, where interactions are identified via the reduced density gradient analysis.^429,430^ The comparison includes four complexes, each dominated by a distinct interaction type, that is hydrogen bonding, dispersion, halogen bonding, and mixed interactions. For all but the dispersion-dominated case, the feature subspace generally aligns with either the NCIPLOT or the HOMO isosurface. Dispersion poses a larger challenge, partly because orbital involvement is very nuanced. Overall, this work marks an important step toward interpretable ML predictions for NCIs, showing that even data-driven methods can capture aspects of the underlying physical reality. While further research is needed to enhance the practical application of models like TFRegNCI and TFViTNCI, they serve as an impressive conceptual foundation.

**Figure 18:**
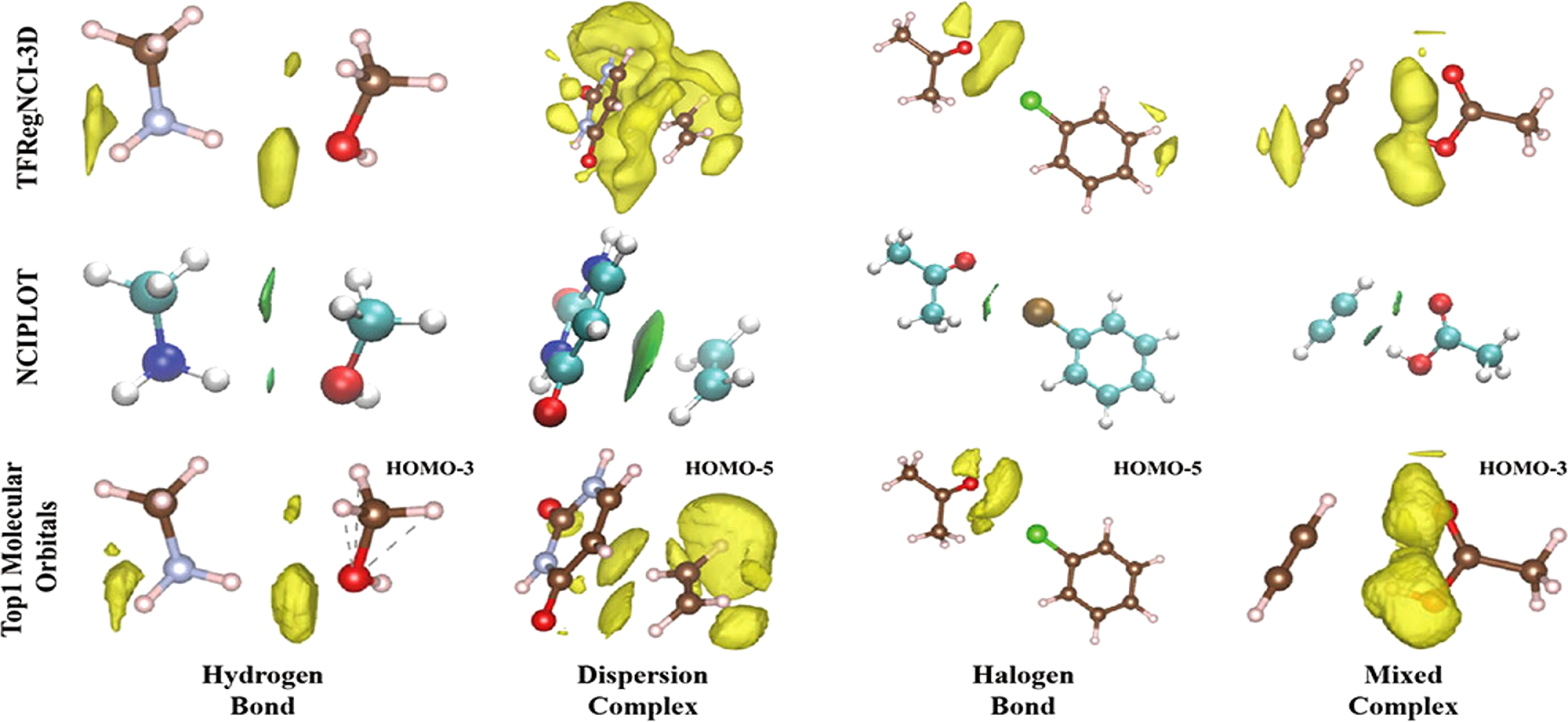
Feature visualization of TFRegNCI-3D compared to the NCIPLOT method and relevant MO isosurfaces.^439^ The figure was adapted with permission from the literature.^439^ Copyright 2023 American Chemical Society.

### 6.2 Physics-Informed Models

Lately, there has been a rise in popularity of physics-informed ML models that respect the physical nature of NCIs. They partition the interaction energy in a SAPT-like^291^ manner, isolating the electrostatic, induction, dispersion, and exchange components or alike. Imposing physical constraints offers a few advantages. First, it enhances interpretability as each energy component can be analyzed individually, rather than accepting a black-box result. Second, it improves generalization and transferability, as the partitioning imposes meaningful constraints while maintaining sufficient flexibility for reliable extrapolation. Third, these constraints lead to higher accuracy and prevent unphysical behavior by adhering to well-defined interaction forms. Finally, physics-informed models often require less training data, as the built-in constraints allow the model to learn more efficiently.

A common trait of such models is the atom-pairwise treatment of the NCI energy components as opposed to representing individual atomic contributions that some ML models^444^ employ under the assumption that such a partition exists and is learnable. As was noted previously,^445^ no uniatomic *ab initio* energy labels can be derived, priming such models for poor generalization, limited transferability, and, as a consequence, pathological PES. In contrast, treating NCIs as more physically meaningful atom-pairwise terms is a common practice in many popular force fields^446–448^ and in several empirical dispersion corrections such as the family of corrections developed by the Grimme group.^220^ Below, we discuss some of the physics-based ML models that predict NCIs in an atom pairwise fashion, while respecting the SAPT-like energy partitioning, namely AP-Net,^445^ CONI-Net,^449^ IPML,^450^ and CLIFF.^451^

AP-Net^445^ is an FNN potential for predicting meaningful contributions to the NCI energies within the framework of the SAPT partitioning. The authors propose an atom pair symmetry function (APSF) that they use as a feature in the model. APSF encodes the environment of the atom pair in a form that is invariant to rotations, translations, and permutations, thus ensuring that the physical properties of the system are preserved regardless of orientation. Importantly, APSF does not have a sharp distance cutoff, unlike some ML-modeled atomic environments that are limited to distances as short as 5 Å, which contradicts the very nature of NCIs, failing to fully capture the long-range electrostatic and, to a lesser degree, vdW interactions.

The model was trained on complexes of *N*-methylacetamide paired with 92 different small hydrogen-bond donors and acceptors with varying monomer separations and orientations, resulting in 7784 distinct configurations. Additionally, the training data were augmented with 2192 neutral dimers from the side chain–side chain interaction subset of the BioFragment database (SSI BFDb).^306^ The test set included hydrogen-bound complexes of *N*-methylacetamide and isoquinolone. AP-Net demonstrates remarkable transferability. After being trained on a data set without the H_2_O dimer, it still accurately predicts the smooth PES for this dimer, capturing both the radial and angular dependence of the hydrogen bond. Additionally, despite being trained on the hydrogen bonding-enriched data set, AP-Net generalizes well across the chemical space, achieving an MAE of 1.1 kcal mol^–1^ on the diverse S66×8 ^309^ NCI benchmark. This suggests that the model learns physically meaningful relationships with interpretable pairwise interaction energies, further enhancing its reliability.

CONI-Net,^449^ or Component-Separable Neural Network, is another ML model aiming to parametrize an NCI force field. It is a set of four submodels, each designed to represent a specific additive component of the dimer NCI energy based on the SAPT partitioning. The network architecture is built on a Behler–Parrinello framework,^452,453^ which treats the interaction energy as a sum of two-atomic terms, which rely on FNNs. CONI-Net focuses exclusively on intermolecular interactions, while the intramolecular ones, which are beyond the scope of this work, are handled by the GROMOS force field.^454^ For features, CONI-Net uses intermolecular atomic pairs encoded as fingerprints. These do not contain explicit geometric information, but instead encode the local chemical environment of each atom of a pair as charges and bond orders within a respective monomer. Contributions of every fingerprint to the overall NCI energy are computed by four specialized subnets, each responsible for a specific component according to the SAPT scheme. Every subnet consists of 1) a fully connected neural network that interpolates between atomic pairs, and 2) a function layer containing multiple power-law terms to model the distance dependence. This design offers two key advantages. First, by treating the distance-dependence separately, the pair fingerprint only relies on the equilibrium monomer properties. Once computed, these can be reused throughout an MD simulation with no recalculation, thus reducing the amount of computational work. Second, the power-law terms enforce physically realistic mathematical forms for the distance dependencies. These terms ensure monotonic behavior, which prevents the model from learning unphysical oscillations and helps mitigate overfitting by constraining the model to follow expected physical trends (e.g., electrostatics decay as *r* ^–1^, dispersion as *r* ^–6^). Despite these constraints, using multiple power-law terms provides the model with sufficient flexibility to capture complex molecular interactions in a generalizable fashion.

The authors evaluate their model in two distinct applications. First, they use it in MD simulations by predicting thermodynamic properties, like mass density and enthalpy of vaporization, for organic solvents. Second, they test its transferability to larger hydrocarbons that are not included in the training set, demonstrating the ability of the model to generalize to unseen molecules. While the two models were trained on separate data sets, they utilize the same hyperparameters. For the first application, the model was trained on the CHNO data set of eight simple organic molecules, with 2000 homodimers per molecule, yielding 12,000 training and 4000 validation data points. The model predicted absolute dimer NCI energies with an MAE of 0.09 kcal mol^–1^, with the lowest errors in the induction and dispersion components (0.02 kcal mol^–1^), and higher errors for exchange and electrostatics (0.08 kcal mol^–1^ and 0.09 kcal mol^–1^, respectively). This variation is attributed to different interactions handling approximations differently, particularly due to challenges in modeling charge penetration and Pauli repulsion, which are angularly dependent and thus simplified in the isotropic interaction model. Bulk properties like enthalpy of vaporization and mass density were predicted with relative MAEs of 11.6% and 3.4%, respectively. For the second application, the model was trained on the CH data set (aliphatic and aromatic hydrocarbons) with 20,400 training and 6800 validation points, plus a test set of the same size. Unlike the CHNO-based MD simulations, the test set included molecules not present in the training and validation data sets. The total NCI energy was predicted with an MAE of 0.04 kcal mol^–1^, with MAEs of individual SAPT components ranging from 0.01–0.06 kcal mol^–1^. The model showed good generalization, predicting enthalpy of vaporization and mass density for unseen molecules with relative MAEs of 4.9% and 2.0%, respectively, which showcases good generalization power.

IPML^450^ is a physics-based intermolecular NCI potential, transferable across both small neutral organic molecules and larger biologically relevant systems. The model utilizes KRR and slightly deviates from the strict SAPT partitioning by considering five key interactions: electrostatics, charge penetration, repulsion, induction/polarization, and many-body dispersion. Overall, this leads to a total of 8 trainable parameters within IPML, which were derived by the ML models during training. The final parametrization of the NCI energies was performed using the S22×5 ^425^ small-molecule data set as a reference. Two models were trained based on the equilibrium distances from S22×5. The first model (labeled model 1 from now on) also incorporated off-equilibrium structures from the same data set (at 0.9 times the equilibrium separation), while the second model (model 2) was supplemented with host–guest complexes from the S12L database.^242^ Both models exhibited similar trained parameters. Interestingly, the prediction errors displayed a strong distance-dependence. More specifically, despite the overall MAE in model 1 of 0.7 kcal mol^–1^, the monomer separation-dependent MAE varied from 1.0 kcal mol^–1^ at 0.9 times the equilibrium separation to 0.2 kcal mol^–1^ at twice the equilibrium separation.

The authors benchmark IPML on a variety of data sets. The performance on the diverse S66×8 ^309^ NCI benchmark with CCSD­(T)/CBD-level energies was found to be excellent for most data points: MAEs of 0.4 kcal mol^–1^ for model 1 and 0.5 kcal mol^–1^ for model 2. Similarly, the SSI BFDb^306^ data set of side-chain interactions in proteins results in MAEs of 0.37 kcal mol^–1^ and 0.38 kcal mol^–1^, respectively. Testing on the JSCH-2005 data set, which includes representative DNA base and amino acid pairs,^311^ yielded significantly higher MAEs of 1.4 kcal mol^–1^ and 2.3 kcal mol^–1^, respectively. The authors attribute this to the presence of systems characterized by substantial π-stacking and hydrogen-bonding interactions, and to difficulties in predicting some quadrupole moments. In larger test systems, the errors also grow. For instance, IPML overstabilized the water clusters (consisting of 2–10 molecules)^455^ with an MAE of 8.1 kcal mol^–1^ for both models. However, the general trend is still captured correctly. Similar results were obtained for the S12L energies,^242^ where MAE reaches 9.7 kcal mol^–1^ for the first model. The authors do not provide an MAE for model 2 but it reproduces the energies significantly better, highlighting the importance of having a representative training set. Finally, IPML was tested on a benzene crystal. While it produced qualitatively correct results, both model 1 and model 2 overstabilized the crystal by 4.7 kcal mol^–1^ and 1.8 kcal mol^–1^, respectively. Notably, model 2 showed significantly less overstabilization, likely due to its differing training set, which included data from the S12L supramolecular benchmark. While model 1 performed worse on the benzene crystal, it provided better results for a benzene dimer. This suggests that incorporating supramolecular structures in the training set biases the model toward better predictions in larger systems, including the condensed phase.

CLIFF,^451^ or Component-based Machine-Learned Intermolecular Force Field, integrates accurate, physics-based equations for intermolecular interaction energies with ML. Similarly to IPML, the CLIFF model uses physics-derived functional forms for the electrostatic, exchange, induction/polarization, and London dispersion components, akin to the energy decomposition provided by SAPT. The authors note that while IPML includes terms that generally correspond to the SAPT components, its internal parameters are fit solely to total interaction energies, making the individual component energies inaccurate. IPML is thus unable to offer qualitative insights. As a result, the total interaction energies depend on a systematic cancellation of errors across different components, which cannot be guaranteed for a sufficiently diverse range of interaction types. This lack of accuracy in the individual components undermines the ability of the model to reliably predict interactions across distinct systems. As far as CLIFF is concerned, each of its component energies is fitted along with the total sum, making the model more general and robust.

CLIFF leverages aSLATM (atomic Spectrum of London and Axilrod–Teller-Muto potentials)^456,457^ to represent local atomic environments. These descriptors were used in separately trained KRR models for computing multipoles, atomic widths, and Hirshfeld ratios of all chemical elements used in the model. The calculated atomic parameters, along with additional global constants, were then applied in atom-pairwise interaction energy calculations for each SAPT-like component. Finally, a multitarget objective function was employed to minimize deviations in predicting both total and component NCI energies.

The training set for CLIFF consists of dimers from two collections, spanning 8 elements (C, H, N, O, S, F, Cl, and Br). The first collection includes 465 dimers generated by pairing 30 interaction sites of 23 monomers, with various intermolecular distances and orientations to capture short-range interactions, yielding 7000 configurations in total. The second collection includes 884 dimers at or near equilibrium, representing drug–protein interactions, spanning a range of energies from – 0.3 to – 27.5 kcal mol^–1^. The combined set contains 7884 dimers, ensuring that both short-range interactions and equilibrium geometries are well-represented for fitting the model.

The authors assess the performance and generalization of CLIFF on data sets like S66×8,^309^ SSI BFDb,^306^ X40×8,^320,458^ and NBC10,^459,460^ covering diverse interaction types, including both hydrogen and halogen bonding together with π-interactions. The predictions of the model, compared to the SAPT2+(3)­δMP2/aug-cc-pVTZ reference data, achieve MAEs of around 0.7 kcal mol^–1^ or better, with particularly low errors for the SSI and NBC10 benchmarks (0.28 kcal mol^–1^ and 0.49 kcal mol^–1^, respectively). However, errors increase for systems with repulsive interaction energies, especially in radial potential energy scans. In a subsequent study, the CLIFF methodology was also tested on two other small-molecule data sets with similar results.^461^

## 7 Designing Noncovalent Interactions

After having reviewed data sets, featurization strategies, and predictive ML models for NCIs, in this section we will explore the design of targets with optimized propensity for NCIs, guided by ML approaches. There are several approaches for doing so. Deep generative models, as unsupervised learning tools, can learn the statistical distribution of data sets and generate new data samples consistent with this distribution. Alternatively, data-driven optimization algorithms can enable target-directed molecular design, with reinforcement learning methods serving as a prominent example of this methodology. When combined with data characterizing NCIs, these approaches enable the deliberate design of NCIs within molecular systems. A notable example is the design of ligands that bind to specific proteins – a research area that has exploded in recent years due to its immediate relevance to drug discovery. It has been reviewed extensively by several authors.^462–464^ Rather than revisiting topics covered in other reviews, in this section, we want to highlight ML approaches applied beyond protein binding, focusing on the targeted design of NCIs in molecular systems. Despite there being only a handful of relevant papers, it is a rapidly emerging field with significant untapped potential, as NCIs represent a subtle balance of multiple weak forces, making their design a challenging and intricate task.

A notable example of molecular design aimed at optimizing NCIs was presented by Parrilla-Gutiérrez et al.,^465^ showcasing ML models trained on electron densities and molecular electrostatic potentials for the inverse design of guest molecules in host–guest complexes, with experimental validation of top candidates. This method was used to design binders for both cucurbit[6]­uril^466^ (CB[6]) and a Pd_2_L_4_ cage^467^ with 1,3-bis­(pyridin-3-ylethynyl)­benzene as ligand L. The authors implemented a two-stage workflow (Figure 19). In the first stage, an ML approach generated a virtual library of potential guests for a targeted host. In the second stage, human experts selected the most promising candidates for experimental testing via ^1^H NMR titration.^266^ The core component of the ML approach was a variational autoencoder (VAE) for the reconstruction of the 3D electron densities, trained on the QM9 data set, which consists of more than 130,000 molecules with up to 9 non-hydrogen atoms, specifically C, O, N, or F.^468,469^ This VAE compresses 3D electron densities, represented as a voxel grid similar to the commonly used CUBE format for storing electron densities, into a 1D latent space through its encoder. The decoder then uses this 1D latent space to regenerate the 3D electron densities. Additionally, the authors train a CNN to predict molecular electrostatic potentials on a 3D grid directly from 3D electron densities. The training data for the latter model were generated using the GFN2-xTB level of theory.^313^

**Figure 19:**
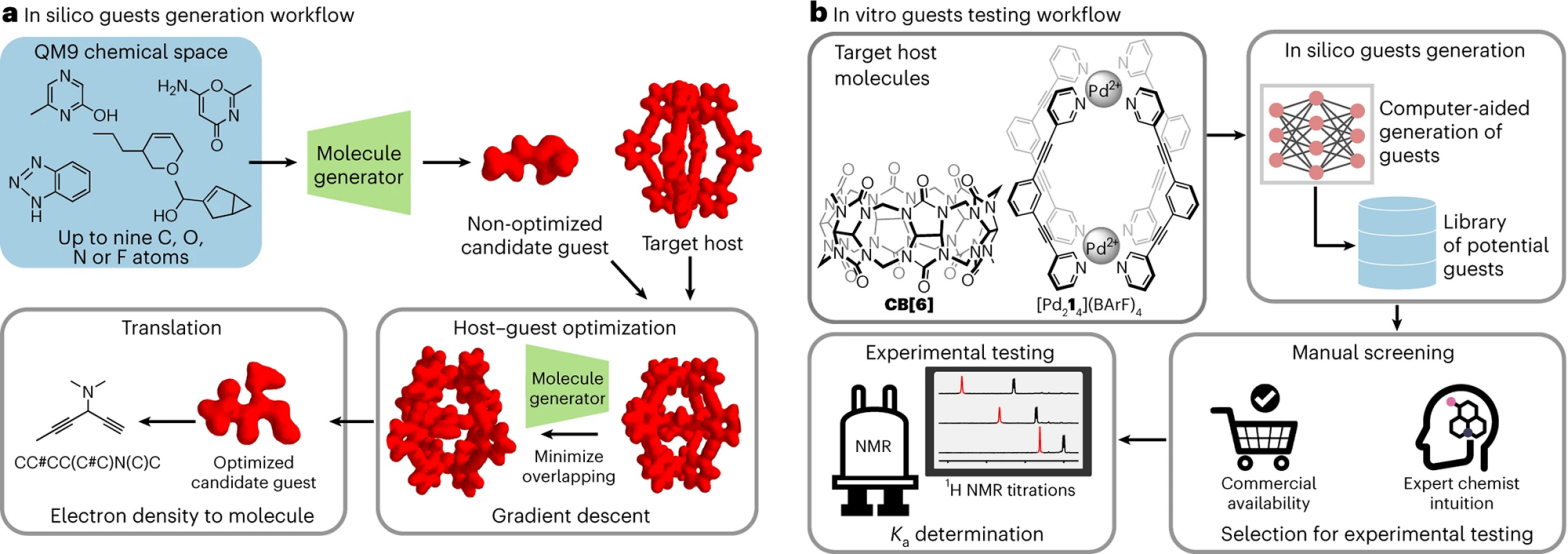
Two-stage workflow for the explicit design of small organic host molecules via a VAE for generating 3D electron densities.^465^

This VAE enabled the generation of molecules with better binding properties by navigating a 1D latent space, requiring only the 3D electron density of the target host. Molecules were optimized through gradient descent within the latent space to achieve three key objectives: 1) maximizing the size of the guest molecule, 2) minimizing exchange repulsion by reducing electron density overlap between the host and guest, and 3) maximizing electrostatic attraction by aligning their electrostatic potentials.

The process began with a random selection of molecules from the VAE’s latent space. These representations were decoded into 3D electron densities, which were then used to predict molecular electrostatic potentials through a trained CNN model. These properties determined the fitness of each molecule, guiding gradient updates in the latent space. By iteratively refining the molecules, the model produced new candidates with improved binding properties, effectively minimizing repulsion and maximizing attraction. In the end, the workflow yielded optimized 3D electron densities for better molecular design.

The final component of the ML approach was a Transformer model designed to translate generated 3D electron densities into SMILES representations,^470,471^ making it easier for human experts to interpret the proposed structures in the virtual library. This model followed a standard encoder-decoder architecture, where 3D electron densities were encoded into 1D latent sequences. The decoder processed tokenized SMILES sequences as both input and output, enabling the autoregressive generation of new molecules.

To bridge the gap between 3D electron densities and the Transformer’s 2D attention mechanism, the encoder’s embedding layer converted these densities into 2D attention matrices. This was done by first expanding the 3D electron densities into four dimensions, allowing the use of 3D convolutional layers in TensorFlow,^472^ and then compressing them back into two dimensions through convolutions. Instead of using raw 3D electron densities, the model took the pointwise product of electron densities and molecular electrostatic potentials as input, which improved translation accuracy from electron density to SMILES.

The virtual libraries obtained from this ML approach described above were inspected by human experts and the most promising structures, as judged based on their resemblance to known guests, their commercial availability, and the intuition of the experts, were tested experimentally via ^1^H NMR titration.^266^ The selected candidates consisted of both molecules that are very similar to known binders, following the intuition of the experts, and counterintuitive molecules with low resemblance of known binders. For CB[6], the algorithm rediscovered 9 known binders and uncovered 7 promising new binders, most of which had association constants larger than the ones of the 9 known binders. The strongest host discovered had an association constant more than 1 order of magnitude larger than the best association constant among the 9 known binders rediscovered. For the Pd_2_L_4_ cage, no previously known binders were rediscovered but 4 promising new binders were discovered. However, all of these new binders had relatively low binding affinities, which is largely because the corresponding molecules were quite small, exposing key limitations of the ML approach regarding host cavity size. The authors proposed that this limitation could be overcome by relying on data sets with larger molecules, such as included in the complete GDB-17 ^468^ data set.

## 8 Software Packages for Noncovalent Interactions

Before closing this review, we wish to inspect the available software packages that facilitate the study of NCIs. The development of reliable ML models requires reproducible workflows for data sets, featurization, training, and inference. For all these aspects, it is key that a code can be executed and utilized in a straightforward manner. User-friendly software interfaces, comprehensive documentation, permissible licenses for both data and code, and reliable metadata of software packages lead to higher adoption by peers, promoting follow-up research and advancing the field. Most of the studies discussed above come with dedicated code repositories that we will highlight in this section. In our experience, most research software of this kind is regrettably not maintained over the long-term, highlighting the importance of releasing code with comprehensive documentation.

Many of the featurization strategies accounting for NCIs have been implemented in dedicated code packages, which we will also discuss here. However, we will not cover programs that are primarily developed for molecular mechanics or electronic structure simulations. For an overview of the latter, we refer the reader to a recent special collection.^473^ A brief overview of the packages we discuss is provided in Table 5.

**Table 5:** Software Packages Facilitating the Development of ML Models for Studying NCIs

Software	Description	Documentation	Link
NCI Data Sets			
misato-data set^307^	Package for downloading the MISATO data set and for training and using ML models with it.	Yes	GitHub Repository
spice-data set^298^	Package with scripts and data files for the creation of the SPICE data set, facilitating its consistent extension.	No	GitHub Repository
NCI Featurization			
InteractionNet^355^	Package for setting up and using the InteractionNet model, including graph featurization.	No	GitHub Repository
ldp^406^	Package for computing the dispersion interaction potential *P*.	No	GitHub Repository
qml	Package for molecular representation learning with quantum chemistry-based featurization methods.	Yes	GitHub Repository
NCIPLOT^474^	Program for the identification of NCIs based on the reduced density gradient of the electron density.	Yes	Website
Multiwfn^475^	Program for electronic wave function analysis enabling quantum chemistry-based featurization.	Yes	Website
py2sambvca^476,477^	Package for computing buried volumes of chemical structures.	Yes	GitHub Repository
DBSTEP	Package for computing Sterimol parameters and buried volumes for chemical structures.	Yes	GitHub Repository
morfeus	Package for computing the dispersion interaction potential, buried volume, and Sterimol parameters.	Yes	GitHub Repository
wSterimol^378^	Package for computing Boltzmann-weighted Sterimol parameters for chemical structures.	Yes	GitHub Repository
NCI Prediction			
delfta^314^	Package for training DelFTa models, result analysis, and predicting formation and orbital energies, dipoles, Mulliken partial charges, and Wiberg bond orders.	Yes	GitHub Repository
cliff^451^	Package for training CLIFF models, analyzing the results, and predicting interaction energies decomposed into electrostatics, exchange, induction, and dispersion.	Yes	GitHub Repository
XB-ML^423^	Package for training and using models to characterize and predict halogen bonding.	No	GitHub Repository
TFRegNCI^439^	Package for training TFRegNCI models and analyzing the results for NCI-corrections to electronic energies.	No	GitHub Repository
AP-Net^445^	Package for training AP-Net models, analyzing the results, and predicting interaction energies decomposed into electrostatics, exchange, induction, and dispersion.	No	GitHub Repository
ipml^445^	Package for featurization, training IPML models, analysis, and predicting interaction energies decomposed into electrostatics, charge penetration, repulsion, induction, and many-body dispersion.	Yes	GitHub Repository
NCI Design			
electrondensity2 ^465^	Package for generating training data, training models, and use them to design guest molecules based on host electron densities.	No	GitHub Repository

### 8.1 Packages for Data Sets

Data sets themselves are usually shared on dedicated data repositories rather than on code repositories. Additionally, the platforms used for publishing code are not suitable for hosting large data sets because they are optimized for handling many small files rather than a few large ones. Furthermore, simply publishing a data set in a dedicated repository does not equate to creating a software package. Therefore, in this section, we will focus on packages that are not just data repositories but also provide a code that facilitates the utilization of the corresponding data, or even go beyond that.

The **misato-data set** Python package^307^ not only provides detailed instructions for how to download the full data set from Zenodo, but also comes with a small subset of the data for demonstration and testing purposes. Additionally, it provides examples illustrating the structure of the data and a custom code for loading and using the data with PyTorch.^478^ We believe that the provided code, along with the accompanying tutorials, enhances the usability of the data for various applications and thus stimulates further research.

The **spice-data set** Python package^298^ is a rare example of a data set repository that provides scripts and data files used for the creation of the SPICE data set. This allows not only assessing the reliability of the provided data but also enables augmenting it with new data points in a systematic manner. Accordingly, this facilitates developing ML models with improved performance compared to the reference implementation described in the associated publication. We believe that this approach should be embraced by other data sets, as it not only simplifies data verification but also enables the community to seamlessly extend systematic data sets.

### 8.2 Packages for Featurization

Molecular featurization for the purpose of developing ML models has received great attention in recent years.^479^ It is the backbone of any ML model and often determines what type of ML architecture is best to be used. Accordingly, it is crucial that the corresponding tools for featurization are user-friendly and well-documented in order to facilitate their adoption. Whereas a large number of featurization approaches are implemented in the standard cheminformatics and molecular ML packages such as rdkit^480^ and deepchem,^481^ they often lack informative representations tailored to modeling NCIs. This underscores the need for further developments in this research area. In this section, we highlight software packages that allow for featurizing NCIs.

Among the featurization approaches discussed in previous sections, the one employed by **InteractionNet** ^355^ stands out, as it uses graph representation learning to describe both covalent bonds and NCIs equally, through dedicated edges. Therefore, pretrained models can readily be used as learned representation for downstream prediction tasks, making this approach highly attractive. Unfortunately, the code repository only provides the Python code as is with no usage instructions.

The **ldp** ^406^ Python package allows computing London dispersion potential maps and average London dispersion potentials of molecules or functional groups. It only provides minimal installation and usage instructions with one example for each of the two main functions. Moreover, it relies on external programs to compute dispersion coefficients and generate molecular surfaces, both of which require manual installation, making the process cumbersome.

The **qml** ^482^ Python tooklit for quantum ML comes with extensive documentation and usage instructions, and facilitates the entire ML model development workflow from featurization to training different models and making predictions. The implemented featurization strategies include several representations based on the Coulomb matrix and the SLATM of a molecule, both potentially useful for modeling NCIs. The latter representation has been used in the CLIFF^451^ model discussed above.

**NCIPLOT** ^474^ is a standalone program that was originally developed for visualizing NCIs^483^ based on electron density-based analysis and has reached wide adoption by the research community. It relies on the so-called reduced density gradient that is derived from the electron density.^474^ Apart from its power as a visualization tool, NCIPLOT reveals points in space associated with NCIs, which can be used directly as an explicit representation of NCIs for ML, as demonstrated in recent proof-of-concept demonstrations for correcting predicted electronic energies from DFT (*vide supra*).^427,430,431^

Similarly, the standalone **Multiwfn** program^475^ has been developed to conduct comprehensive wave function- and electron density-based analysis. It implements a very large number of different approaches, which are documented in a detailed manual provided by the developer. It implements the van der Waals potential and it also provides an alternative implementation of the NCIPLOT methodology based on the reduced density gradient and can therefore also be used for direct featurization of NCIs.

The **py2sambvca** ^476,477^ is a Python package that serves as a thin client to interface Python scripts with the **SambVca** Fortran program,^484^ which calculates the buried volume and topographic steric maps of ligands. The package comes with installation instructions and usage information about the functions it provides. **SambVca** needs to be compiled and installed separately. This representation has been extensively used for the study of transition metal catalysts.^485^

The **DBSTEP** ^486^ is a Python package with a Fortran backend that computes the Sterimol parameters and the buried volume based on molecular structure. It is extensively documented and also provides a detailed installation guide and usage instructions. The steric parameters that **DBSTEP** computes have been widely used for ML models predicting reaction or catalyst performance, and are widely used as general design handles for activity and selectivity (*vide supra*).

The **morfeus** ^487^ Python package not only provides an alternative implementation of the Sterimol parameters and the buried volume based on molecular structures, but also implements the Tolman cone angle. Additionally, it comes with a standalone implementation of the average London dispersion potentials of molecules and functional groups, without requiring the installation of external programs. Finally, it provides an interface to the **xtb** ^488^ program, allowing simple access to additional molecular properties that are relevant to studying NCIs, such as the dipole moment. It also comes with a comprehensive documentation and many usage examples.

The **wSterimol** Python package^378^ implements a recently proposed extension of the classical rigid Sterimol parameters to improve the featurization of steric effects in flexible molecules. It implements computation of the Boltzmann-weighted Sterimol parameters which account for conformational flexibility. This package is well-documented and provides detailed instructions for usage and instructive tutorials.

### 8.3 Packages for Prediction

ML packages for predicting NCIs should allow others to test models on new data and use them in applications. To be useful, these packages must include either pretrained models or training code along with the necessary data sets.

From the NCI-predicting ML models that were discussed earlier, **delfta**,^314^ **cliff**,^451^ and **ipml** ^445^ packages offer clear installation and usage guides, making them accessible to the community. While ipml focuses on featurization and training new models, delfta and cliff provide tutorials for using pretrained models to make predictions. Additionally,ipml includes the full data set from its corresponding publication as a part of the repository. All three packages offer pretrained model parameters, enabling immediate application to molecular systems.

In contrast, **XB-ML**,^423^ **TFRegNCI**,^439^ and **AP-Net** ^445^ only provide minimal information on how to install the packages or use the provided code. Nevertheless, both the TFRegNCI and AP-Net come with pretrained models. The XB-ML package includes the full data sets used for training, which is also the case for AP-Net. All these three packages come with the code for performing the required featurization and training the corresponding models based on correctly formatted data. Therefore, they can all be applied to new problems and, thus, are a strong foundation for further research.

### 8.4 Packages for Design

Packages enabling the design of NCIs can be used to generate molecules with desired properties, such as favorable binding affinity to a well-defined target. Ideally, such packages should also provide either pretrained models or a code for training, together with the training data.

The **electrondensity2** ^465^ Python package, used by Parrilla-Gutiérrez et al. for the design of optimized guests in host–guest complexes (*vide supra*), comes with installation instructions and a description of how the original training data set can be regenerated. The bulk of the installation can be carried out with package management software like conda. However, the orbkit Python package,^489^ on which **electrondensity2** relies, requires manual installation. The full training data set is not provided as it is prohibitively large (indicated as almost 300 GB in total). In addition, the package provides means to train all models used in the corresponding study (*vide supra*) and use them for the design of new small organic molecule guests. Trained models are shared by the authors via a dedicated data repository.^490^

## 9 Challenges and Outlook

Based on the previous sections, it is clear that the study of NCIs with ML is still in its early days. Whereas ML has already made a significant impact on molecular property prediction^491^ and inverse molecular design,^492^ it has yet to reach maturity and unlock its full potential for studying NCIs in molecular systems. We believe that this is in part due to the inherently challenging nature of studying NCIs, be it experimentally, computationally, or both.^493^ NCIs are intrinsically weak in terms of strength, and, in a particular molecular system, they rarely occur in isolation. Instead, they often involve a complex interplay of multiple, sometimes counteracting, interactions. Nevertheless, several studies we highlighted above already provide a glimpse of the unique potential ML can offer to advance research on NCIs. In this section, we outline the current challenges within this emerging field, which may serve as a guide for future research efforts.

The first challenge concerns the available data sets that characterize NCIs, in particular the aspects of quality, diversity, and size. In terms of data set quality, the field would strongly benefit from systematic high-quality data sets determined by experiment. While a significant amount of experimental data has been published in recent decades, they are extremely scattered, often with only up to a handful of individual data points per study. Additionally, the majority of such studies only provides experimentally determined association free energies without any detailed structural information. While, in principle, this does not prevent the corresponding data from being used for downstream ML applications, the absence of simple molecular graph NCI representations and the fact that 3-dimensional structures of molecular systems with NCIs are generally not straightforward to generate, make this data hard to utilize. Generating 3-dimensional structures for such systems, at the very least, requires comprehensive exploration of the corresponding conformer space, which is computationally expensive. One way forward is to apply data mining on a large scale to uncover relevant data from external sources, which can then be integrated with the available data using high-throughput virtual screening to enhance it with structural information. This could give rise to comparably large composite data sets with an estimated number of data points on the order of 10^3^, which combine both experimental and computational information, similar to how computational composite data sets like GMTKN55 ^212^ or the Atlas family of data sets^290^ have been created. We envision that this would blur the boundaries between computational and experimental data sets and, ultimately, lead to ML models with higher accuracy for predicting NCIs. In terms of data set diversity, the molecular space covered by current data sets is largely limited to small- and medium-sized molecular systems consisting of main group elements in the ground state. Hence, systematic computational data sets characterizing NCIs in transition metal compounds and excited states are needed. In terms of size, while recent years have brought forth a significant growth in the number of data points per data set concerning NCIs, we believe that, in the coming years, we will undoubtedly witness further growth by several orders of magnitude toward billions.

The second challenge concerns featurization strategies for NCIs. As can be deduced from the highlighted studies in this review, molecular featurization approaches accounting for NCIs are still not routine as they are not implemented in standard cheminformatics or molecular ML packages. In part, this is because the development of effective molecular features for NCIs is still very much ongoing research that has yet to uncover both simple and expressive molecular representations. For instance, we believe that there is significant promise in the recently developed approaches relying on promolecular electron densities for leading to considerable advances in featurization.^427,430,431^ Another avenue could be the application of highly efficient and scalable semiempirical quantum chemistry methods as backbone for new molecular representation. Nevertheless, apart from the need for such novel molecular featurization strategies for NCIs, many of the existing approaches discussed in this review have reached sufficient maturity for their inclusion in mainstream cheminformactics packages. We believe that this will not only stimulate the adoption of ML approaches in the study of NCI but also improve the quality of ML models, even when NCIs are not an explicit subject of research.

The third challenge concerns the use of effective training strategies for enabling self-supervised or unsupervised learning at scale for NCIs. Essentially all the ML models discussed in this review rely solely on supervised learning, which requires labeled data of high quality in sufficient amounts. However, recent advancements in deep learning show that the amount of labeled NCI data currently available, or likely to become available in the near future, is insufficient to achieve the desired model performance. As a result, alternative strategies, particularly self-supervised or unsupervised learning, need to be explored.^494^ In the context of NCIs, self-supervised or unsupervised learning at scale could give rise to so-called foundation models^495^ that would allow for effective fine-tuning on relatively small specialized data sets concerning a specific family of NCIs to provide accurate predictions. In particular, we envision that training such a foundation model on simulated data at scale, followed by fine-tuning on the comparably small experimental data sets, could unlock unprecedented prediction accuracies. This also has the potential to uncover new families of NCIs that have been overlooked so far.

The fourth and final challenge we will discuss is the development of deep generative models for NCIs. Whereas molecular deep generative models are a very popular research subject in ML for chemistry,^496^ their use for the explicit design of NCIs has yet to make significant advances. Nevertheless, we believe that designing NCIs is an area primed to be tackled with ML as it necessitates accounting for complex patterns of nuanced interactions and immense design spaces, making it extremely challenging for classical expert-led design. Current molecular deep generative models, in principle, are already powerful enough to address this challenging task, as long as they are incorporated in iterative computational design-make-test-analyze cycles,^492^ allowing for continuous model refinement as molecular designs improve at every iteration. This has the potential to establish new structural motifs giving rise to uniquely strong NCIs which have been overlooked until now. To drive progress in this area, we propose the development of a dedicated computational benchmark for the inverse design of molecular systems relying solely on NCIs with strong interaction energies. Such a benchmark, inspired by the recent systematic suite TARTARUS,^35^ could encourage broader adoption among both chemists and ML experts. We are looking forward to the significant advancements in molecular deep generative models for NCIs that are sure to emerge in the coming years.

## 10 Conclusion

Overall, it is clear that the study of NCIs with ML is an emerging field that is beginning to show its potential. In this review, we have aimed to provide a comprehensive overview of current developments in this area. We believe that NCIs and ML are two areas that are bound to come together as the inherent complexity and subtlety of NCIs lends itself perfectly to ML approaches. In recent years, promising new directions emerged, in particular the use of quantum chemical featurization strategies for NCIs and the analysis of feature importance in ML models to locate significant NCIs. Nevertheless, it is clear that NCIs are still a niche area in the larger ML for chemistry research domain, and we feel that it is particularly overlooked by researchers without a chemistry background. To counteract this bias, we not only highlighted recent research in this area but also explicitly discussed current challenges to be tackled in the near future. One promising avenue for raising awareness is the development of a dedicated ML competition for predicting and designing NCIs, akin to how the Critical Assessment of Structure Prediction (CASP) challenge has shaped the landscape of ML models for protein structure prediction.^497^ We believe that this will both increase the visibility of this topic and, ultimately, enable the wider research community to tackle longstanding challenges, such as the design of arbitrarily large molecular systems with bespoke NCIs at will.

## References

[1] Müller-Dethlefs K., Hobza P. (2000). Noncovalent Interactions: A Challenge for Experiment and Theory. Chem. Rev..

[2] Casitas A., Rees J. A., Goddard R., Bill E., DeBeer S., Fürstner A. (2017). Two Exceptional Homoleptic Iron­(IV) Tetraalkyl Complexes. Angew. Chem., Int. Ed..

[3] Rösel S., Quanz H., Logemann C., Becker J., Mossou E., Cañadillas-Delgado L., Caldeweyher E., Grimme S., Schreiner P. R. (2017). London dispersion enables the shortest
intermolecular hydrocarbon H··· H contact. J. Am. Chem. Soc..

[4] Rösel S., Becker J., Allen W. D., Schreiner P. R. (2018). Probing
the delicate balance between pauli repulsion and London dispersion
with triphenylmethyl derivatives. J. Am. Chem.
Soc..

[5] Askeland, D. ; Wright, W. The Science and Engineering of Materials; Cengage Learning: Boston, MA, 2015; p 38.

[6] Lu M., Rao S., Yue H., Han J., Wang J. (2024). Recent Advances in
the Application of Machine Learning to Crystal Behavior and Crystallization
Process Control. Cryst. Growth Des..

[7] MacDougall F. H. (1916). The Equation
of State for Gases and Liquids. J. Am. Chem.
Soc..

[8] Elliott, J. R. ; Diky, V. ; Knotts, T. A. ; Wilding, W. V. The Properties of Gases and Liquids, sixth ed.; McGraw-Hill’s AccessEngineeringLibrary; McGraw Hill LLC: New York, NY, 2023.

[9] Rothe E. W., Bernstein R. B. (1959). Total Collision Cross Sections for
the Interaction
of Atomic Beams of Alkali Metals with Gases. J. Chem. Phys..

[10] Jankowski P., McKellar A. R. W., Szalewicz K. (2012). Theory Untangles the High-Resolution
Infrared Spectrum of the *Ortho*-H_2_-CO van
Der Waals Complex. Science.

[11] Yang B., Zhang P., Wang X., Stancil P., Bowman J., Balakrishnan N., Forrey R. (2015). Quantum Dynamics of CO–H_2_ in Full
Dimensionality. Nat. Commun..

[12] Novoa, J. J. Chapter 2: Using Computational Quantum Chemistry as a Tool to Understand the Structure of Molecular Crystals and the Nature of Their Intermolecular Interactions. In Intermolecular Interactions in Crystals: Fundamentals of Crystal Engineering; Novoa, J. J. , Ed.; The Royal Society of Chemistry, 2017; pp 69–114.

[13] Politzer, P. ; Murray, J. S. ; Clark, T. Chapter 10: Intermolecular Interactions in Crystals. In Intermolecular Interactions in Crystals: Fundamentals of Crystal Engineering; Novoa, J. J. , Ed.; The Royal Society of Chemistry, 2017; pp 375–409.

[14] Gavezzotti, A. Chapter 3: Bonding in Organic Molecules and Condensed Phases. The Role of Repulsions. In Intermolecular Interactions in Crystals: Fundamentals of Crystal Engineering; Novoa, J. J. , Ed.; The Royal Society of Chemistry, 2017; pp 115–146.

[15] Edwards A. J., Mackenzie C. F., Spackman P. R., Jayatilaka D., Spackman M. A. (2017). Intermolecular Interactions in Molecular Crystals:
What’s in a Name?. Faraday Discuss..

[16] Intermolecular Interactions in Crystals: Fundamentals of Crystal Engineering; Novoa, J. J. , Ed.; Royal Society of Chemistry: London, 2018.

[17] Brandenburg J. G., Maas T., Grimme S. (2015). Benchmarking DFT and
Semiempirical
Methods on Structures and Lattice Energies for Ten Ice Polymorphs. J. Chem. Phys..

[18] Brandenburg J. G., Grimme S. (2016). Organic Crystal
Polymorphism: A Benchmark for Dispersion-Corrected
Mean-Field Electronic Structure Methods. Acta
Crystallogr., Sect. B: Struct. Sci., Cryst. Eng. Mater..

[19] Odoh S. O., Cramer C. J., Truhlar D. G., Gagliardi L. (2015). Quantum-Chemical
Characterization of the Properties and Reactivities of Metal–Organic
Frameworks. Chem. Rev..

[20] Reichardt, C. ; Welton, T. Solvents and Solvent Effects in Organic Chemistry, 4th ed.; Wiley-VCH: Weinheim, Germany, 2011.

[21] Knowles R.
R., Jacobsen E. N. (2010). Attractive
Noncovalent Interactions in Asymmetric Catalysis:
Links between Enzymes and Small Molecule Catalysts. Proc. Natl. Acad. Sci. U.S.A..

[22] Jena S., Dutta J., Tulsiyan K. D., Sahu A. K., Choudhury S. S., Biswal H. S. (2022). Noncovalent Interactions
in Proteins and Nucleic Acids:
Beyond Hydrogen Bonding and *π*-Stacking. Chem. Soc. Rev..

[23] Pravda L., Berka K., Svobodová Vařeková R., Sehnal D., Banáš P., Laskowski R. A., Koča J., Otyepka M. (2014). Anatomy of Enzyme Channels. BMC Bioinf..

[24] Fersht, A. Enzyme Structure and Mechanism, 2nd ed.; W. H. Freeman: New York, 1985.

[25] Chiti F., Dobson C. M. (2006). Protein Misfolding,
Functional Amyloid, and Human Disease. Annu.
Rev. Biochem..

[26] Dobson C. M. (2003). Protein
Folding and Misfolding. Nature.

[27] All Nobel Prizes 2024. https://www.nobelprize.org/all-nobel-prizes-2024 (accessed February 23, 2025).

[28] Wu Z., Ramsundar B., Feinberg E. N., Gomes J., Geniesse C., Pappu A. S., Leswing K., Pande V. (2018). MoleculeNet: A Benchmark
for Molecular Machine Learning. Chem. Sci..

[29] Haghighatlari M., Vishwakarma G., Altarawy D., Subramanian R., Kota B. U., Sonpal A., Setlur S., Hachmann J. (2020). ChemML: A
Machine Learning and Informatics Program Package for the Analysis,
Mining, and Modeling of Chemical and Materials Data. Wiley Interdiscip. Rev. Comput. Mol. Sci..

[30] Nigam A., Pollice R., Hurley M. F., Hickman R. J., Aldeghi M., Yoshikawa N., Chithrananda S., Voelz V. A., Aspuru-Guzik A. (2021). Assigning
Confidence to Molecular Property Prediction. Expert Opin. Drug Discovery.

[31] Heid E., Greenman K. P., Chung Y., Li S.-C., Graff D. E., Vermeire F. H., Wu H., Green W. H., McGill C. J. (2024). Chemprop:
A Machine Learning Package for Chemical Property Prediction. J. Chem. Inf. Model..

[32] Gómez-Bombarelli R., Wei J. N., Duvenaud D., Hernández-Lobato J. M., Sánchez-Lengeling B., Sheberla D., Aguilera-Iparraguirre J., Hirzel T. D., Adams R. P., Aspuru-Guzik A. (2018). Automatic
Chemical Design Using a Data-Driven Continuous Representation of Molecules. ACS Cent. Sci..

[33] García-Ortegón M., Simm G. N., Tripp A. J., Hernández-Lobato J. M., Bender A., Bacallado S. (2022). DOCKSTRING:
Easy Molecular Docking
Yields Better Benchmarks for Ligand Design. J. Chem. Inf. Model..

[34] Seumer J., Kirschner Solberg Hansen J., Bro̷ndsted
Nielsen M., Jensen J. H. (2023). Computational Evolution of New Catalysts
for the Morita–Baylis–Hillman
Reaction. Angew. Chem., Int. Ed..

[35] Nigam A., Pollice R., Tom G., Jorner K., Willes J., Thiede L., Kundaje A., Aspuru-Guzik A. (2023). Tartarus:
A Benchmarking Platform for Realistic and Practical Inverse Molecular
Design. Adv. Neural Inf. Process. Syst..

[36] Loeffler H. H., He J., Tibo A., Janet J. P., Voronov A., Mervin L. H., Engkvist O. (2024). Reinvent 4:
Modern AI–Driven Generative Molecule
Design. J. Cheminf..

[37] Häse F., Roch L. M., Kreisbeck C., Aspuru-Guzik A. (2018). Phoenics:
A Bayesian Optimizer for Chemistry. ACS Cent.
Sci..

[38] Shields B. J., Stevens J., Li J., Parasram M., Damani F., Alvarado J. I. M., Janey J. M., Adams R. P., Doyle A. G. (2021). Bayesian
Reaction Optimization as a Tool for Chemical Synthesis. Nature.

[39] Slattery A., Wen Z., Tenblad P., Sanjosé-Orduna J., Pintossi D., den Hartog T., Noël T. (2024). Automated
Self-Optimization, Intensification,
and Scale-up of Photocatalysis in Flow. Science.

[40] Lopez S. A., Sanchez-Lengeling B., de Goes Soares J., Aspuru-Guzik A. (2017). Design Principles
and Top Non-Fullerene Acceptor Candidates for Organic Photovoltaics. Joule.

[41] Schwaller P., Probst D., Vaucher A. C., Nair V. H., Kreutter D., Laino T., Reymond J.-L. (2021). Mapping the Space of Chemical Reactions
Using Attention-Based Neural Networks. Nat.
Mach. Intell..

[42] Wellawatte G. P., Seshadri A., White A. D. (2022). Model Agnostic Generation of Counterfactual
Explanations for Molecules. Chem. Sci..

[43] Boiko D. A., MacKnight R., Kline B., Gomes G. (2023). Autonomous Chemical
Research with Large Language Models. Nature.

[44] Bran A. M., Cox S., Schilter O., Baldassari C., White A. D., Schwaller P. (2024). Augmenting
Large Language Models with Chemistry Tools. Nat. Mach. Intell..

[45] Huang B., von Lilienfeld O. A. (2021). Ab Initio Machine Learning in Chemical
Compound Space. Chem. Rev..

[46] Keith J. A., Vassilev-Galindo V., Cheng B., Chmiela S., Gastegger M., Müller K.-R., Tkatchenko A. (2021). Combining Machine Learning and Computational
Chemistry for Predictive Insights into Chemical Systems. Chem. Rev..

[47] Westermayr J., Marquetand P. (2021). Machine Learning for Electronically
Excited States
of Molecules. Chem. Rev..

[48] Noé F., Tkatchenko A., Müller K.-R., Clementi C. (2020). Machine Learning for
Molecular Simulation. Annu. Rev. Phys. Chem..

[49] Unke O. T., Chmiela S., Sauceda H. E., Gastegger M., Poltavsky I., Schütt K. T., Tkatchenko A., Müller K.-R. (2021). Machine Learning Force Fields. Chem. Rev..

[50] Szalewicz K. (2012). Symmetry-Adapted
Perturbation Theory of Intermolecular Forces. Wiley Interdiscip. Rev. Comput. Mol. Sci..

[51] Zhao L., von Hopffgarten M., Andrada D. M., Frenking G. (2018). Energy Decomposition
Analysis. Wiley Interdiscip. Rev. Comput. Mol.
Sci..

[52] Mao Y., Loipersberger M., Horn P. R., Das A., Demerdash O., Levine D. S., Prasad Veccham S., Head-Gordon T., Head-Gordon M. (2021). From Intermolecular Interaction Energies and Observable
Shifts to Component Contributions and Back Again: A Tale of Variational
Energy Decomposition Analysis. Annu. Rev. Phys.
Chem..

[53] Bistoni G., Altun A., Wang Z., Neese F. (2024). Local Energy Decomposition
Analysis of London Dispersion Effects: From Simple Model Dimers to
Complex Biomolecular Assemblies. Acc. Chem.
Res..

[54] London F. (1930). Zur Theorie
Und Systematik Der Molekularkräfte. Z.
Phys..

[55] Heisenberg W. (1926). Mehrkörperproblem
Und Resonanz in Der Quantenmechanik. Z. Phys..

[56] Dirac P. A. M. (1926). On the
Theory of Quantum Mechanics. Proc. R. Soc. London,
Ser. A.

[57] Bent H. A. (1968). Structural
Chemistry of Donor-Acceptor Interactions. Chem.
Rev..

[58] Wu P., Chaudret R., Hu X., Yang W. (2013). Noncovalent Interaction
Analysis in Fluctuating Environments. J. Chem.
Theory Comput..

[59] Kollman P.
A., Allen L. C. (1972). Theory
of the Hydrogen Bond. Chem. Rev..

[60] Cavallo G., Metrangolo P., Milani R., Pilati T., Priimagi A., Resnati G., Terraneo G. (2016). The Halogen Bond. Chem. Rev..

[61] Riley K. E., Hobza P. (2013). On the Importance and
Origin of Aromatic Interactions in Chemistry
and Biodisciplines. Acc. Chem. Res..

[62] Truhlar D. G. (2019). Dispersion
Forces: Neither Fluctuating nor Dispersing. J. Chem. Educ..

[63] Atkins, P. W. ; de Paula, J. ; Keeler, J. J. Atkins’ Physical Chemistry, eleventh ed.; Oxford University Press: Oxford, New York, 2018.

[64] IUPAC Gold Book – Van Der Waals Forces. 2019; https://goldbook.iupac.org/terms/view/V06597 (accessed February 23, 2025).

[65] Pauli W. (1925). Über
Den Zusammenhang Des Abschlusses Der Elektronengruppen Im Atom Mit
Der Komplexstruktur Der Spektren. Z. Phys..

[66] Lennard-Jones J. E. (1931). Cohesion. Proc.
Phys. Soc..

[67] Buckingham R. A. (1938). The Classical
Equation of State of Gaseous Helium, Neon and Argon. Proc. R. Soc. London, Ser. A.

[68] Rackers, J. A. ; Ponder, J. W. Classical Pauli Repulsion: An Anisotropic, Atomic Multipole Model. J. Chem. Phys. 2019, 150. 10.1063/1.5081060. PMC6386640 30823770

[69] Azar R. J., Head-Gordon M. (2012). An Energy
Decomposition Analysis for Intermolecular
Interactions from an Absolutely Localized Molecular Orbital Reference
at the Coupled-Cluster Singles and Doubles Level. J. Chem. Phys..

[70] Wagner J. P., Schreiner P. R. (2015). London Dispersion in Molecular Chemistry—Reconsidering
Steric Effects. Angew. Chem., Int. Ed..

[71] Wang S., Hou K., Heinz H. (2021). Accurate and
Compatible Force Fields for Molecular
Oxygen, Nitrogen, and Hydrogen to Simulate Gases, Electrolytes, and
Heterogeneous Interfaces. J. Chem. Theory Comput..

[72] Eisenschitz R., London F. (1930). Über Das Verhältnis
Der van Der Waalsschen
Kräfte Zu Den Homöopolaren Bindungskräften. Z. Phys..

[73] Koide A. (1976). A New Expansion
for Dispersion Forces and Its Application. Proc.
Phys. Soc., London.

[74] van der Waals, J. D. The Equation of State for Gases and Liquids. In Nobel Lectures, Physics 1901–1921; Elsevier Publishing Company: Amsterdam, 1967; pp 254–265.

[75] Barker J. A. (1982). Van Der
Waals Molecules and Condensed Phases. Faraday
Discuss. Chem. Soc..

[76] Pollice R., Fleckenstein F., Shenderovich I., Chen P. (2019). Compensation of London
Dispersion in the Gas Phase and in Aprotic Solvents. Angew. Chem., Int. Ed..

[77] Gravillier L.-A., Cockroft S. L. (2023). Context-Dependent Significance of London Dispersion. Acc. Chem. Res..

[78] Rummel L., Schreiner P. R. (2024). Advances and Prospects in Understanding
London Dispersion
Interactions in Molecular Chemistry. Angew.
Chem., Int. Ed..

[79] Salem L. (1961). The Forces
between Polyatomic Molecules. II. Short-range Repulsive Forces. Proc. R. Soc. London, Ser. A.

[80] Bader R. F. (2006). Pauli Repulsions
Exist Only in the Eye of the Beholder. Chem.
Eur. J..

[81] Slater J. C. (1928). The Normal
State of Helium. Phys. Rev..

[82] Hamlin T. A., Bickelhaupt F. M., Fernández I. (2021). The Pauli Repulsion-Lowering Concept
in Catalysis. Acc. Chem. Res..

[83] Bauzá A., Frontera A. (2015). Aerogen Bonding Interaction:
A New Supramolecular Force?. Angew. Chem., Int.
Ed..

[84] Scheiner S. (2021). Dissection
of the Origin of *π*-Holes and the Noncovalent
Bonds in Which They Engage. J. Phys. Chem. A.

[85] Mallada B., Ondráček M., Lamanec M., Gallardo A., Jiménez-Martín A., De La Torre B., Hobza P., Jelínek P. (2023). Visualization
of *π*-Hole in Molecules by Means of Kelvin Probe
Force Microscopy. Nat. Commun..

[86] Arunan E., Desiraju G. R., Klein R. A., Sadlej J., Scheiner S., Alkorta I., Clary D. C., Crabtree R. H., Dannenberg J. J., Hobza P. (2011). Definition
of the Hydrogen Bond (IUPAC Recommendations
2011). Pure Appl. Chem..

[87] Steiner T. (2002). The Hydrogen
Bond in the Solid State. Angew. Chem., Int.
Ed..

[88] Meot-Ner M. (2005). The Ionic
Hydrogen Bond. Chem. Rev..

[89] Kollman P. A. (1972). Theory
of Hydrogen Bond Directionality. J. Am. Chem.
Soc..

[90] Shahi A., Arunan E. (2016). Why Are Hydrogen Bonds Directional?. Chem. Sci. J..

[91] Umeyama H., Morokuma K. (1977). The Origin of Hydrogen Bonding. An
Energy Decomposition
Study. J. Am. Chem. Soc..

[92] Sokalski W. A., Hariharan P. C., Kaufman J. J. (1983). A Self-Consistent Field Interaction
Energy Decomposition Study of 12 Hydrogen-Bonded Dimers. J. Phys. Chem..

[93] Fersht, A. Structure and Mechanism in Protein Science: A Guide to Enzyme Catalysis and Protein Folding; Macmillan, 1999.

[94] Li X.-Z., Walker B., Michaelides A. (2011). Quantum Nature
of the Hydrogen Bond. Proc. Natl. Acad. Sci.
U.S.A..

[95] Shaik, S. Chapter 1: Bonds and Intermolecular Interactions – The Return of Cohesion to Chemistry. In Intermolecular Interactions in Crystals: Fundamentals of Crystal Engineering; Novoa, J. J. , Ed.; The Royal Society of Chemistry, 2017; pp 1–68.

[96] Van
der Lubbe S. C., Fonseca Guerra C. (2019). The Nature of Hydrogen Bonds: A Delineation
of the Role of Different Energy Components on Hydrogen Bond Strengths
and Lengths. Chem. Asian J..

[97] Moore T. S., Winmill T. F. (1912). CLXXVII.—The
State of Amines in Aqueous Solution. J. Chem.
Soc., Trans..

[98] Latimer W. M., Rodebush W. H. (1920). Polarity and Ionization from the Standpoint of the
Lewis Theory of Valence. J. Am. Chem. Soc..

[99] Bolen D. W., Rose G. D. (2008). Structure and Energetics
of the Hydrogen-Bonded Backbone
in Protein Folding. Annu. Rev. Biochem..

[100] Liu Y., Wang L., Zhao L., Zhang Y., Li Z.-T., Huang F. (2024). Multiple Hydrogen Bonding
Driven Supramolecular Architectures and
Their Biomedical Applications. Chem. Soc. Rev..

[101] Politzer P., Murray J. S., Clark T. (2013). Halogen Bonding
and
Other *σ*-Hole Interactions: A Perspective. Phys. Chem. Chem. Phys..

[102] Clark T. (2013). *σ*-Holes. Wiley Interdiscip.
Rev. Comput. Mol. Sci..

[103] Hassel O., Ro̷mming C. (1962). Direct Structural Evidence for Weak
Charge-Transfer Bonds in Solids Containing Chemically Saturated Molecules. Q. Rev., Chem. Soc..

[104] Decato, D. A. ; John, E. A. ; Berryman, O. B. Chapter 1. Halogen Bonding: An Introduction. In Halogen Bonding in Solution, 1st ed.; Huber, S. , Ed.; Wiley, 2021; pp 1–41.

[105] Shields Z. P., Murray J. S., Politzer P. (2010). Directional
Tendencies
of Halogen and Hydrogen Bonds. Int. J. Quantum
Chem..

[106] Politzer P., Murray J. S., Clark T. (2013). Halogen Bonding and
Other *σ*-Hole Interactions: A Perspective. Phys. Chem. Chem. Phys..

[107] Brammer L. (2017). Halogen Bonding,
Chalcogen Bonding, Pnictogen Bonding,
Tetrel Bonding: Origins, Current Status and Discussion. Faraday Discuss..

[108] Mehta N., Fellowes T., White J. M., Goerigk L. (2021). CHAL336 Benchmark
Set: How Well Do Quantum-Chemical Methods Describe Chalcogen-Bonding
Interactions?. J. Chem. Theory Comput..

[109] Bulfield D., Huber S. M. (2016). Halogen Bonding
in Organic Synthesis
and Organocatalysis. Chem. Eur. J..

[110] Sutar R. L., Huber S. M. (2019). Catalysis of Organic
Reactions through
Halogen Bonding. ACS Catal..

[111] Grabowski, S. J. Hydrogen Bonds and Halogen Bonds – A Comparative Study. In 13: Hydrogen Bonds and Halogen Bonds – A Comparative Study; Novoa, J. J. , Ed.; The Royal Society of Chemistry, 2017; pp 478–515.

[112] Lu L., Lu Y., Zhu Z., Liu H. (2020). Pnictogen, Chalcogen,
and Halogen Bonds in Catalytic Systems: Theoretical Study and Detailed
Comparison. J. Mol. Model..

[113] Metrangolo P., Neukirch H., Pilati T., Resnati G. (2005). Halogen Bonding
Based Recognition Processes: A World Parallel to Hydrogen Bonding. Acc. Chem. Res..

[114] Murray J. S., Lane P., Clark T., Riley K. E., Politzer P. (2012). *σ*-Holes, *π*-Holes and Electrostatically-Driven
Interactions. J. Mol. Model..

[115] Wang H., Wang W., Jin W. J. (2016). *σ*-Hole Bond vs *π*-Hole Bond:
A Comparison Based
on Halogen Bond. Chem. Rev..

[116] Quinonero D., Garau C., Rotger C., Frontera A., Ballester P., Costa A., Deya P. M. (2002). Anion–*π* Interactions: Do They Exist?. Angew. Chem., Int. Ed..

[117] Schottel B. L., Chifotides H. T., Dunbar K. R. (2008). Anion-*π* Interactions. Chem. Soc. Rev..

[118] Egli M., Sarkhel S. (2007). Lone Pair- Aromatic Interactions:
To Stabilize or Not to Stabilize. Acc. Chem.
Res..

[119] Politzer P., Murray J. S. (2020). Electrostatics
and Polarization in *σ*-and *π*-Hole Noncovalent Interactions:
An Overview. ChemPhysChem.

[120] Martinez C. R., Iverson B. L. (2012). Rethinking the Term
“Pi-Stacking”. Chem. Sci..

[121] Bang, D. ; Tereshko, V. ; Kossiakoff, A. A. ; Kent, S. B. H. X-Ray Crystal Structure of Chemically Synthesized Crambin. 2007.

[122] Bang D., Tereshko V., Kossiakoff A. A., Kent S. B. H. (2009). Role of a Salt Bridge in the Model Protein Crambin
Explored by Chemical Protein Synthesis: X-ray Structure of a Unique
Protein Analogue, [V15A]­Crambin-*α*-Carboxamide. Mol. BioSyst..

[123] Meyer E. A., Castellano R. K., Diederich F. (2003). Interactions
with Aromatic Rings in Chemical and Biological Recognition. Angew. Chem., Int. Ed..

[124] Lee E. C., Kim D., Jurecka P., Tarakeshwar P., Hobza P., Kim K. S. (2007). Understanding of Assembly Phenomena
by Aromatic- Aromatic Interactions: Benzene Dimer and the Substituted
Systems. J. Phys. Chem. A.

[125] Ma J. C., Dougherty D. A. (1997). The Cation- *π* Interaction. Chem. Rev..

[126] Tsuzuki S. (2012). CH/*π* Interactions. Annu. Rep. Prog. Chem., Sect. C: Phys. Chem..

[127] Carter-Fenk K., Herbert J. M. (2020). Reinterpreting *π*-Stacking. Phys. Chem. Chem.
Phys..

[128] Herbert J. M. (2021). Neat, Simple,
and Wrong: Debunking Electrostatic Fallacies
Regarding Noncovalent Interactions. J. Phys.
Chem. A.

[129] Tsuzuki S., Uchimaru T., Mikami M. (2006). Intermolecular Interaction
between Hexafluorobenzene and Benzene: Ab Initio Calculations Including
CCSD­(T) Level Electron Correlation Correction. J. Phys. Chem. A.

[130] Yan L., Gloor C. J., Moran A. M., You W. (2023). Non-Covalent Interactions
Involving *π* Effect between Organic Cations
in Low-Dimensional Organic/Inorganic Hybrid Perovskites. Appl. Phys. Lett..

[131] Dougherty, D. A. Chapter 14: The Cation–*π* Interaction. In Intermolecular Interactions in Crystals: Fundamentals of Crystal Engineering; Novoa, J. J. , Ed.; The Royal Society of Chemistry, 2017; pp 516–529.

[132] Xie N.-Z., Du Q.-S., Li J.-X., Huang R.-B. (2015). Exploring
Strong Interactions in Proteins with Quantum Chemistry and Examples
of Their Applications in Drug Design. PLoS One.

[133] Dimitrijević B.
P., Borozan S. Z., Stojanović S. (2012). *π*–*π* and Cation–*π* Interactions in Protein–Porphyrin
Complex Crystal Structures. RSC Adv..

[134] Gamez P. (2014). The Anion–*π* Interaction: Naissance
and Establishment of a Peculiar Supramolecular Bond. Inorg. Chem. Front..

[135] Giese M., Albrecht M., Rissanen K. (2016). Experimental Investigation
of Anion–*π* Interactions – Applications
and Biochemical Relevance. Chem. Commun..

[136] Takahashi, O. ; Nishio, M. Chapter 12: The CH···*π* Hydrogen Bond. In Intermolecular Interactions in Crystals: Fundamentals of Crystal Engineering; Novoa, J. J. , Ed.; The Royal Society of Chemistry, 2017; pp 453–477.

[137] Marcus Y., Hefter G. (2006). Ion Pairing. Chem. Rev..

[138] Fuoss R. M. (1958). Ionic Association. III. The Equilibrium
between Ion
Pairs and Free Ions. J. Am. Chem. Soc..

[139] Bosshard H. R., Marti D. N., Jelesarov I. (2004). Protein Stabilization
by Salt Bridges: Concepts, Experimental Approaches and Clarification
of Some Misunderstandings. J. Mol. Recognit..

[140] Donald J. E., Kulp D. W., DeGrado W. F. (2011). Salt Bridges:
Geometrically
Specific, Designable Interactions. Proteins:
Struct., Funct., Bioinf..

[141] Debye P., Hückel E. (1923). Zur Theorie Der Elektrolyte. I. Gefrierpunktserniedrigung
Und Verwandte Erscheinungen. Phys. Z..

[142] Jeziorski B., Moszynski R., Szalewicz K. (1994). Perturbation
Theory Approach to Intermolecular Potential Energy Surfaces of van
Der Waals Complexes. Chem. Rev..

[143] Andrés J. (2019). Nine Questions on Energy
Decomposition Analysis. J. Comput. Chem..

[144] Kitaura K., Morokuma K. (1976). A New Energy Decomposition
Scheme
for Molecular Interactions within the Hartree-Fock Approximation. Int. J. Quantum Chem..

[145] Khaliullin R. Z., Cobar E. A., Lochan R. C., Bell A. T., Head-Gordon M. (2007). Unravelling the Origin of Intermolecular Interactions
Using Absolutely Localized Molecular Orbitals. J. Phys. Chem. A.

[146] Glendening E. D., Streitwieser A. (1994). Natural Energy Decomposition Analysis:
An Energy Partitioning Procedure for Molecular Interactions with Application
to Weak Hydrogen Bonding, Strong Ionic, and Moderate Donor–Acceptor
Interactions. J. Chem. Phys..

[147] Glendening E. D. (1996). Natural Energy Decomposition Analysis:
Explicit Evaluation
of Electrostatic and Polarization Effects with Application to Aqueous
Clusters of Alkali Metal Cations and Neutrals. J. Am. Chem. Soc..

[148] Ziegler T., Rauk A. (1979). A Theoretical Study of the Ethylene-Metal
Bond in Complexes between Copper­(1+), Silver­(1+), Gold­(1+), Platinum(0)
or Platinum­(2+) and Ethylene, Based on the Hartree-Fock-Slater Transition-State
Method. Inorg. Chem..

[149] Schneider W. B., Bistoni G., Sparta M., Saitow M., Riplinger C., Auer A. A., Neese F. (2016). Decomposition
of Intermolecular
Interaction Energies within the Local Pair Natural Orbital Coupled
Cluster Framework. J. Chem. Theory Comput..

[150] Parker T. M., Burns L. A., Parrish R. M., Ryno A. G., Sherrill C. D. (2014). Levels of symmetry adapted perturbation
theory (SAPT).
I. Efficiency and performance for interaction energies. J. Chem. Phys..

[151] Misquitta A. J., Szalewicz K. (2005). Symmetry-Adapted Perturbation-Theory
Calculations of Intermolecular Forces Employing Density-Functional
Description of Monomers. J. Chem. Phys..

[152] Misquitta A. J., Podeszwa R., Jeziorski B., Szalewicz K. (2005). Intermolecular potentials based on symmetry-adapted
perturbation theory with dispersion energies from time-dependent density-functional
calculations. J. Chem. Phys..

[153] Goodfellow, I. ; Bengio, Y. ; Courville, A. Deep Learning; MIT Press, 2016.

[154] Jordan M. I., Mitchell T. M. (2015). Machine Learning: Trends, Perspectives,
and Prospects. Science.

[155] Kotsiantis, S. B. Supervised Machine Learning: A Review of Classification Techniques. In Emerging Artificial Intelligence Applications in Computer Engineering; Maglogiannis, I. , Karpouzis, K. , Wallace, M. , Soldatos, J. , Eds.; IOS Press, 2007; Vol. 160; pp 3–24.

[156] Bishop, C. M. ; Nasrabadi, N. M. Pattern Recognition and Machine Learning; Springer, 2006; Vol. 4.

[157] Kutner, M. H. ; Nachtsheim, C. J. ; Neter, J. ; Li, W. Applied Linear Statistical Models, 5th ed.; McGraw-Hill/Irwin, 2004.

[158] Hoerl A.
E., Kennard R. W. (1970). Ridge Regression:
Biased Estimation
for Nonorthogonal Problems. Technometrics.

[159] Burges, C. J. ; Schölkopf, B. ; Smola, A. J. Advances in Kernel Methods: Support Vector Learning; The MIT Press, 1998.

[160] Cortes C., Vapnik V. (1995). Support-Vector Networks. Mach.
Learn..

[161] Chen, T. ; Guestrin, C. Xgboost: A Scalable Tree Boosting System. Proceedings of the 22nd ACM SIGKDD International Conference on Knowledge Discovery and Data Mining, 2016; pp 785–794.

[162] Abdi H., Williams L. J. (2010). Principal Component
Analysis. Wiley interdisciplinary reviews: computational
statistics.

[163] van der Maaten L., Hinton G. (2008). Visualizing
Data Using t-SNE. J. Mach. Learn. Res..

[164] Goodfellow, I. ; Pouget-Abadie, J. ; Mirza, M. ; Xu, B. ; Warde-Farley, D. ; Ozair, S. ; Courville, A. ; Bengio, Y. Generative Adversarial Nets. Adv. Neural Inf. Process. Syst. 2014, 27.

[165] Li P., Pei Y., Li J. (2023). A Comprehensive Survey on Design
and Application of Autoencoder in Deep Learning. Appl. Soft Comput..

[166] Chen, T. ; Kornblith, S. ; Norouzi, M. ; Hinton, G. A Simple Framework for Contrastive Learning of Visual Representations. International Conference on Machine Learning. 2020; pp 1597–1607.

[167] Sutton, R. S. ; Barto, A. G. Reinforcement Learning: An Introduction; The MIT Press, 2018.

[168] Mnih V. (2015). Human-Level Control
through Deep Reinforcement Learning. Nature.

[169] Watkins C. J., Dayan P. (1992). Q-Learning. Mach. Learn..

[170] Sutton, R. S. ; McAllester, D. ; Singh, S. ; Mansour, Y. Policy Gradient Methods for Reinforcement Learning with Function Approximation. Adv. Neural Inf. Process. Syst. 1999.1057

[171] Kotsiantis S. B., Kanellopoulos D., Pintelas P. E. (2007). Data Preprocessing
for Supervised Leaning. Int. J. Comput. Inf.
Eng..

[172] Domingos P. (2012). A Few Useful Things
to Know about Machine Learning. Commun. ACM.

[173] Rosenblatt F. (1958). The Perceptron: A Probabilistic Model
for Information
Storage and Organization in the Brain. Psychol.
Rev..

[174] Rumelhart D. E., Hinton G. E., Williams R. J. (1986). Learning
Representations
by Back-Propagating Errors. Nature.

[175] Bergstra, J. ; Bardenet, R. ; Bengio, Y. ; Kégl, B. Algorithms for Hyper-Parameter Optimization. Adv. Neural Inf. Process. Syst. 2011.

[176] Bergstra J., Bengio Y. (2012). Random Search for Hyper-Parameter
Optimization. J. Mach. Learn. Res..

[177] Snoek, J. ; Larochelle, H. ; Adams, R. P. Practical Bayesian Optimization of Machine Learning Algorithms. Adv. Neural Inf. Process. Syst. 2012, 25.

[178] Maclaurin, D. ; Duvenaud, D. ; Adams, R. Gradient-Based Hyperparameter Optimization through Reversible Learning. International Conference on Machine Learning, 2015; pp 2113–2122.

[179] Real, E. ; Aggarwal, A. ; Huang, Y. ; Le, Q. V. Regularized Evolution for Image Classifier Architecture Search. Proceedings of the Aaai Conference on Artificial Intelligence. 2019; pp 4780–4789.

[180] Bebis G., Georgiopoulos M. (1994). Feed-Forward Neural Networks. Ieee Potentials.

[181] Li Z., Liu F., Yang W., Peng S., Zhou J. (2022). A Survey of
Convolutional Neural Networks: Analysis, Applications, and Prospects. IEEE Trans. Neural Netw. Learn. Syst..

[182] LeCun Y., Bottou L., Bengio Y., Haffner P. (1998). Gradient-based
learning applied to document recognition. Proceedings
of the IEEE.

[183] Krizhevsky, A. ; Sutskever, I. ; Hinton, G. E. Imagenet classification with deep convolutional neural networks. Advances in neural information processing systems 2012, 25.

[184] Jiménez J., Skalic M., Martinez-Rosell G., De Fabritiis G. (2018). K deep: protein–ligand
absolute binding affinity
prediction via 3d-convolutional neural networks. J. Chem. Inf. Model..

[185] Ragoza M., Hochuli J., Idrobo E., Sunseri J., Koes D. R. (2017). Protein–Ligand Scoring with Convolutional Neural
Networks. J. Chem. Inf. Model..

[186] Wallach, I. ; Dzamba, M. ; Heifets, A. AtomNet: a deep convolutional neural network for bioactivity prediction in structure-based drug discovery. arXiv preprint arXiv:1510.02855 2015, 10.48550/arXiv.1510.02855.

[187] Wu Z., Pan S., Chen F., Long G., Zhang C., Yu P. S. (2021). A Comprehensive
Survey on Graph Neural Networks. IEEE Trans.
Neural Netw. Learn. Syst..

[188] Gilmer, J. ; Schoenholz, S. S. ; Riley, P. F. ; Vinyals, O. ; Dahl, G. E. Neural Message Passing for Quantum Chemistry. International Conference on Machine Learning, 2017; pp 1263–1272.

[189] Kipf, T. N. ; Welling, M. Semi-Supervised Classification with Graph Convolutional Networks. International Conference on Learning Representations. 2017.

[190] Geerts, F. ; Mazowiecki, F. ; Perez, G. Let’s Agree to Degree: Comparing Graph Convolutional Networks in the Message-Passing Framework. Proceedings of the 38th International Conference on Machine Learning, 2021; pp 3640–3649.

[191] Heid E., Greenman K. P., Chung Y., Li S.-C., Graff D. E., Vermeire F. H., Wu H., Green W. H., McGill C. J. (2024). Chemprop: A Machine Learning Package
for Chemical Property
Prediction. J. Chem. Inf. Model..

[192] Vaswani, A. ; Shazeer, N. ; Parmar, N. ; Uszkoreit, J. ; Jones, L. ; Gomez, A. N. ; Kaiser, Ł. ; Polosukhin, I. Attention Is All You Need. Adv. Neural Inf. Process. Syst. 2017.

[193] Jozefowicz, R. ; Zaremba, W. ; Sutskever, I. An Empirical Exploration of Recurrent Network Architectures. International Conference on Machine Learning, 2015; pp 2342–2350.

[194] Bohacek R. S., McMartin C., Guida W. C. (1996). The Art and Practice
of Structure-Based Drug Design: A Molecular Modeling Perspective. Med. Res. Rev..

[195] Schwaller P., Vaucher A. C., Laplaza R., Bunne C., Krause A., Corminboeuf C., Laino T. (2022). Machine Intelligence
for Chemical Reaction Space. Wiley Interdiscip.
Rev. Comput. Mol. Sci..

[196] Bird C. L., Frey J. G. (2013). Chemical information matters: an
e-Research perspective on information and data sharing in the chemical
sciences. Chem. Soc. Rev..

[197] Mercado R., Kearnes S. M., Coley C. W. (2023). Data Sharing
in
Chemistry: Lessons Learned and a Case for Mandating Structured Reaction
Data. J. Chem. Inf. Model..

[198] Holtrop, F. ; Visscher, K. W. ; Jupp, A. R. ; Slootweg, J. C. Steric Attraction: A Force to Be Reckoned With. In Adv. Phys. Org. Chem.; Elsevier, 2020; Vol. 54; pp 119–141.

[199] Priya Gnanasekar, S. ; Arunan, E. Chapter 7: Molecular Beam and Spectroscopic Techniques: Towards Fundamental Understanding of Intermolecular Interactions/Bonds. In Intermolecular Interactions in Crystals: Fundamentals of Crystal Engineering; Novoa, J. J. , Ed.; The Royal Society of Chemistry, 2017; pp 259–309.

[200] Vioglio, P. C. ; Chierotti, M. R. ; Gobetto, R. Chapter 8: Solid-state NMR Techniques for the Study of Intermolecular Interactions. In Intermolecular Interactions in Crystals: Fundamentals of Crystal Engineering; Novoa, J. J. , Ed.; The Royal Society of Chemistry, 2017; pp 310–349.

[201] Chapter 2. Characteristics of Non-covalent Complexes and Their Determination by Experimental and Theoretical Techniques. In Theoretical and Computational Chemistry Series; Royal Society of Chemistry: Cambridge, 2009; pp 21–69.

[202] Šponer J., Leszczynski J., Hobza P. (2001). Hydrogen Bonding, Stacking
and Cation Binding of DNA Bases. J. Mol. Struct.
THEOCHEM.

[203] Hobza P., Šponer J., Reschel T. (1995). Density Functional
Theory and Molecular Clusters. J. Comput. Chem..

[204] Kristyán S., Pulay P. (1994). Can (Semi)­Local Density
Functional
Theory Account for the London Dispersion Forces?. Chem. Phys. Lett..

[205] Pérez-Jordá J. M, Becke A. (1995). A Density-Functional
Study of van Der Waals Forces: Rare Gas Diatomics. Chem. Phys. Lett..

[206] Janowski T., Pulay P. (2007). High Accuracy Benchmark Calculations
on the Benzene Dimer Potential Energy Surface. Chem. Phys. Lett..

[207] Sinnokrot M. O., Valeev E. F., Sherrill C. D. (2002). Estimates of the
Ab Initio Limit for *π*-*π* Interactions: The Benzene Dimer. J. Am. Chem.
Soc..

[208] Raghavachari K., Trucks G. W., Pople J. A., Head-Gordon M. (1989). A Fifth-Order
Perturbation Comparison of Electron Correlation Theories. Chem. Phys. Lett..

[209] Kodrycka M., Patkowski K. (2019). Platinum, Gold, and Silver Standards
of Intermolecular Interaction Energy Calculations. J. Chem. Phys..

[210] Patkowski, K. Chapter One - Benchmark Databases of Intermolecular Interaction Energies: Design, Construction, and Significance. In Annual Reports in Computational Chemistry; Elsevier, 2017; Vol. 13; pp 3–91.

[211] Řezáč J., Hobza P. (2016). Benchmark Calculations
of Interaction Energies in Noncovalent Complexes and Their Applications. Chem. Rev..

[212] Goerigk L., Hansen A., Bauer C., Ehrlich S., Najibi A., Grimme S. (2017). A Look at the Density Functional
Theory Zoo with the Advanced GMTKN55 Database for General Main Group
Thermochemistry, Kinetics and Noncovalent Interactions. Phys. Chem. Chem. Phys..

[213] Mardirossian N., Head-Gordon M. (2017). Thirty Years of Density Functional
Theory in Computational Chemistry: An Overview and Extensive Assessment
of 200 Density Functionals. Mol. Phys..

[214] Mardirossian N., Head-Gordon M. (2016). *ω*B97M-V: A
Combinatorially Optimized, Range-Separated Hybrid, Meta-GGA Density
Functional with VV10 Nonlocal Correlation. J.
Chem. Phys..

[215] Prasad V. K., Otero-de-la-Roza A., DiLabio G. A. (2022). Fast and Accurate
Quantum Mechanical Modeling of Large Molecular Systems Using Small
Basis Set Hartree–Fock Methods Corrected with Atom-Centered
Potentials. J. Chem. Theory Comput..

[216] Al-Hamdani Y. S., Nagy P. R., Zen A., Barton D., Kállay M., Brandenburg J. G., Tkatchenko A. (2021). Interactions
between Large Molecules Pose a Puzzle for Reference Quantum Mechanical
Methods. Nat. Commun..

[217] Al-Hamdani Y. S., Tkatchenko A. (2019). Understanding
Non-Covalent Interactions
in Larger Molecular Complexes from First Principles. J. Chem. Phys..

[218] Hermann J., DiStasio R. A., Tkatchenko A. (2017). First-Principles
Models for van Der Waals Interactions in Molecules and Materials:
Concepts, Theory, and Applications. Chem. Rev..

[219] Caldeweyher E., Bannwarth C., Grimme S. (2017). Extension of the D3
Dispersion Coefficient Model. J. Chem. Phys..

[220] Grimme S., Antony J., Ehrlich S., Krieg H. (2010). A Consistent
and Accurate *Ab Initio* Parametrization of Density
Functional Dispersion Correction (DFT-D) for the 94 Elements H-Pu. J. Chem. Phys..

[221] Grimme S. (2004). Accurate Description of van Der Waals
Complexes by
Density Functional Theory Including Empirical Corrections. J. Comput. Chem..

[222] Grimme S., Ehrlich S., Goerigk L. (2011). Effect of the Damping
Function in Dispersion Corrected Density Functional Theory. J. Comput. Chem..

[223] Grimme S. (2006). Semiempirical
GGA-type Density Functional Constructed
with a Long-Range Dispersion Correction. J.
Comput. Chem..

[224] Grimme S., Hansen A., Ehlert S., Mewes J.-M. (2021). r2SCAN-3c:
A “Swiss Army Knife” Composite Electronic-Structure
Method. J. Chem. Phys..

[225] Riplinger C., Neese F. (2013). An Efficient and near
Linear Scaling
Pair Natural Orbital Based Local Coupled Cluster Method. J. Chem. Phys..

[226] Ambrosetti A., Reilly A. M., DiStasio R. A., Tkatchenko A. (2014). Long-Range
Correlation Energy Calculated from Coupled Atomic Response Functions. J. Chem. Phys..

[227] Stöhr M., Tkatchenko A. (2019). Quantum mechanics
of proteins in
explicit water: The role of plasmon-like solute-solvent interactions. Sci. Adv..

[228] Dion M., Rydberg H., Schroder E., Langreth D. C., Lundqvist B. I. (2004). Van Der Waals Density Functional for General Geometries. Phys. Rev. Lett..

[229] Vydrov O.
A., Van Voorhis T. (2010). Nonlocal van
Der Waals Density Functional:
The Simpler the Better. J. Chem. Phys..

[230] Jones A. P., Crain J., Sokhan V. P., Whitfield T. W., Martyna G. J. (2013). Quantum Drude oscillator model of
atoms and molecules:
Many-body polarization and dispersion interactions for atomistic simulation. Phys. Rev. B.

[231] Khabibrakhmanov A., Fedorov D. V., Tkatchenko A. (2023). Universal
Pairwise Interatomic van der Waals Potentials Based on Quantum Drude
Oscillators. J. Chem. Theory Comput..

[232] Řezáč J., Hobza P. (2013). Describing
Noncovalent
Interactions beyond the Common Approximations: How Accurate Is the
”Gold Standard,” CCSD­(T) at the Complete Basis Set Limit?. J. Chem. Theory Comput..

[233] Karton A., Spackman P. R. (2021). Evaluation of density functional
theory for a large and diverse set of organic and inorganic equilibrium
structures. J. Comput. Chem..

[234] Zhao C., Wu R., Zhang S., Hong X. (2023). Benchmark
Study of Density Functional Theory Methods in Geometry Optimization
of Transition Metal–Dinitrogen Complexes. J. Phys. Chem. A.

[235] Grimme S., Steinmetz M. (2013). Effects of London dispersion correction
in density functional theory on the structures of organic molecules
in the gas phase. Phys. Chem. Chem. Phys..

[236] Risthaus T., Steinmetz M., Grimme S. (2014). Implementation of nuclear
gradients of range-separated hybrid density functionals and benchmarking
on rotational constants for organic molecules. J. Comput. Chem..

[237] Karton A., de Oliveira M. T. (2025). Good Practices in Database Generation
for Benchmarking Density Functional Theory. Wiley Interdiscip. Rev. Comput. Mol. Sci..

[238] Hohenstein E. G., Sherrill C. D. (2012). Wavefunction methods
for noncovalent
interactions. Wiley Interdiscip. Rev. Comput.
Mol. Sci..

[239] Aspuru-Guzik A., Lindh R., Reiher M. (2018). The Matter Simulation
(R)­evolution. ACS Cent. Sci..

[240] Amezcua M., Setiadi J., Mobley D. L. (2024). The SAMPL9
Host–Guest
Blind Challenge: An Overview of Binding Free Energy Predictive Accuracy. Phys. Chem. Chem. Phys..

[241] Sedlak R., Janowski T., Pitoňák M., Řezáč J., Pulay P., Hobza P. (2013). Accuracy of
Quantum Chemical Methods for Large Noncovalent Complexes. J. Chem. Theory Comput..

[242] Grimme S. (2012). Supramolecular
Binding Thermodynamics by Dispersion-Corrected
Density Functional Theory. Chem. Eur. J..

[243] Sure R., Grimme S. (2015). Comprehensive Benchmark
of Association
(Free) Energies of Realistic Host–Guest Complexes. J. Chem. Theory Comput..

[244] Gorges J., Grimme S., Hansen A. (2022). Reliable Prediction
of Association (Free) Energies of Supramolecular Complexes with Heavy
Main Group Elements – the HS13L Benchmark Set. Phys. Chem. Chem. Phys..

[245] Kruse H., Mladek A., Gkionis K., Hansen A., Grimme S., Sponer J. (2015). Quantum Chemical Benchmark Study
on 46 RNA Backbone Families Using a Dinucleotide Unit. J. Chem. Theory Comput..

[246] Spicher S., Caldeweyher E., Hansen A., Grimme S. (2021). Benchmarking
London Dispersion Corrected Density Functional Theory for Noncovalent
Ion–*π* Interactions. Phys. Chem. Chem. Phys..

[247] Lao K. U., Schäffer R., Jansen G., Herbert J. M. (2015). Accurate
Description of Intermolecular Interactions Involving Ions Using Symmetry-Adapted
Perturbation Theory. J. Chem. Theory Comput..

[248] Ernst B. G., Lao K. U., Sullivan A. G., DiStasio R. A. (2020). Attracting Opposites: Promiscuous
Ion-*π* Binding in the Nucleobases. J. Phys. Chem.
A.

[249] Medrano Sandonas L., Van Rompaey D., Fallani A., Hilfiker M., Hahn D., Perez-Benito L., Verhoeven J., Tresadern G., Kurt Wegner J., Ceulemans H., Tkatchenko A. (2024). Dataset for
Quantum-Mechanical Exploration of Conformers
and Solvent Effects in Large Drug-like Molecules. Sci. Data.

[250] Pollice R., Chen P. (2019). Origin of the Immiscibility
of Alkanes
and Perfluoroalkanes. J. Am. Chem. Soc..

[251] Vik E. C., Li P., Pellechia P. J., Shimizu K. D. (2019). Transition-State Stabilization by N→*π** Interactions Measured Using Molecular Rotors. J. Am. Chem. Soc..

[252] Lin B., Liu H., Karki I., Vik E. C., Smith M. D., Pellechia P. J., Shimizu K. D. (2023). Pnictogen Interactions with Nitrogen
Acceptors. Angew. Chem., Int. Ed..

[253] Yang L., Brazier J. B., Hubbard T. A., Rogers D. M., Cockroft S. L. (2016). Can Dispersion Forces Govern Aromatic
Stacking in an
Organic Solvent?. Angew. Chem., Int. Ed..

[254] Pollice R., Bot M., Kobylianskii I. J., Shenderovich I., Chen P. (2017). Attenuation of London Dispersion
in Dichloromethane Solutions. J. Am. Chem. Soc..

[255] Wilming F. M., Marazzi B., Debes P. P., Becker J., Schreiner P. R. (2023). Probing the Size Limit of Dispersion
Energy Donors
with a Bifluorenylidene Balance: Magic Cyclohexyl. J. Org. Chem..

[256] Wang L., Wu Y., Deng Y., Kim B., Pierce L., Krilov G., Lupyan D., Robinson S., Dahlgren M. K., Greenwood J. (2015). Accurate and Reliable
Prediction of Relative Ligand Binding Potency in Prospective Drug
Discovery by Way of a Modern Free-Energy Calculation Protocol and
Force Field. J. Am. Chem. Soc..

[257] Schindler C. E. M. (2020). Large-Scale Assessment
of Binding Free
Energy Calculations in Active Drug Discovery Projects. J. Chem. Inf. Model..

[258] Calbo J., Ortí E., Sancho-García J. C., Aragó J. (2015). Accurate Treatment of Large Supramolecular Complexes
by Double-Hybrid Density Functionals Coupled with Nonlocal van Der
Waals Corrections. J. Chem. Theory Comput..

[259] Pracht P., Bohle F., Grimme S. (2020). Automated
Exploration
of the Low-Energy Chemical Space with Fast Quantum Chemical Methods. Phys. Chem. Chem. Phys..

[260] Xie L., Liu H. (2002). The Treatment of Solvation by a Generalized Born Model
and a Self-Consistent Charge-Density Functional Theory-Based Tight-Binding
Method. J. Comput. Chem..

[261] Ringe S., Oberhofer H., Hille C., Matera S., Reuter K. (2016). Function-Space-Based
Solution Scheme for the Size-Modified
Poisson–Boltzmann Equation in Full-Potential DFT. J. Chem. Theory Comput..

[262] Cametti M., Crousse B., Metrangolo P., Milani R., Resnati G. (2012). The Fluorous Effect in Biomolecular
Applications. Chem. Soc. Rev..

[263] Newberry R. W., Raines R. T. (2017). The N→*π** Interaction. Acc. Chem. Res..

[264] Nikitin K., O’Gara R. (2019). Mechanisms
and beyond: Elucidation
of Fluxional Dynamics by Exchange NMR Spectroscopy. Chem. Eur. J..

[265] Lunazzi L., Mancinelli M., Mazzanti A., Lepri S., Ruzziconi R., Schlosser M. (2012). Rotational Barriers of Biphenyls
Having Heavy Heteroatoms as Ortho-Substituents: Experimental and Theoretical
Determination of Steric Effects. Org. Biomol.
Chem..

[266] Thordarson P. (2011). Determining
Association Constants from Titration Experiments
in Supramolecular Chemistry. Chem. Soc. Rev..

[267] Pastor A., Martínez-Viviente E. (2008). NMR Spectroscopy
in
Coordination Supramolecular Chemistry: A Unique and Powerful Methodology. Coord. Chem. Rev..

[268] Bastos M., Abian O., Johnson C. M., Ferreira-da-Silva F., Vega S., Jimenez-Alesanco A., Ortega-Alarcon D., Velazquez-Campoy A. (2023). Isothermal Titration Calorimetry. Nat. Rev. Methods Primers.

[269] Rodgers M. T., Armentrout P. B. (2000). Noncovalent Metal–Ligand Bond
Energies as Studied by Threshold Collision-Induced Dissociation. Mass Spectrom. Rev..

[270] Cappel D., Hall M. L., Lenselink E. B., Beuming T., Qi J., Bradner J., Sherman W. (2016). Relative Binding
Free Energy Calculations Applied to Protein Homology Models. J. Chem. Inf. Model..

[271] Díaz Mirón J. E. Z., Stein M. (2022). A Benchmark for Non-Covalent
Interactions in Organometallic Crystals. Phys.
Chem. Chem. Phys..

[272] Otero-de-la-Roza A., Johnson E. R. (2012). A Benchmark for Non-Covalent Interactions
in Solids. J. Chem. Phys..

[273] Reilly A. M., Tkatchenko A. (2013). Understanding
the Role of Vibrations,
Exact Exchange, and Many-Body van Der Waals Interactions in the Cohesive
Properties of Molecular Crystals. J. Chem. Phys..

[274] Bernardes C. E. S., Joseph A. (2015). Evaluation of the OPLS-AA
Force Field
for the Study of Structural and Energetic Aspects of Molecular Organic
Crystals. J. Phys. Chem. A.

[275] Schmidt L., Van Der Spoel D., Walz M.-M. (2023). Probing Phase Transitions
in Organic Crystals Using Atomistic MD Simulations. ACS Phys. Chem. Au..

[276] Reilly A. M., Tkatchenko A. (2013). Seamless and Accurate Modeling of
Organic Molecular Materials. J. Phys. Chem.
Lett..

[277] Moellmann J., Grimme S. (2014). DFT-D3 Study
of Some Molecular Crystals. J. Phys. Chem. C.

[278] Dolgonos G. A., Hoja J., Boese A. D. (2019). Revised
Values for
the X23 Benchmark Set of Molecular Crystals. Phys. Chem. Chem. Phys..

[279] Kříž K., Řezáč J. (2022). Non-Covalent
Interactions Atlas Benchmark Data Sets 4: *σ*-Hole Interactions. Phys. Chem. Chem. Phys..

[280] Řezáč J. (2020). Non-Covalent Interactions
Atlas Benchmark
Data Sets 2: Hydrogen Bonding in an Extended Chemical Space. J. Chem. Theory Comput..

[281] Řezáč J. (2022). Non-Covalent Interactions Atlas Benchmark
Data Sets 5: London Dispersion in an Extended Chemical Space. Phys. Chem. Chem. Phys..

[282] Karton A., Daon S., Martin J. M. (2011). W4–11: A
High-Confidence Benchmark Dataset for Computational Thermochemistry
Derived from First-Principles W4 Data. Chem.
Phys. Lett..

[283] Kříž K., Nováček M., Řezáč J. (2021). Non-Covalent
Interactions Atlas Benchmark
Data Sets 3: Repulsive Contacts. J. Chem. Theory
Comput..

[284] Bauzá A., Alkorta I., Frontera A., Elguero J. (2013). On the Reliability
of Pure and Hybrid DFT Methods for the Evaluation of Halogen, Chalcogen,
and Pnicogen Bonds Involving Anionic and Neutral Electron Donors. J. Chem. Theory Comput..

[285] De Azevedo
Santos L., Ramalho T. C., Hamlin T. A., Bickelhaupt F. M. (2021). Chalcogen
Bonds: Hierarchical *Ab Initio* Benchmark and Density
Functional Theory Performance Study. J. Comput.
Chem..

[286] Liu M., Han X., Chen H., Peng Q., Huang H. (2023). A Molecular
Descriptor of Intramolecular Noncovalent Interaction for Regulating
Optoelectronic Properties of Organic Semiconductors. Nat. Commun..

[287] Setiawan D., Kraka E., Cremer D. (2015). Strength of
the Pnicogen
Bond in Complexes Involving Group Va Elements N, P, and As. J. Phys. Chem. A.

[288] Palanisamy D. (2020). A Quantum
Chemical Perspective on the Potency of Electron
Donors and Acceptors in Pnicogen Bonds (As···N, P···N,
N···N). J. Mol. Model..

[289] Karton A., Martin J. M. L. (2012). Explicitly Correlated
W*n* Theory: W1-F12 and W2-F12. J. Chem. Phys..

[290] Řezáč, J. Non-Covalent Interactions Atlas. 2024; http://www.nciatlas.org (accessed February 23, 2025).

[291] Parker T.
M., Burns L. A., Parrish R. M., Ryno A. G., Sherrill C. D. (2014). Levels of Symmetry
Adapted Perturbation Theory (SAPT).
I. Efficiency and Performance for Interaction Energies. J. Chem. Phys..

[292] Sparrow Z. M., Ernst B. G., Joo P. T., Lao K. U., DiStasio R. A. (2021). NENCI-2021.
I. A Large Benchmark Database of Non-Equilibrium
Non-Covalent Interactions Emphasizing Close Intermolecular Contacts. J. Chem. Phys..

[293] Christensen A. S., Sirumalla S. K., Qiao Z., O’Connor M. B., Smith D. G. A., Ding F., Bygrave P. J., Anandkumar A., Welborn M., Manby F. R., Miller T. F. (2021). OrbNet Denali: A
Machine Learning Potential for Biological and Organic Chemistry with
Semi-Empirical Cost and DFT Accuracy. J. Chem.
Phys..

[294] Isert C., Atz K., Jiménez-Luna J., Schneider G. (2022). QMugs, Quantum Mechanical Properties of Drug-like Molecules. Sci. Data.

[295] Masumian E., Boese A. D. (2024). Benchmarking Swaths
of Intermolecular
Interaction Components with Symmetry-Adapted Perturbation Theory. J. Chem. Theory Comput..

[296] Donchev A. G., Taube A. G., Decolvenaere E., Hargus C., McGibbon R. T., Law K.-H., Gregersen B. A., Li J.-L., Palmo K., Siva K., Bergdorf M., Klepeis J. L., Shaw D. E. (2021). Quantum Chemical Benchmark Databases
of Gold-Standard Dimer Interaction Energies. Sci. Data.

[297] Eastman, P. ; Behara, P. K. ; Dotson, D. ; Galvelis, R. ; Herr, J. ; Horton, J. ; Mao, Y. ; Chodera, J. ; Pritchard, B. ; Wang, Y. ; De Fabritiis, G. ; Markland, T. SPICE 2.0.1. 2024; https://zenodo.org/records/10975225 (accessed February 23, 2025).

[298] Eastman P., Behara P. K., Dotson D. L., Galvelis R., Herr J. E., Horton J. T., Mao Y., Chodera J. D., Pritchard B. P., Wang Y., De Fabritiis G., Markland T. E. (2023). SPICE, A Dataset
of Drug-like Molecules and Peptides
for Training Machine Learning Potentials. Sci.
Data.

[299] Unke O. T., Stöhr M., Ganscha S., Unterthiner T., Maennel H., Kashubin S., Ahlin D., Gastegger M., Medrano Sandonas L., Berryman J. T., Tkatchenko A., Müller K.-R. (2024). Biomolecular
Dynamics with Machine-Learned Quantum-Mechanical
Force Fields Trained on Diverse Chemical Fragments. Sci. Adv..

[300] Unke, O. T. DFT data for “Biomolecular Dynamics with Machine Learned Quantum-Mechanical Force Fields Trained on Diverse Chemical Fragments”. 2024; https://zenodo.org/records/10720941 (accessed March 16, 2025). 10.1126/sciadv.adn4397 PMC11809612 38579003

[301] Kabylda, A. ; Frank, J. T. ; Dou, S. S. ; Khabibrakhmanov, A. ; Sandonas, L. M. ; Unke, O. T. ; Chmiela, S. ; Müller, K.-R. ; Tkatchenko, A. Molecular Simulations with a Pretrained Neural Network and Universal Pairwise Force Fields. ChemRxiv 2024, 10.26434/chemrxiv-2024-bdfr0-v2. PMC12447504 40886167

[302] Kabylda, A. ; Frank, J. T. ; Dou, S. S. ; Khabibrakhmanov, A. ; Sandonas, L. M. ; Unke, O. T. ; Chmiela, S. ; Müller, K.-R. ; Tkatchenko, A. DFT data for ”Molecular Simulations with a Pretrained Neural Network and Universal Pairwise Force Fields”. 2025; https://zenodo.org/records/14779793 (accessed March 13, 2025). 10.1021/jacs.5c09558 PMC12447504 40886167

[303] Spronk S. A., Glick Z. L., Metcalf D. P., Sherrill C. D., Cheney D. L. (2023). A Quantum
Chemical Interaction Energy Dataset for Accurately
Modeling Protein-Ligand Interactions. Sci. Data.

[304] Berman H. M., Westbrook J., Feng Z., Gilliland G., Bhat T. N., Weissig H., Shindyalov I. N., Bourne P. E. (2000). The Protein Data Bank. Nucleic
Acids Res..

[305] Berman H., Henrick K., Nakamura H., Markley J. L. (2007). The Worldwide
Protein Data Bank (wwPDB): Ensuring a Single, Uniform Archive of PDB
Data. Nucleic Acids Res..

[306] Burns L. A., Faver J. C., Zheng Z., Marshall M. S., Smith D. G. A., Vanommeslaeghe K., MacKerell A. D., Merz K. M., Sherrill C. D. (2017). The BioFragment
Database (BFDb):
An Open-Data Platform for Computational Chemistry Analysis of Noncovalent
Interactions. J. Chem. Phys..

[307] Siebenmorgen T., Menezes F., Benassou S., Merdivan E., Didi K., Mourão A. S. D., Kitel R., Liò P., Kesselheim S., Piraud M., Theis F. J., Sattler M., Popowicz G. M. (2024). MISATO: Machine Learning Dataset of Protein–Ligand
Complexes for Structure-Based Drug Discovery. Nat. Comput. Sci..

[308] Korlepara D. B. (2024). PLAS-20k: Extended Dataset of Protein-Ligand
Affinities from MD Simulations for Machine Learning Applications. Sci. Data.

[309] Řezáč J., Riley K. E., Hobza P. (2011). S66: A Well-balanced
Database of Benchmark Interaction Energies Relevant to Biomolecular
Structures. J. Chem. Theory Comput..

[310] Wang Q., Rackers J. A., He C., Qi R., Narth C., Lagardere L., Gresh N., Ponder J. W., Piquemal J.-P., Ren P. (2015). General Model for Treating Short-Range
Electrostatic Penetration in a Molecular Mechanics Force Field. J. Chem. Theory Comput..

[311] Jurečka P., Šponer J., Černý J., Hobza P. (2006). Benchmark Database of Accurate (MP2
and CCSD­(T) Complete Basis Set
Limit) Interaction Energies of Small Model Complexes, DNA Base Pairs,
and Amino Acid Pairs. Phys. Chem. Chem. Phys..

[312] Gaulton A. (2017). The ChEMBL Database
in 2017. Nucleic Acids Res..

[313] Bannwarth C., Ehlert S., Grimme S. (2019). GFN2-xTB—An
Accurate and Broadly Parametrized Self-Consistent Tight-Binding Quantum
Chemical Method with Multipole Electrostatics and Density-Dependent
Dispersion Contributions. J. Chem. Theory Comput..

[314] Atz K., Isert C., Böcker M. N. A., Jiménez-Luna J., Schneider G. (2022). *Δ*-Quantum Machine-Learning for
Medicinal Chemistry. Phys. Chem. Chem. Phys..

[315] Goerigk L., Grimme S. (2010). A General Database
for Main Group
Thermochemistry, Kinetics, and Noncovalent Interactions - Assessment
of Common and Reparameterized (*meta*-)­GGA Density
Functionals. J. Chem. Theory Comput..

[316] Goerigk L., Grimme S. (2011). Efficient and Accurate
Double-Hybrid-Meta-GGA
Density Functionals—Evaluation with the Extended GMTKN30 Database
for General Main Group Thermochemistry, Kinetics, and Noncovalent
Interactions. J. Chem. Theory Comput..

[317] Mehta N., Casanova-Páez M., Goerigk L. (2018). Semi-Empirical
or Non-Empirical Double-Hybrid Density Functionals: Which Are More
Robust?. Phys. Chem. Chem. Phys..

[318] Najibi A., Goerigk L. (2018). The Nonlocal Kernel
in van Der Waals
Density Functionals as an Additive Correction: An Extensive Analysis
with Special Emphasis on the B97M-V and *ω*B97M-V
Approaches. J. Chem. Theory Comput..

[319] Řezáč J. (2020). Non-Covalent Interactions
Atlas Benchmark
Data Sets: Hydrogen Bonding. J. Chem. Theory
Comput..

[320] Řezáč J., Riley K. E., Hobza P. (2012). Benchmark
Calculations of Noncovalent Interactions of Halogenated Molecules. J. Chem. Theory Comput..

[321] Kesharwani M. K., Manna D., Sylvetsky N., Martin J. M. L. (2018). The X40×10
Halogen Bonding Benchmark Revisited:
Surprising Importance of (*n*–1)­d Subvalence
Correlation. J. Phys. Chem. A.

[322] McGibbon R. T., Taube A. G., Donchev A. G., Siva K., Hernández F., Hargus C., Law K.-H., Klepeis J. L., Shaw D. E. (2017). Improving the Accuracy of Mo̷ller-Plesset
Perturbation
Theory with Neural Networks. J. Chem. Phys..

[323] Frank J. T., Unke O. T., Müller K.-R., Chmiela S. (2024). A Euclidean Transformer for Fast and Stable Machine
Learned Force Fields. Nat. Commun..

[324] Hoja J., Medrano Sandonas L., Ernst B. G., Vazquez-Mayagoitia A., DiStasio R. A., Tkatchenko A. (2021). QM7-X, a Comprehensive
Dataset of
Quantum-Mechanical Properties Spanning the Chemical Space of Small
Organic Molecules. Sci. Data.

[325] Burley, S. K. ; Berman, H. M. ; Kleywegt, G. J. ; Markley, J. L. ; Nakamura, H. ; Velankar, S. Protein Data Bank (PDB): The Single Global Macromolecular Structure Archive. In Protein Crystallography; Wlodawer, A. , Dauter, Z. , Jaskolski, M. , Eds.; Springer New York: New York, NY, 2017; Vol. 1607; pp 627–641. 10.1007/978-1-4939-7000-1_26 PMC5823500 28573592

[326] Varadi M., Anyango S., Deshpande M., Nair S., Natassia C., Yordanova G., Yuan D., Stroe O., Wood G., Laydon A. (2022). AlphaFold Protein Structure Database: Massively Expanding
the Structural
Coverage of Protein-Sequence Space with High-Accuracy Models. Nucleic Acids Res..

[327] Wang R., Fang X., Lu Y., Yang C.-Y., Wang S. (2005). The PDBbind Database: Methodologies and Updates. J. Med. Chem..

[328] Korlepara D. B., Vasavi C. S., Jeurkar S., Pal P. K., Roy S., Mehta S., Sharma S., Kumar V., Muvva C., Sridharan B., Garg A., Modee R., Bhati A. P., Nayar D., Priyakumar U. D. (2022). PLAS-5k: Dataset of Protein-Ligand
Affinities from Molecular Dynamics for Machine Learning Applications. Sci. Data.

[329] Ward M. D. (1997). Photo-Induced Electron and Energy
Transfer in Non-Covalently
Bonded Supramolecular Assemblies. Chem. Soc.
Rev..

[330] Vallavoju N., Sivaguru J. (2014). Supramolecular Photocatalysis: Combining
Confinement and Non-Covalent Interactions to Control Light Initiated
Reactions. Chem. Soc. Rev..

[331] Haque A., Alenezi K. M., Khan M. S., Wong W.-Y., Raithby P. R. (2023). Non-Covalent Interactions (NCIs)
in *π*-Conjugated Functional Materials: Advances
and Perspectives. Chem. Soc. Rev..

[332] Wasielewski M. R. (2009). Self-Assembly Strategies for Integrating
Light Harvesting
and Charge Separation in Artificial Photosynthetic Systems. Acc. Chem. Res..

[333] Dreuw A., Head-Gordon M. (2005). Single-Reference ab Initio Methods
for the Calculation of Excited States of Large Molecules. Chem. Rev..

[334] Lischka H., Nachtigallová D., Aquino A. J. A., Szalay P. G., Plasser F., Machado F. B. C., Barbatti M. (2018). Multireference
Approaches for Excited States of Molecules. Chem. Rev..

[335] Crespo-Hernández C. E., Cohen B., Hare P. M., Kohler B. (2004). Ultrafast Excited-State
Dynamics in Nucleic Acids. Chem. Rev..

[336] Hancock A. C., Goerigk L. (2023). Noncovalently Bound
Excited-State
Dimers: A Perspective on Current Time-Dependent Density Functional
Theory Approaches Applied to Aromatic Excimer Models. RSC Adv..

[337] Jones A. C., Goerigk L. (2024). Exploring Non-Covalent Interactions
in Excited States: Beyond Aromatic Excimer Models. Phys. Chem. Chem. Phys..

[338] Ge Q., Mao Y., Head-Gordon M. (2018). Energy Decomposition Analysis for
Exciplexes Using Absolutely Localized Molecular Orbitals. J. Chem. Phys..

[339] Ji L., Shi J., Wei J., Yu T., Huang W. (2020). Air-Stable
Organic Radicals: New-generation Materials for Flexible Electronics?. Adv. Mater..

[340] Mizuno A., Matsuoka R., Mibu T., Kusamoto T. (2024). Luminescent
Radicals. Chem. Rev..

[341] Hatakeyama-Sato K., Oyaizu K. (2023). Redox: Organic Robust
Radicals and
Their Polymers for Energy Conversion/Storage Devices. Chem. Rev..

[342] Yan M., Kawamata Y., Baran P. S. (2017). Synthetic Organic
Electrochemical
Methods since 2000: On the Verge of a Renaissance. Chem. Rev..

[343] Zhu C., Ang N. W., Meyer T. H., Qiu Y., Ackermann L. (2021). Organic Electrochemistry:
Molecular Syntheses with Potential. ACS Cent.
Sci..

[344] Romero N. A., Nicewicz D. A. (2016). Organic Photoredox Catalysis. Chem. Rev..

[345] Chan A. Y., Perry I. B., Bissonnette N. B., Buksh B. F., Edwards G. A., Frye L. I., Garry O. L., Lavagnino M. N., Li B. X., Liang Y. (2022). Metallaphotoredox:
The Merger of Photoredox and Transition Metal Catalysis. Chem. Rev..

[346] Klein A. V., Hambley T. W. (2009). Platinum Drug Distribution in Cancer
Cells and Tumors. Chem. Rev..

[347] Rottenberg S., Disler C., Perego P. (2021). The Rediscovery
of
Platinum-Based Cancer Therapy. Nat. Rev. Cancer..

[348] Blomberg M. R., Borowski T., Himo F., Liao R.-Z., Siegbahn P. E. (2014). Quantum Chemical Studies of Mechanisms
for Metalloenzymes. Chem. Rev..

[349] Pyykkö P. (1997). Strong Closed-Shell Interactions
in Inorganic Chemistry. Chem. Rev..

[350] Feinberg E. N., Sur D., Wu Z., Husic B. E., Mai H., Li Y., Sun S., Yang J., Ramsundar B., Pande V. S. (2018). PotentialNet for
Molecular Property Prediction. ACS Cent. Sci..

[351] Ballester P. J., Mitchell J. B. O. (2010). A Machine Learning
Approach to Predicting
Protein–Ligand Binding Affinity with Applications to Molecular
Docking. Bioinformatics.

[352] Li H., Leung K.-S., Wong M.-H., Ballester P. J. (2015). Improving
AutoDock Vina Using Random Forest: The Growing Accuracy of Binding
Affinity Prediction by the Effective Exploitation of Larger Data Sets. Mol. Inform..

[353] Wang R., Lai L., Wang S. (2002). Further Development
and Validation of Empirical Scoring Functions for Structure-Based
Binding Affinity Prediction. J. Comput. Aided
Mol. Des..

[354] Cang Z., Wei G.-W. (2017). TopologyNet: Topology Based Deep
Convolutional and Multi-Task Neural Networks for Biomolecular Property
Predictions. PLoS Comput. Biol..

[355] Cho H., Lee E. K., Choi I. S. (2020). Layer-Wise
Relevance Propagation
of InteractionNet Explains Protein–Ligand Interactions at the
Atom Level. Sci. Rep..

[356] Bach S., Binder A., Montavon G., Klauschen F., Müller K.-R., Samek W. (2015). On Pixel-Wise Explanations for Non-Linear
Classifier Decisions by Layer-Wise Relevance Propagation. PLoS One.

[357] Xiao F., Cheng Y., Wang J.-R., Wang D., Zhang Y., Chen K., Mei X., Luo X. (2022). Cocrystal
Prediction of Bexarotene by Graph Convolution Network and Bioavailability
Improvement. Pharmaceutics.

[358] Torng W., Altman R. B. (2019). Graph Convolutional Neural Networks
for Predicting Drug-Target Interactions. J.
Chem. Inf. Model..

[359] Moskal M., Beker W., Szymkuć S., Grzybowski B. A. (2021). Scaffold-Directed Face Selectivity Machine-Learned
from Vectors of Non-covalent Interactions. Angew.
Chem., Int. Ed..

[360] Orlandi M., Coelho J. A. S., Hilton M. J., Toste F. D., Sigman M. S. (2017). Parametrization of Non-covalent Interactions for Transition
State Interrogation Applied to Asymmetric Catalysis. J. Am. Chem. Soc..

[361] Li X., Liu P., Houk K. N., Birman V. B. (2008). Origin of Enantioselectivity
in CF_3_-PIP-Catalyzed Kinetic Resolution of Secondary Benzylic
Alcohols. J. Am. Chem. Soc..

[362] Neel A. J., Milo A., Sigman M. S., Toste F. D. (2016). Enantiodivergent
Fluorination of Allylic Alcohols: Data Set Design Reveals Structural
Interplay between Achiral Directing Group and Chiral Anion. J. Am. Chem. Soc..

[363] Glendening E. D., Streitwieser A. (1994). Natural Energy Decomposition Analysis:
An Energy Partitioning Procedure for Molecular Interactions with Application
to Weak Hydrogen Bonding, Strong Ionic, and Moderate Donor–Acceptor
Interactions. J. Chem. Phys..

[364] Morokuma K. (1971). Molecular Orbital Studies of Hydrogen
Bonds. III. C
= O···H–O Hydrogen Bond in H_2_CO···H_2_O and H_2_CO···2H_2_O. J. Chem. Phys..

[365] Ziegler T., Rauk A. (1979). A Theoretical Study of the Ethylene-Metal
Bond in Complexes between Copper­(1+), Silver­(1+), Gold­(1+), Platinum(0)
or Platinum­(2+) and Ethylene, Based on the Hartree-Fock-Slater Transition-State
Method. Inorg. Chem..

[366] Ziegler T., Rauk A. (1979). Carbon Monoxide, Carbon
Monosulfide,
Molecular Nitrogen, Phosphorus Trifluoride, and Methyl Isocyanide
as.Sigma. Donors and.Pi. Acceptors. A Theoretical Study by the Hartree-Fock-Slater
Transition-State Method. Inorg. Chem..

[367] Ziegler T., Rauk A. (1977). On the Calculation
of Bonding Energies
by the Hartree Fock Slater Method: I. The Transition State Method. Theor. Chim. Acta.

[368] DeTar D. F. (1987). General
Definition of Formal Steric Enthalpy and Applications
to Estimating Differences of Enthalpies of Formation. J. Org. Chem..

[369] Ramachandran S., Kota P., Ding F., Dokholyan N. V. (2011). Automated
Minimization of Steric Clashes in Protein Structures. Proteins: Struct., Funct., Bioinf..

[370] Liu S. (2007). Steric Effect: A Quantitative Description
from Density Functional
Theory. J. Chem. Phys..

[371] Tsirelson V. G., Stash A. I., Liu S. (2010). Quantifying Steric
Effect with Experimental Electron Density. J.
Chem. Phys..

[372] Zhang J., He X., Wang B., Rong C., Zhao D., Liu S. (2024). Density-Based Quantification
of Steric
Effects: Validation by Taft Steric Parameters from Acid-Catalyzed
Hydrolysis of Esters. Phys. Chem. Chem. Phys..

[373] Tolman C. A. (1970). Phosphorus Ligand Exchange Equilibriums
on Zerovalent
Nickel. Dominant Role for Steric Effects. J.
Am. Chem. Soc..

[374] Hillier A. C., Sommer W. J., Yong B. S., Petersen J. L., Cavallo L., Nolan S. P. (2003). A Combined Experimental and Theoretical
Study Examining the Binding of *N* -Heterocyclic Carbenes
(NHC) to the Cp∗RuCl (Cp∗ = *η*
^5^ -C _5_ Me _5_) Moiety: Insight into
Stereoelectronic Differences between Unsaturated and Saturated NHC
Ligands. Organometallics.

[375] Poater A., Cosenza B., Correa A., Giudice S., Ragone F., Scarano V., Cavallo L. (2009). Samb *V* ca: A Web Application for the Calculation of the Buried
Volume of
N-Heterocyclic Carbene Ligands. Eur. J. Inorg.
Chem..

[376] Verloop, A. ; Hoogenstraaten, W. ; Tipker, J. Development and Application of New Steric Substituent Parameters in Drug Design. In Drug Design; Elsevier, 1976; pp 165–207.

[377] Verloop, A. THE STERIMOL APPROACH: FURTHER DEVELOPMENT OF THE METHOD AND NEW APPLICATIONS. In Pesticide Chemistry: Human Welfare and Environment; Elsevier, 1983; pp 339–344.

[378] Brethomé A. V., Fletcher S. P., Paton R. S. (2019). Conformational Effects
on Physical-Organic Descriptors: The Case of Sterimol Steric Parameters. ACS Catal..

[379] Exploring QSAR: Fundamentals and Applications in Chemistry and Biology; Hansch, C. , Leo, A. , Hoekman, D. H. , Eds.; ACS Professional Reference Book; American Chemical Society: Washington, DC, 1995.

[380] Winstein S., Holness N. J. (1955). Neighboring Carbon and Hydrogen.
XIX. t-Butylcyclohexyl Derivatives. Quantitative Conformational Analysis. J. Am. Chem. Soc..

[381] Adams R., Yuan H. C. (1933). The Stereochemistry of Diphenyls
and Analogous Compounds. Chem. Rev..

[382] Bott G., Field L. D., Sternhell S. (1980). Steric Effects.
A Study of a Rationally Designed System. J.
Am. Chem. Soc..

[383] Taft R. W. (1952). Linear Free Energy Relationships from Rates of Esterification
and Hydrolysis of Aliphatic and Ortho-substituted Benzoate Esters. J. Am. Chem. Soc..

[384] Taft R. W. (1953). Linear Steric Energy Relationships. J. Am. Chem. Soc..

[385] Taft R. W. (1952). Polar and Steric Substituent Constants for Aliphatic
and O-Benzoate Groups from Rates of Esterification and Hydrolysis
of Esters. J. Am. Chem. Soc..

[386] Charton M. (1975). Steric Effects I. Esterification
and Acid-Catalyzed
Hydrolysis of Esters. J. Am. Chem. Soc..

[387] Charton M. (1975). Steric Effects. II. Base-catalyzed
Ester Hydrolysis. J. Am. Chem. Soc..

[388] Kutter E., Hansch C. (1969). Steric Parameters in
Drug Design.
Monoamine Oxidase Inhibitors and Antihistamines. J. Med. Chem..

[389] Miller J. J., Sigman M. S. (2008). Quantitatively Correlating the Effect
of Ligand-Substituent Size in Asymmetric Catalysis Using Linear Free
Energy Relationships. Angew. Chem., Int. Ed..

[390] Mantilli L., Gérard D., Torche S., Besnard C., Mazet C. (2010). Improved Catalysts
for the Iridium-Catalyzed Asymmetric Isomerization
of Primary Allylic Alcohols Based on Charton Analysis. Chem. Eur. J..

[391] Quintard A., Alexakis A. (2011). 1,2-Sulfone Rearrangement in Organocatalytic
Reactions. Org. Biomol. Chem..

[392] Gustafson J. L., Sigman M. S., Miller S. J. (2010). Linear
Free-Energy
Relationship Analysis of a Catalytic Desymmetrization Reaction of
a Diarylmethane-bis­(Phenol). Org. Lett..

[393] Harper K. C., Bess E. N., Sigman M. S. (2012). Multidimensional
Steric Parameters in the Analysis of Asymmetric Catalytic Reactions. Nat. Chem..

[394] Ardkhean R., Roth P. M. C., Maksymowicz R. M., Curran A., Peng Q., Paton R. S., Fletcher S. P. (2017). Enantioselective
Conjugate Addition Catalyzed by a Copper Phosphoramidite Complex:
Computational and Experimental Exploration of Asymmetric Induction. ACS Catal..

[395] Ardkhean R., Mortimore M., Paton R. S., Fletcher S. P. (2018). Formation
of Quaternary Centres by Copper Catalysed Asymmetric Conjugate Addition
to *β*-Substituted Cyclopentenones with the Aid
of a Quantitative Structure–Selectivity Relationship. Chem. Sci..

[396] Milo A., Neel A. J., Toste F. D., Sigman M. S. (2015). A Data-Intensive
Approach to Mechanistic Elucidation Applied to Chiral Anion Catalysis. Science.

[397] Clements H. D., Flynn A. R., Nicholls B. T., Grosheva D., Lefave S. J., Merriman M. T., Hyster T. K., Sigman M. S. (2023). Using Data
Science for Mechanistic Insights and Selectivity Predictions in a
Non-Natural Biocatalytic Reaction. J. Am. Chem.
Soc..

[398] Haas B. C., Goetz A. E., Bahamonde A., McWilliams J. C., Sigman M. S. (2022). Predicting Relative Efficiency of
Amide Bond Formation Using Multivariate Linear Regression. Proc. Natl. Acad. Sci. U.S.A..

[399] Reid J. P., Sigman M. S. (2019). Holistic Prediction
of Enantioselectivity
in Asymmetric Catalysis. Nature.

[400] Samha M. H., Wahlman J. L. H., Read J. A., Werth J., Jacobsen E. N., Sigman M. S. (2022). Exploring Structure–Function
Relationships of Aryl Pyrrolidine-Based Hydrogen-Bond Donors in Asymmetric
Catalysis Using Data-Driven Techniques. ACS
Catal..

[401] Santiago C. B., Guo J.-Y., Sigman M. S. (2018). Predictive and Mechanistic
Multivariate Linear Regression Models for Reaction Development. Chem. Sci..

[402] Werth J., Sigman M. S. (2020). Connecting and Analyzing Enantioselective
Bifunctional Hydrogen Bond Donor Catalysis Using Data Science Tools. J. Am. Chem. Soc..

[403] Werth J., Sigman M. S. (2021). Linear Regression Model Development
for Analysis of Asymmetric Copper-Bisoxazoline Catalysis. ACS Catal..

[404] Solel E., Ruth M., Schreiner P. R. (2021). London
Dispersion Helps Refine Steric A-Values: Dispersion Energy Donor Scales. J. Am. Chem. Soc..

[405] Pollice R., Chen P. (2019). Origin of the Immiscibility of Alkanes
and Perfluoroalkanes. J. Am. Chem. Soc..

[406] Pollice R., Chen P. (2019). A Universal Quantitative
Descriptor
of the Dispersion Interaction Potential. Angew.
Chem., Int. Ed..

[407] Orlandi M., Hilton M. J., Yamamoto E., Toste F. D., Sigman M. S. (2017). Mechanistic Investigations of the Pd(0)-Catalyzed Enantioselective
1,1-Diarylation of Benzyl Acrylates. J. Am.
Chem. Soc..

[408] Lu T., Chen Q. (2020). van der Waals
potential: an important complement to
molecular electrostatic potential in studying intermolecular interactions. J. Mol. Model..

[409] Kayal R., Baldinelli L., Harden I., Neese F., Bistoni G. (2025). Understanding
and quantifying the impact of solute–solvent
van der Waals interactions on the selectivity of asymmetric catalytic
transformations. Chem. Sci..

[410] Clements A., Lewis M. (2006). Arene-Cation Interactions
of Positive
Quadrupole Moment Aromatics and Arene-Anion Interactions of Negative
Quadrupole Moment Aromatics. J. Phys. Chem.
A.

[411] Mecozzi S., West A. P., Dougherty D. A. (1996). Cation-*π* Interactions in Simple Aromatics: Electrostatics
Provide a Predictive Tool. J. Am. Chem. Soc..

[412] Bauzá A., Deyà P. M., Frontera A., Quiñonero D. (2014). Substituent
Effects in Cation–*π* Interactions Revisited:
A General Approach Based on Intrinsic Properties of the Arenes. Phys. Chem. Chem. Phys..

[413] Liu Z., Chen Z., Xi J., Xu X. (2020). An Accurate Single
Descriptor for Ion–*π* Interactions. Natl. Sci. Rev..

[414] Stone A. J. (2013). Are Halogen Bonded Structures Electrostatically Driven?. J. Am. Chem. Soc..

[415] Ai-Guo Z. (2017). Dissecting
the Nature of Halogen Bonding Interactions
from Energy Decomposition and Wavefunction Analysis. Monatsh. Chem..

[416] Herrmann B., Svatunek D. (2024). Directionality of Halogen-Bonds:
Insights from 2D Energy Decomposition Analysis. Chem. - Asian J..

[417] Murray J. S., Politzer P. (2011). The Electrostatic Potential: An Overview. Wiley Interdiscip. Rev. Comput. Mol. Sci..

[418] Gonthier J. F., Steinmann S. N., Wodrich M. D., Corminboeuf C. (2012). Quantification
of “Fuzzy” Chemical Concepts: A Computational Perspective. Chem. Soc. Rev..

[419] Alkorta I., Sánchez-Sanz G., Elguero J. (2013). Linear Free Energy
Relationships in Halogen Bonds. CrystEngComm.

[420] Politzer P., Murray J. S. (2013). Halogen Bonding:
An Interim Discussion. ChemPhysChem.

[421] Lange A., Heidrich J., Zimmermann M. O., Exner T. E., Boeckler F. M. (2019). Scaffold Effects on Halogen Bonding
Strength. J. Chem. Inf. Model..

[422] Heidrich J., Exner T. E., Boeckler F. M. (2019). Predicting
the Magnitude
of *σ*-Holes Using VmaxPred, a Fast and Efficient
Tool Supporting the Application of Halogen Bonds in Drug Discovery. J. Chem. Inf. Model..

[423] Devore D. P., Shuford K. L. (2024). Data and Molecular Fingerprint-Driven
Machine Learning Approaches to Halogen Bonding. J. Chem. Inf. Model..

[424] Gao, T. ; Li, H. ; Li, W. ; Li, L. ; Fang, C. ; Li, H. ; Hu, L. ; Lu, Y. ; Su, Z.-M. A Machine Learning Correction for DFT Non-Covalent Interactions Based on the S22, S66 and X40 Benchmark Databases. J. Cheminf. 2016, 8. 10.1186/s13321-016-0133-7. PMC4855356 27148408

[425] Gráfová L., Pitoňák M., Řezáč J., Hobza P. (2010). Comparative Study of
Selected Wave Function and Density Functional Methods for Noncovalent
Interaction Energy Calculations Using the Extended S22 Data Set. J. Chem. Theory Comput..

[426] Řezáč J., Riley K. E., Hobza P. (2011). Extensions
of the S66 Data Set: More Accurate Interaction Energies and Angular-Displaced
Nonequilibrium Geometries. J. Chem. Theory Comput..

[427] Peccati F., Desmedt E., Contreras-García J. (2019). A Regression
Approach to Accurate Interaction Energies Using Topological Descriptors. Comput. Theor. Chem..

[428] Spackman M. A., Maslen E. N. (1986). Chemical Properties from the Promolecule. J. Phys. Chem..

[429] Johnson E. R., Keinan S., Mori-Sánchez P., Contreras-García J., Cohen A. J., Yang W. (2010). Revealing
Noncovalent Interactions. J. Am. Chem. Soc..

[430] Boto R. A., Peccati F., Laplaza R., Quan C., Carbone A., Piquemal J.-P., Maday Y., Contreras-García J. (2020). NCIPLOT4:
Fast, Robust, and Quantitative Analysis of Noncovalent Interactions. J. Chem. Theory Comput..

[431] Wieduwilt E. K., Boto R. A., Macetti G., Laplaza R., Contreras-García J., Genoni A. (2023). Extracting Quantitative
Information at Quantum Mechanical Level from Noncovalent Interaction
Index Analyses. J. Chem. Theory Comput..

[432] Cheng Y.-Y., Yuan H. (2006). Quantitative Study
of Electrostatic
and Steric Effects on Physicochemical Property and Biological Activity. J. Mol. Graphics Modell..

[433] Kier L. B., Hall L. H. (1990). An Electrotopological-State
Index
for Atoms in Molecules. Pharm. Res..

[434] Zimmermann M. O., Lange A., Wilcken R., Cieslik M. B., Exner T. E., Joerger A. C., Koch P., Boeckler F. M. (2014). Halogen-Enriched
Fragment Libraries as Chemical Probes for Harnessing Halogen Bonding
in Fragment-Based Lead Discovery. Future Med.
Chem..

[435] Pedregosa F. (2011). Scikit-Learn:
Machine Learning in Python. J. Mach. Learn.
Res..

[436] Rogers D., Hahn M. (2010). Extended-Connectivity
Fingerprints. J. Chem. Inf. Model..

[437] Zhong S., Guan X. (2023). Count-Based Morgan
Fingerprint: A
More Efficient and Interpretable Molecular Representation in Developing
Machine Learning-Based Predictive Regression Models for Water Contaminants’
Activities and Properties. Environ. Sci. Technol..

[438] Tabet A., Gebhart T., Wu G., Readman C., Pierson Smela M., Rana V. K., Baker C., Bulstrode H., Anikeeva P., Rowitch D. H., Scherman O. A. (2020). Applying
Support-Vector
Machine Learning Algorithms toward Predicting Host–Guest Interactions
with Cucurbit[7]­Uril. Phys. Chem. Chem. Phys..

[439] Wang D., Li W., Dong X., Li H., Hu L. (2023). TFRegNCI: Interpretable Noncovalent Interaction Correction
Multimodal
Based on Transformer Encoder Fusion. J. Chem.
Inf. Model..

[440] Selvaraju, R. R. ; Cogswell, M. ; Das, A. ; Vedantam, R. ; Parikh, D. ; Batra, D. Grad-CAM: Visual Explanations from Deep Networks via Gradient-Based Localization. 2017 IEEE International Conference on Computer Vision (ICCV), 2017; pp 618–626.

[441] Li W., Wang D., Yang Z., Zhang H., Hu L., Chen G. (2022). DeepNCI: DFT Noncovalent
Interaction Correction with Transferable
Multimodal Three-Dimensional Convolutional Neural Networks. J. Chem. Inf. Model..

[442] Radosavovic, I. ; Kosaraju, R. P. ; Girshick, R. ; He, K. ; Dollár, P. Designing Network Design Spaces. 2020 IEEE/CVF Conference on Computer Vision and Pattern Recognition (CVPR), 2020; pp 10425–10433.

[443] Dosovitskiy, A. ; Beyer, L. ; Kolesnikov, A. ; Weissenborn, D. ; Zhai, X. ; Unterthiner, T. ; Dehghani, M. ; Minderer, M. ; Heigold, G. ; Gelly, S. ; Uszkoreit, J. ; Houlsby, N. An Image Is Worth 16 × 16 Words: Transformers for Image Recognition at Scale. International Conference on Learning Representations, 2021.

[444] Unke O. T., Chmiela S., Sauceda H. E., Gastegger M., Poltavsky I., Schütt K. T., Tkatchenko A., Müller K.-R. (2021). Machine Learning Force Fields. Chem. Rev..

[445] Glick Z. L., Metcalf D. P., Koutsoukas A., Spronk S. A., Cheney D. L., Sherrill C. D. (2020). AP-Net: An Atomic-Pairwise
Neural Network for Smooth and Transferable Interaction Potentials. J. Chem. Phys..

[446] Brooks B. R. (2009). CHARMM: The Biomolecular Simulation Program. J. Comput. Chem..

[447] Ponder J. W., Case D. A. (2003). Force Fields for Protein Simulations. Adv. Protein. Chem..

[448] Harder E. (2016). OPLS3: A Force Field Providing Broad Coverage
of Drug-like
Small Molecules and Proteins. J. Chem. Theory
Comput..

[449] Konrad M., Wenzel W. (2021). CONI-Net: Machine Learning of Separable
Intermolecular Force Fields. J. Chem. Theory
Comput..

[450] Bereau T., DiStasio R. A., Tkatchenko A., Von Lilienfeld O. A. (2018). Non-Covalent
Interactions across Organic and Biological
Subsets of Chemical Space: Physics-based Potentials Parametrized from
Machine Learning. J. Chem. Phys..

[451] Schriber J. B., Nascimento D. R., Koutsoukas A., Spronk S. A., Cheney D. L., Sherrill C. D. (2021). CLIFF:
A Component-Based,
Machine-Learned, Intermolecular Force Field. J. Chem. Phys..

[452] Behler J. (2015). Constructing High-Dimensional Neural
Network Potentials:
A Tutorial Review. Int. J. Quantum Chem..

[453] Behler J., Parrinello M. (2007). Generalized
Neural-Network Representation
of High-Dimensional Potential-Energy Surfaces. Phys. Rev. Lett..

[454] Schmid N., Eichenberger A. P., Choutko A., Riniker S., Winger M., Mark A. E., Van Gunsteren W. F. (2011). Definition
and Testing of the GROMOS Force-Field Versions 54A7 and 54B7. Eur. Biophys. J..

[455] Temelso B., Archer K. A., Shields G. C. (2011). Benchmark Structures
and Binding Energies of Small Water Clusters with Anharmonicity Corrections. J. Phys. Chem. A.

[456] Huang B., Von Lilienfeld O. A. (2016). Communication: Understanding Molecular
Representations in Machine Learning: The Role of Uniqueness and Target
Similarity. J. Chem. Phys..

[457] Huang B., Von Lilienfeld O. A. (2020). Quantum Machine Learning Using Atom-in-Molecule-Based
Fragments Selected on the Fly. Nat. Chem..

[458] Smith D. G. A., Burns L. A., Patkowski K., Sherrill C. D. (2016). Revised Damping Parameters for the D3 Dispersion Correction
to Density Functional Theory. J. Phys. Chem.
Lett..

[459] Burns L. A., Mayagoitia Á.
V., Sumpter B. G., Sherrill C. D. (2011). Density-Functional Approaches to Noncovalent Interactions:
A Comparison of Dispersion Corrections (DFT-D), Exchange-Hole Dipole
Moment (XDM) Theory, and Specialized Functionals. J. Chem. Phys..

[460] Marshall M. S., Burns L. A., Sherrill C. D. (2011). Basis Set Convergence
of the Coupled-Cluster Correction, *δ*MP2CCSD­(T):
Best Practices for Benchmarking Non-Covalent Interactions and the
Attendant Revision of the S22, NBC10, HBC6, and HSG Databases. J. Chem. Phys..

[461] Chen J.-A., Chao S. D. (2023). Intermolecular Non-Bonded
Interactions
from Machine Learning Datasets. Molecules.

[462] Cheng Y., Gong Y., Liu Y., Song B., Zou Q. (2021). Molecular Design in Drug Discovery:
A Comprehensive Review of Deep
Generative Models. Briefings Bioinf..

[463] Pang C., Qiao J., Zeng X., Zou Q., Wei L. (2024). Deep Generative Models in de Novo Drug Molecule Generation. J. Chem. Inf. Model..

[464] Du Y., Jamasb A. R., Guo J., Fu T., Harris C., Wang Y., Duan C., Liò P., Schwaller P., Blundell T. L. (2024). Machine Learning-Aided Generative
Molecular Design. Nat. Mach. Intell..

[465] Parrilla-Gutiérrez J. M., Granda J. M., Ayme J.-F., Bajczyk M. D., Wilbraham L., Cronin L. (2024). Electron Density-Based
GPT for Optimization and Suggestion of Host–Guest Binders. Nat. Comput. Sci..

[466] Lagona J., Mukhopadhyay P., Chakrabarti S., Isaacs L. (2005). The Cucurbit [*n*]­Uril Family. Angew. Chem., Int. Ed..

[467] Han M., Engelhard D. M., Clever G. H. (2014). Self-Assembled Coordination Cages
Based on Banana-Shaped Ligands. Chem. Soc. Rev..

[468] Ruddigkeit L., Van Deursen R., Blum L. C., Reymond J.-L. (2012). Enumeration
of 166 Billion Organic Small Molecules in the Chemical Universe Database
GDB-17. J. Chem. Inf. Model..

[469] Ramakrishnan R., Dral P. O., Rupp M., Von Lilienfeld O. A. (2014). Quantum
Chemistry Structures and Properties of 134 Kilo Molecules. Sci. Data.

[470] Weininger D. (1988). SMILES, a Chemical Language and Information System.
1. Introduction to Methodology and Encoding Rules. J. Chem. Inf. Comput. Sci..

[471] Weininger D., Weininger A., Weininger J. L. (1989). SMILES.
2. Algorithm for Generation of Unique SMILES Notation. J. Chem. Inf. Comput. Sci..

[472] Abadi, M. ; . TensorFlow: Large-scale Machine Learning on Heterogeneous Systems. arXiv 2016, 10.48550/arXiv.1603.04467.

[473] Sherrill C. D., Manolopoulos D. E., Martínez T. J., Michaelides A. (2020). Electronic Structure Software. J. Chem. Phys..

[474] Laplaza R., Peccati F., Boto R. A., Quan C., Carbone A., Piquemal J.-P., Maday Y., Contreras-García J. (2021). NCIPLOT and
the Analysis of Noncovalent Interactions Using the Reduced Density
Gradient. Wiley Interdiscip. Rev. Comput. Mol.
Sci..

[475] Lu T. (2024). A Comprehensive Electron Wavefunction
Analysis Toolbox for Chemists,
Multiwfn. J. Chem. Phys..

[476] Poater A., Cosenza B., Correa A., Giudice S., Ragone F., Scarano V., Cavallo L. (2009). SambVca: A
Web Application
for the Calculation of the Buried Volume of n-Heterocyclic Carbene
Ligands. Eur. J. Inorg. Chem..

[477] Falivene L., Credendino R., Poater A., Petta A., Serra L., Oliva R., Scarano V., Cavallo L. (2016). SambVca 2.
A Web Tool for Analyzing Catalytic Pockets with Topographic Steric
Maps. Organometallics.

[478] Paszke, A. ; . PyTorch: An Imperative Style, High-Performance Deep Learning Library. Adv. Neural Inf. Process. Syst. 32: Annual Conference on Neural Information Processing Systems 2019, NeurIPS 2019, December 8–14, 2019, Vancouver, BC, Canada, 2019; pp 8024–8035.

[479] Wigh D. S., Goodman J. M., Lapkin A. A. (2022). A Review of Molecular
Representation in the Age of Machine Learning. Wiley Interdiscip. Rev. Comput. Mol. Sci..

[480] RDKit: Open-Source Cheminformatics Software. https://www.rdkit.org (accessed February 23, 2025).

[481] DeepChem. https://deepchem.io (accessed February 23, 2025).

[482] QML: A Python Toolkit for Quantum Machine Learning. https://www.qmlcode.org (accessed February 23, 2025).

[483] Contreras-García J., Johnson E. R., Keinan S., Chaudret R., Piquemal J.-P., Beratan D. N., Yang W. (2011). NCIPLOT: A
Program for Plotting Noncovalent Interaction Regions. J. Chem. Theory Comput..

[484] Poater A., Cosenza B., Correa A., Giudice S., Ragone F., Scarano V., Cavallo L. (2009). Samb*V* ca: A Web
Application for the Calculation of the Buried Volume of
N-Heterocyclic Carbene Ligands. Eur. J. Inorg.
Chem..

[485] Falivene L., Cao Z., Petta A., Serra L., Poater A., Oliva R., Scarano V., Cavallo L. (2019). Towards the
Online Computer-Aided Design of Catalytic Pockets. Nat. Chem..

[486] DBSTEP: DFT-based Steric Parameters. https://github.com/patonlab/DBSTEP (accessed February 23, 2025).

[487] MORFEUS: Molecular Features for Machine Learning. https://digital-chemistry-laboratory.github.io/morfeus (accessed February 23, 2025).

[488] Bannwarth C., Caldeweyher E., Ehlert S., Hansen A., Pracht P., Seibert J., Spicher S., Grimme S. (2021). Extended Tight-Binding
Quantum Chemistry Methods. Wiley Interdiscip.
Rev. Comput. Mol. Sci..

[489] Hermann G., Pohl V., Tremblay J. C., Paulus B., Hege H.-C., Schild A. (2016). ORBKIT: A Modular Python Toolbox
for Cross-Platform Postprocessing of Quantum Chemical Wavefunction
Data. J. Comput. Chem..

[490] Cronin, L. Code and the model for Electron Density-Based GPT for Optimisation and Suggestion of Host-Guest Binders, 2024; https://zenodo.org/records/10530598 (accessed February 23, 2025). 10.1038/s43588-024-00602-x PMC10965440 38459272

[491] Dou B., Zhu Z., Merkurjev E., Ke L., Chen L., Jiang J., Zhu Y., Liu J., Zhang B., Wei G.-W. (2023). Machine Learning
Methods for Small Data Challenges
in Molecular Science. Chem. Rev..

[492] Sanchez-Lengeling B., Aspuru-Guzik A. (2018). Inverse Molecular
Design Using Machine
Learning: Generative Models for Matter Engineering. Science.

[493] Müller-Dethlefs K., Hobza P. (2000). Noncovalent Interactions:
A Challenge for Experiment and Theory. Chem.
Rev..

[494] Liu X., Zhang F., Hou Z., Mian L., Wang Z., Zhang J., Tang J. (2023). Self-Supervised
Learning: Generative
or Contrastive. IEEE Trans. Knowl. Data Eng..

[495] Bommasani, R. ; On the Opportunities and Risks of Foundation Models. arXiv 2022, 10.48550/arXiv.2108.07258.

[496] Anstine D. M., Isayev O. (2023). Generative Models as an Emerging
Paradigm in the Chem. Sci.s. J. Am. Chem. Soc..

[497] Moult J., Pedersen J. T., Judson R., Fidelis K. (1995). A Large-Scale
Experiment to Assess Protein Structure Prediction Methods. Proteins: Struct., Funct., Bioinf..

